# Community guidelines for GPCR ligand bias: IUPHAR review 32

**DOI:** 10.1111/bph.15811

**Published:** 2022-03-27

**Authors:** Peter Kolb, Terry Kenakin, Stephen P. H. Alexander, Marcel Bermudez, Laura M. Bohn, Christian S. Breinholt, Michel Bouvier, Stephen J. Hill, Evi Kostenis, Kirill A. Martemyanov, Rick R. Neubig, H. Ongun Onaran, Sudarshan Rajagopal, Bryan L. Roth, Jana Selent, Arun K. Shukla, Martha E. Sommer, David E. Gloriam

**Affiliations:** 1 Department of Pharmaceutical Chemistry, Philipps-University Marburg, Marburg, Germany; 2 Department of Pharmacology, University of North Carolina School of Medicine, Chapel Hill, North Carolina, USA; 3 School of Life Sciences, University of Nottingham, Nottingham, UK; 4 Department of Pharmaceutical and Medicinal Chemistry, University of Münster, Münster, Germany; 5 Department of Molecular Medicine, The Scripps Research Institute, Jupiter, Florida, USA; 6 Department of Drug Design and Pharmacology, University of Copenhagen, Copenhagen, Denmark; 7 Department of Biochemistry and Molecular Medicine, Institute for Research in Immunology and Cancer, Université de Montréal, Québec, Canada; 8 Molecular, Cellular, and Pharmacobiology Section, Institute for Pharmaceutical Biology, University of Bonn, Bonn, Germany; 9 Department of Neuroscience, The Scripps Research Institute, Jupiter, Florida, USA; 10 Department of Pharmacology and Toxicology, Michigan State University, East Lansing, Michigan, USA; 11 Molecular Biology and Technology Development Unit, Department of Pharmacology, Faculty of Medicine, Ankara University, Ankara, Turkey; 12 Department of Medicine, Duke University Medical Center, Durham, North Carolina, USA; 13 Department of Biochemistry, Duke University Medical Center, Durham, North Carolina, USA; 14 Research Programme on Biomedical Informatics, Hospital Del Mar Medical Research Institute, Department of Experimental and Health Sciences, Pompeu Fabra University, Barcelona, Spain; 15 Department of Biological Sciences and Bioengineering, Indian Institute of Technology, Kanpur, India; 16 Institute of Medical Physics and Biophysics, Charité–Universitätsmedizin Berlin, Berlin, Germany; 17 Signalling Platform, ISAR Bioscience Institute, Munich-Planegg, Germany

## Abstract

GPCRs modulate a plethora of physiological processes and mediate the effects of one-third of FDA-approved drugs. Depending on which ligand activates a receptor, it can engage different intracellular transducers. This ‘biased signalling’ paradigm requires that we now characterize physiological signalling not just by receptors but by ligand–receptor pairs. Ligands eliciting biased signalling may constitute better drugs with higher efficacy and fewer adverse effects. However, ligand bias is very complex, making reproducibility and description challenging. Here, we provide guidelines and terminology for any scientists to design and report ligand bias experiments. The guidelines will aid consistency and clarity, as the basic receptor research and drug discovery communities continue to advance our understanding and exploitation of ligand bias. Scientific insight, biosensors, and analytical methods are still evolving and should benefit from and contribute to the implementation of the guidelines, together improving translation from in vitro to disease-relevant in vivo models.

## 1 Introduction

The ~800 human GPCRs transduce sensory inputs and systemic signals into appropriate cellular responses in numerous physiological processes. They recognize a vast diversity of signals ranging from photons, tastants and odours to ions, neurotransmitters, hormones, and cytokines (Harding et al., 2021; Wacker, Stevens, & Roth, 2017). Even though GPCRs represent the primary target of 34% of FDA-approved drugs, more than 220 non-olfactory GPCRs have disease associations which are as yet untapped in clinical research (Hauser, Attwood, Rask-Andersen, Schioth, & Gloriam, 2017; Sriram & Insel, 2018). Despite the diversity of extracellular ligands and physiological roles of GPCRs, these cell surface receptors share a conserved molecular fold and intracellular transducers. Agonist binding stabilizes active conformations of the receptor, facilitating the binding of one or more cytosolic transducer proteins. These include the heterotrimeric G proteins consisting of α, β and γ subunits that dissociate to α and βγ upon activation by the receptor. G proteins comprise 16 distinct α subunits and are divided into four families based on homology and associated downstream signalling pathways: G_s_ (G_s_ and G_olf_), G_i/o_ (G_i1_, G_i2_, G_i3_, G_O_, G_t1_, G_t2_, G_t3_, and G_z_), G_q/11_ (G_q_, G_11_, G_14_ and G_15_), and G_12/13_ (G_12_ and G_13_). Moreover, there are five different β and 12 γ subunit types, resulting in a vast number of possible heterotrimeric G protein combinations (Hillenbrand et al., 2015; Masuho et al., 2021; Milligan & Kostenis, 2006; Olsen et al., 2020).

Activated GPCRs are also bound and phosphorylated at multiple Ser and Thr residues by one or more of the seven different GPCR kinases (GRKs) or effector kinases, such as PKA and PKC. Receptor phosphorylation by GRKs is a key functional determinant for the binding of arrestin proteins (4 subtypes) (Komolov & Benovic, 2018), which can ‘arrest’ signalling by blocking G protein coupling and facilitating receptor internalization, although phosphorylation-independent arrestin interactions have also been described (Eichel et al., 2018). Arrestins are scaffold proteins that recruit and/or assemble other proteins that signal (Ahn, Shenoy, Luttrell, & Lefkowitz, 2020). Ultimately, the functional interplay between G proteins, GRKs, other kinases, arrestins, and other interaction partners at a GPCR shapes the outcome of receptor signalling in space and time (Gutkind & Kostenis, 2018; Kenakin, 2019). However, the molecular mechanisms underlying these complex and variable interactions remain far from fully understood (Smith et al., 2021; Thomsen et al., 2016).

‘Biased signalling’ is the ligand-dependent activation of certain pathways over others, and can lead to a ‘functionally selective’ response. ‘Biased signalling’ became generally accepted after evidence accumulated that the rank order of ligands by potency could be different for different pathways engaged by a single receptor (Roth & Chuang, 1987; Spengler et al., 1993) or inversion of the ligand modality (Azzi et al., 2003; Baker, Hall, & Hill, 2003). The most frequently studied pathway-bias has been that between G proteins and arrestins, while more recent studies have compared G protein families and even subtypes belonging to the same G protein family. Several receptors have evolved to recognize multiple alternative endogenous agonists eliciting biased signalling (relative to the principal endogenous ligand), for example, chemokine (Kohout et al., 2004), opioid (Gomes et al., 2020), PACAP (Spengler et al., 1993), protease-activated (Hollenberg et al., 2014), 5-HT (serotonin) (Schmid, Raehal, & Bohn, 2008), and PTH (Dean, Vilardaga, Potts, & Gardella, 2008) receptors. Therapeutic exploitation of biased signalling could increase drug efficacy while avoiding adverse effects attributable to particular pathways. Several studies have outlined disease-relevant pathways for future therapeutic targeting (Urban et al., 2007; Whalen, Rajagopal, & Lefkowitz, 2011) or retrospective cross-screening yielding biased ligands predicted to result in potentially useful phenotypes in therapy (Che, Dwivedi-Agnihotri, Shukla, & Roth, 2021; Galandrin, Oligny-Longpre, & Bouvier, 2007; Kenakin, 2019; Urban et al., 2007; Whalen, Rajagopal, & Lefkowitz, 2011).

Mechanistically, biased signalling has been explained by conformational selection. Specifically, a ligand and transducer—binding from opposite sides of the cell membrane—may prefer the same receptor structure conformation and thereby allosterically select each other (Galandrin, Oligny-Longpre, & Bouvier, 2007; Kenakin, 1995; Kenakin & Morgan, 1989; Roth & Chuang, 1987; Smith, Lefkowitz, & Rajagopal, 2018). In other words, this allosteric communication is reciprocal, as G protein pre-coupling can potentiate agonist binding (Bock & Bermudez, 2021; De Lean, Stadel, & Lefkowitz, 1980; Lefkowitz, Mullikin, & Caron, 1976; Maguire, Van Arsdale, & Gliman, 1976). An activated receptor state has also been linked to a high affinity binding state for arrestin (Gurevich & Benovic, 1997). However, it is still unclear what the precise relationship between conformation and signalling is—at least at the level of detail required to predict such outcomes.

Biased signalling comprises very complex pharmacology making experiment design, interpretation and description challenging and often inconsistent—causing confusion about what has really been measured and what can be concluded. Here, we provide recommendations on ligand bias intended to serve as common guidelines for the field along with defined terminology. They are supported by the authoritative organization for pharmacological nomenclature, the Nomenclature and Standards Committee of the International Union of Basic and Clinical Pharmacology (NC-IUPHAR, https://www.guidetopharmacology.org/nciuphar.jsp), and COST Action CA18133 ERNEST (European Research NEtwork on Signal Transduction) (Sommer et al., 2020). Each section below provides definitions, terminology and recommendations for experiments and reporting (summarized in Data S1, S2, and S3).

## 2 Definitions of Pathways and of Bias Types

### 2.1 Pathway definition and modulation

A GPCR pathway is here defined by a transducer protein, or family thereof, binding intracellularly to the receptor and eliciting a distinct cellular downstream signalling cascade, trafficking or internalization. Based on present knowledge, this includes the four G protein families—that is, the G_s_, G_i/o_, G_q/11_, G_12/13_ pathways—and the two GPCR kinase (GRK) and arrestin families (in all six transducer protein families). Typically, there is a sequential engagement of a receptor with a G protein, GRK, and arrestin. However, GRKs and arrestins can be recruited to and bind to GPCRs independently of functionally active G proteins (Grundmann et al., 2018; Hunton et al., 2005; Sauliere et al., 2012; Wehbi et al., 2013). For example, GRK4-6 functions do not appear to require either G proteins or arrestins, as they are not recruited by G_βγ_ but anchored to the plasma membrane via polybasic domains and lipid modification (Komolov & Benovic, 2018). Ligand bias is not limited to the comparison of these six transducer protein families but can also occur within each such family, that is, across its different members. This extends the number of pathways to 27: 16 Gα proteins, 7 GRKs, and 4 arrestin proteins. For example, G proteins belonging to the same family may differ in their functional outcome due to unique binding kinetics, cellular expression levels, and engagement of different downstream effectors (Anderson et al., 2020; Avet et al., 2021; Ho & Wong, 2001; Jiang & Bajpayee, 2009; Olsen et al., 2020). Similarly, differential recruitment of the two isoforms of β-arrestin (β-arrestin 1–2) can translate to distinct functional outcomes, with respect to regulatory and signalling paradigms (Ghosh et al., 2019; Srivastava, Gupta, Gupta, & Shukla, 2015).

In addition, a range of modulatory proteins interact with and change the signalling of receptors, transducers or effectors. For example, receptor activity-modulating proteins (RAMPs) bind to receptors and can alter their trafficking as well as G protein and/or arrestin binding (Hay & Pioszak, 2016). In the case of the calcitonin and calcitonin receptor-like receptor, different receptor-RAMP complexes produce distinct pharmacological responses and are therefore considered as separate receptor subtypes: one calcitonin, two adrenomedullin, and three amylin receptors (Hay, Garelja, Poyner, & Walker, 2018). Similarly, the cannabinoid CB_1_ receptor can bind to cannabinoid receptor interacting protein 1a (CRIP1a), yielding distinct pharmacology (Oliver et al., 2020). GPCRs are also substrates for second messenger-activated kinases such as the cAMP-dependent kinase (PKA), PKC, and the casein kinase (CK), with each producing different effects on receptor signalling and trafficking (Bouvier, Leeb-Lundberg, Benovic, Caron, & Lefkowitz, 1987; Hausdorff et al., 1989; Tobin, Totty, Sterlin, & Nahorski, 1997). Additionally, numerous downstream intracellular effectors modulate pathway responses as scaffolding proteins, for example, kinases and PDZ proteins (Bockaert, Fagni, Dumuis, & Marin, 2004; Kenakin, 2019; Maurice et al., 2011). The regulator of G protein signalling (RGS) proteins selectively modulate G protein subtypes and differentially alter G protein signal strength (Hollinger & Hepler, 2002; Masuho et al., 2020; Neubig & Siderovski, 2002). Furthermore, GRK2 and GRK3 have a RGS homology domain (RH) binding to G_q/11_ to inhibit signalling, and a pleckstrin homology (PH) domain that can bind to G_βγ_ to inhibit its signalling while inducing recruitment of GRK to the receptors (Carman et al., 1999; DebBurman, Ptasienski, Benovic, & Hosey, 1996; Ribas et al., 2007).

**Terminology summary A**

***Transducer:*** For the purpose of defining biased signalling initiated by the GPCRs, transducers are defined as proteins that bind directly to an activated receptor to initiate, facilitate or modulate downstream events such as signalling, trafficking or internalization. This includes G proteins, GRKs and arrestins. Some also use ‘primary’ effector to denote a transducer, but this word can be confusing as these proteins typically bind the receptor one after the other.***Effector:*** Signalling protein located downstream in a transducer’s pathway. ***Modulator:*** Proteins or molecules that do not mediate, but modulate signalling of a receptor, transducer or effectors. Examples include receptor activity-modulating proteins (RAMPs), guanine nucleotide exchange factors (GEFs), GTPase-activating proteins (GAPs), regulators of G protein signalling (RGSs), NO, cholesterol and other lipids. ***Second messenger:*** Small molecules or ions directly controlled by the effectors. Changes in second messenger homeostasis mediate cellular responses and can serve as a quantifiable measure of GPCR activation. Examples include cAMP and calcium. ***Pathway:*** A pathway is named after a transducer protein, or family thereof, that binds to GPCRs. This includes G proteins and their families—that is, G_s_, G_i/o_, G_q/11_, and G_12/13_ which have distinct downstream effectors. It also includes the GPCR kinase (GRK) and arrestin families, which are often but not always engaged following G protein activation.

### 2.2 Ligand bias definition and distinction from system bias

**Figure 1:**
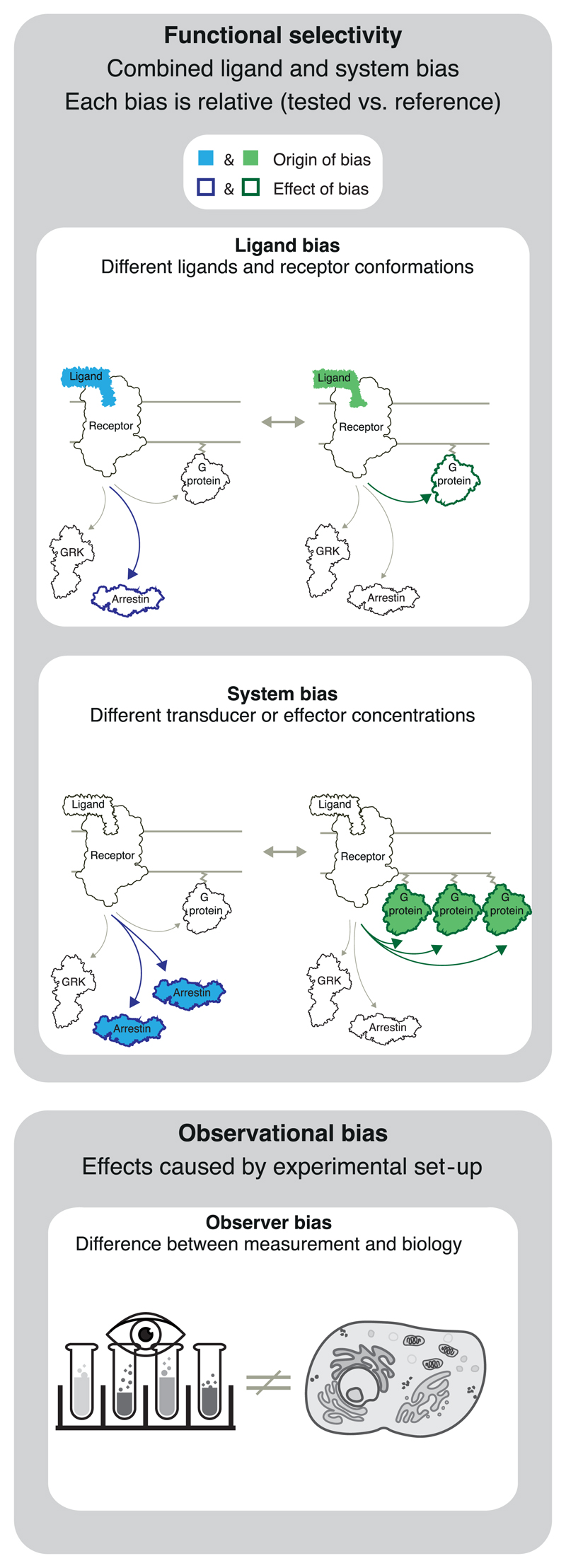
Ligand, system and observational bias. Ligand and system bias together determine the functional selectivity (see terminology subsection for definitions). Each type of bias is measured relative to a reference. Observational bias is not of biological origin, but a consequence of assay sensitivity/non-linearity and the experimental set-up

This paper focuses on **ligand-dependent bias,** that is, cases where a receptor’s pathway engagement changes when binding a given ligand (Figure 1). Quantification of bias compares two pathways at a time, typically the pathway with the strongest signalling relative to the most relevant other pathway(s). It also uses a reference ligand and is therefore a comparison of both pathways and ligands (like a quantitative rank order).

In addition to ligand bias, a receptor’s response is also determined by **system bias,** which encompasses all non-ligand molecules involved in signalling. System bias encompasses, for example, the stoichiometry of receptors, transducers, effectors, and modulatory proteins which varies spatially across tissues, cell types, and temporally across physiological states. System bias includes a receptor’s G protein or arrestin selectivity. For example, some GPCRs lack the inherent ability to elicit G protein coupling while exhibiting robust arrestin interaction (Meyrath et al., 2020; Pandey et al., 2021; Rajagopal et al., 2010). Furthermore, system bias depends on the different levels of receptor constitutive activity, and intracellular intra- or inter-pathway feedbacks. System bias affects all ligands equally (including the reference ligand), unless the system itself has an influence on ligand concentration (e.g. by conditions where one of two ligands is metabolized).

**Functional selectivity** is the combined effect of ligand and system bias (Stallaert, Christopoulos, & Bouvier, 2011). For example, a GPCR can give rise to different physiological effects after activation by alternative endogenous agonists or for the same ligand in different cells/ tissues with differentially expressed signalling components. In drug discovery, achieving functional selectivity by tuning ligand bias on the background of system bias in the tissue of interest provides an opportunity to elicit predominantly the therapeutically beneficial effect of a receptor target.

Biased signalling studies can also suffer from **observational bias,** which is an artificial bias caused by an inappropriate experimental set-up and therefore not reflecting native biology. An example are time points for data collection that are not relevant for the physiological effect. Another example is if one of the two studied pathways is measured using an assay with insufficient sensitivity or, conversely, has an artificially high level of amplification. Therefore, it is necessary to ensure that at least one assay for each pathway has sufficient sensitivity (preferred) or to increase expression levels of the involved signalling partners (alternative). Moreover, the actual signal plateau may be missed if the signal detection tools saturate prematurely. Observational bias can be avoided by using an independent ‘orthogonal’ assay to validate each pathway.

**Disclaimer:** A ligand may be unbiased (relative to the reference ligand) with respect to two particular pathways but may be biased when additional different pathways are studied or if a different reference ligand is used. Hence, the term ‘biased ligand’ cannot be used as an absolute descriptor for a ligand, but only in the context of receptor, reference ligand, and pathways.

**Terminology summary B**

***Ligand bias:*** Ligand-dependent preferential receptor activation so that one over other transducer pathways in a given cellular system and relative to a reference ligand is induced (see below).

***System bias:*** Bias due to differences in the cellular system, including so called ‘tissue bias’. System bias can, for example, arise from differential expression levels of receptors, transducers, effectors, or modulatory proteins.

***Functional selectivity:*** Functional selectivity is the observed response combining ligand- and system-bias.

***Observational bias:*** An artificial bias introduced by the experimental set-up. This effect can be minimized, for example, by using equally sensitive assays across pathways.

## 3 Choosing a reference Ligand

### 3.1 The choice of reference ligand distinguishes benchmark-, pathway-, or physiology-bias

The meaning of ligand bias depends on the choice of reference ligand (Table 1). When the reference ligand is a tool compound, for example, isoprotenerol/isoprenaline for the β_2_-adrenoceptor) or a drug (e.g. to investigate if an agent in a clinical trial is different from this drug), we refer to this as **ligand benchmark-bias,** as all that can be concluded is how the ligands differ in bias. In contrast, **ligand pathway-bias** utilizes the reference ligand with the most balanced signalling across pathways, allowing the researcher to identify ligands for or functionally dissect a specific pathway. Finally, **ligand physiology-bias** utilizes the principal physiological agonist as the reference ligand and consequently can conclude whether the tested ligands induce ‘natural’ or ‘unnatural’ signalling. Thus, the three types of ligand bias are complementary in that they allow different scientific conclusions to be drawn for the tested ligands.

As an example, the choice of reference ligand changes the preferred pathway of the κ-ĸ-opioid receptor agonist naphthoyl-β-naltrexamine (β-NNTA) (White et al., 2014). Using the endogenous ligand dynorphin-A as the reference, β-NNTA has a 4.3-fold bias for β-arrestin recruitment over G protein activation. However, when using the plant psychedelic salvinorin A, β-NNTA displays a 8.9-fold G protein bias instead. When using an analogue, salvinorin B, β-NNTA still has a G protein bias but less pronounced, only 2-fold.

#### 3.1.1 Problems and pitfalls

- Bias cannot be reported without the use of a reference ligand, as a presumed bias may otherwise in fact be due to other differences in, for example, the baseline, window, and sensitivity of assays (‘system bias’ or ‘observational bias’ in Figure 1).
- It cannot be concluded that a tested ligand induces isolated/ specific signalling via one pathway unless a balanced ligand is used as reference. In all other cases, the only conclusion is that the tested ligand differs from the reference. This is because the reference ligand may itself have pathway-bias, making a tested balanced ligand appear biased in comparison.

#### 3.1.2 Recommendations for experimental design

- Choose a reference ligand that can support the claims to be made (Table 1). Use a pathway-balanced and physiological reference ligand to study pathway- and physiology bias, respectively.
- Include multiple reference ligands, thus allowing claims about different types of ligand bias. Doing this will extend the insights from the current study and enable comparison to other studies. This entails, if possible, the inclusion of all types of reference ligand: a highly used tool compound, a drug, a pathway-balanced ligand, and a physiological ligand. Such ligands are available from the receptor pages of the Guide to Pharmacology database (Harding et al., 2021) and the ligand and drug resources in GPCRdb (Hauser, Attwood, Rask-Andersen, Schioth, & Gloriam, 2017; Kooistra et al., 2021).
- Measure the reference ligand(s) and the ligands tested for bias, under identical conditions.
- If needed, use separate reference ligands for bias and E_max_. If the reference ligand for bias is not a full agonist, but a partial agonist producing less than a full receptor response (maximum efficacy, E_max_), a separate reference ligand for E_max_ should be used to exploit the full window of receptor response. For the G_s_ pathway, some studies use forskolin, which activates adenylate cyclase directly, rather than a reference ligand to determine the maximal response. It is possible to use different reference ligands for E_max_ in the different pathways, as long as the reference ligand for bias and all ligands tested for bias are normalized relative to the same reference ligand for E_max_. For mathematical models quantifying bias, see Section 6.2.

#### 3.1.3 Reporting recommendations

- Report the reference ligand along with a motivation to why it was chosen. For example, ‘The reference ligand A was chosen, as it (i) is the principal physiological agonist, (ii) has a relatively balanced signalling through pathways P1 and P2 (provide bias plot slope, or (iii) a clinical or tool compound for which enhanced pathway specificity is desired through the introduction of bias’.
- The claims should be restricted to what is supported by the chosen reference ligand (Table 1). Thus, in order to conclude on pathway- and physiology-bias, a balanced and physiological reference ligand, respectively, must be used.
- Define the bias type. Instead of just ‘ligand bias’, we recommend using the specific terms ‘ligand benchmark-bias’, ‘ligand pathway-bias’, and ‘ligand physiology-bias’ when the reference ligand is a drug/probe, balanced and physiological ligand, respectively. Although the person who generated the data may only be interested in one type of bias, the community understands the meaning of bias differently.

#### 3.1.4 Disclaimer

**Assay/system dependence:** If the system or assays change, the same ligands, receptor, and pathways can display a different bias. This is because a reference ligand does not automatically equalise an imbalance in pathways but merely moves the baseline. Imbalance in pathways is instead predominantly determined by the choice of systems and assays, which should be as similar as possible.

**Terminology summary C**

**Biased ligand:** Ligand preferentially activating one receptor transducer pathway in a given cellular system and relative to a reference ligand. Ligand bias is a property of not just a ligand, but of a ligand, pathway pair and receptor in combination, and only valid within the specific system that is investigated. Therefore, the term ‘biased ligand’ should only be used if explicitly defined, and not be construed to represent a ligand-only property. A recommended definition is included in Section 8.1, which provides one-sentence and table templates for reporting.

**Reference ligand for bias:** The ligand that is, by definition, unbiased. The bias of any other tested ligands is quantified relative to this reference.

**Reference ligand for E_max_:** A separate reference ligand for the full receptor response (maximum efficacy, E_max_). This allows exploiting the full window of receptor response even when the reference ligand for bias is a weak partial agonist.

**Unbiased ligand:** A ligand that stimulates pathways in a manner indistinguishable from the reference ligand.

### 3.2 Ligand pathway-bias (uses a pathway-balanced reference ligand)

Ligand pathway-bias is the most relevant type of ligand bias when seeking to discover a drug with functional selectivity, as it describes predominant signalling via one pathway. A pathway-balanced reference ligand (typically a surrogate, but can also be an endogenous ligand) has near equal signalling through compared pathways, meaning that if a tested ligand is biased, it preferentially activates one pathway over the other (Table 1).

#### 3.2.1 Problems and pitfalls

**Figure 2:**
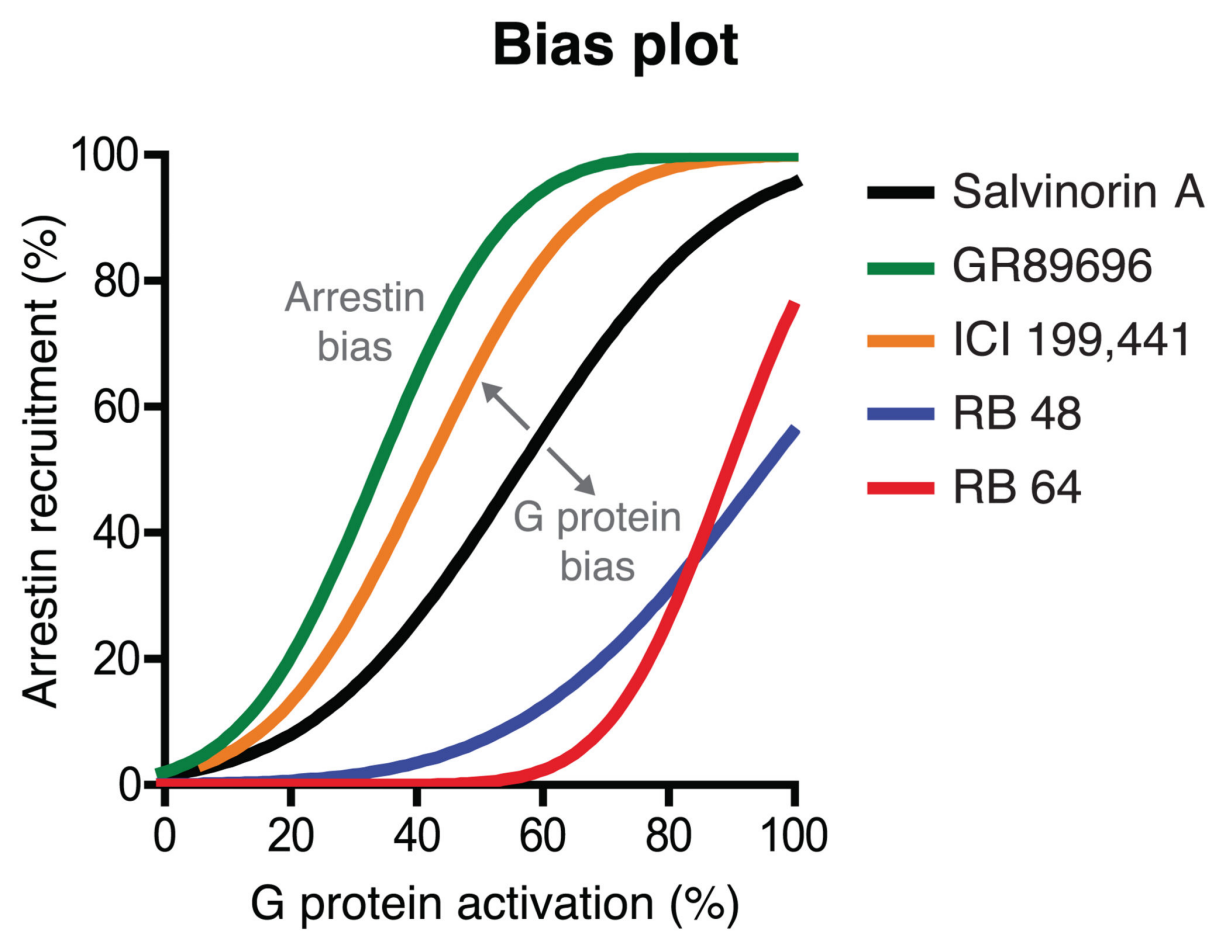
Bias plot of an equimolar comparison of ligand-induced activities in two pathways. The plot is adapted from White et al., (2014) in which Salvinorin A was chosen as the reference ligand because it has a bias plot slope close to 1. GR89696 and ICI 199,441 are arrestin-biased and RB 48 and RB 64 are G protein-biased. The pathway percent activation could, for example, use ΔΔLog (E_max_/EC_50_) or (ΔΔLog(τ/K_A_) values (see Section 6.2 and Box 1)

**Lacking reference ligands:** When no balanced ligand is available, bias can still be measured but not interpreted as pathway-bias. In these cases, it can be useful to visualize the relative pathway preferences of the most interesting tested ligands in a bias plot (Figure 2).

#### 3.2.2 Recommendations for experimental design

- Determine a pathway-balanced reference ligand in a bias plot. A balanced ligand can be defined based on a bias plot, which is an equimolar comparison of induced pathway activities (Figure 2). A balanced (least biased) ligand has a slope of (close to) 1.

#### 3.2.3 Reporting recommendations

- Document the pathway-balance of the reference ligand. For example, present a bias plot (Figure 2) or reference a previous supporting study, if available.

#### 3.2.4 Disclaimer

**Context specific bias:** Many researchers argue, rightly so, that a ligand referred to as balanced will not be balanced in another system or assay. Therefore, any claim of a ligand being balanced and tested ligands exhibiting pathway-bias is always system-specific (which is true for all ligand bias studies). See also the Section 5.1.

**Terminology summary D**

**Balanced ligand:** Has indistinguishable or very similar signalling through compared pathways (Figure 2).

**Ligand pathway-bias:** Ligand bias that is measured relative to a balanced reference ligand and therefore has the meaning that signalling is predominant via one pathway.

### 3.3 Ligand physiology-bias (uses the principal endogenous ligand as reference)

When using an endogenous ligand as a reference ligand, the statement that a tested ligand is biased carries the meaning that its signalling differs from the physiological one (Table 1). This is valuable, for example, when seeking to map the relationships between pathways and physiological effects. Whether this entails a response through a single pathway or not will therefore depend on the extent to which the endogenous ligand engages different transducer pathways or not, in the first place.

#### 3.3.1 Problems and pitfalls

- The same receptor often has multiple endogenous agonists and depending on which one is used as the reference for a ligand physiology-bias study, the measured bias may change substantially.
- A secondary endogenous agonist can be biased relative to the principal endogenous agonist.
- Ligand physiology-bias cannot be determined for ‘orphan’ GPCRs, as they have no known endogenous ligand.
- Endogenous ligands with low efficacy cannot be quantified for bias (see Section 7.1).

#### 3.3.2 Recommendations for experimental design

- Use the principal endogenous agonist as reference ligand for studies of how a tested ligand differs from the natural signalling, that is, ligand physiology-bias. The principal endogenous agonist is that which is most abundant and potent in the receptor‘s most physiologically relevant tissue. Principal and secondary endogenous agonists of GPCRs are curated by experts coordinated by NC-IUPHAR and available in the receptor pages of the Guide to Pharmacology database (Harding et al., 2021). In cases where it is not clear which endogenous agonist represents the principal agonist, we recommend using the endogenous agonist with the highest potency (efficacy is not annotated in this database) or efficacy in the given investigators’ assay and system.

**Terminology summary E**

**G protein selectivity:** The profile of one or more G proteins that a receptor engages, typically when activated by the principal endogenous ligand reference. This may be a subset of additional G proteins that the receptor can engage when bound to other agonists. The term ‘natural bias’ is self-contradictory and should not be used.

**Ligand physiology-bias:** Ligand bias relative to a receptor’s principal endogenous agonist, which therefore bears the meaning that signalling differs from the physiological.

### 3.4 Ligand pathway-preference (uses no reference ligand, and is not ligand bias)

Without a reference ligand, a study cannot conclude on ligand bias. Investigation of a ligand’s differential activity across pathways (e.g. pathway ΔLog (E_max_/EC_50_) or ΔLog(τ/K_A_) values), but not relative to a reference ligand, is here instead referred to as **pathway-preference.** Comparing pathway-preferences of a number of tested ligands can be a useful way to select a reference ligand for subsequent calculation of ligand bias. It may also be a way to rank order ligands without defining a fixed reference point.

#### 3.4.1 Problems and pitfalls

- Studies cannot claim ligand bias without a reference ligand.

#### 3.4.2 Recommendations for experimental design

- Compare pathway ΔLog (E_max_/EC_50_) or ΔLog(τ/K_A_) values, not only fold potencies, as differing efficacies can substantially influence the relationships (Box 1).
- Use the same or near-identical systems and assays, as without a reference ligand any system bias will have a larger influence.

**Terminology summary F**

**Pathway-preference:** A ligand’s differential activity across pathways (e.g. pathway ΔLog (E_max_/EC_50_) values), but without comparison to a reference ligand.

## 4 Measuring at the Transducer or Downstream

### 4.1 Ligand bias measured at the transducer level

Measuring receptor-binding transducers (G proteins, GRKs, or arrestins) avoids downstream signal amplification. This can make experiments more comparable across pathways than measuring downstream effectors or second messengers.

#### 4.1.1 Problems and pitfalls

**Figure 3:**
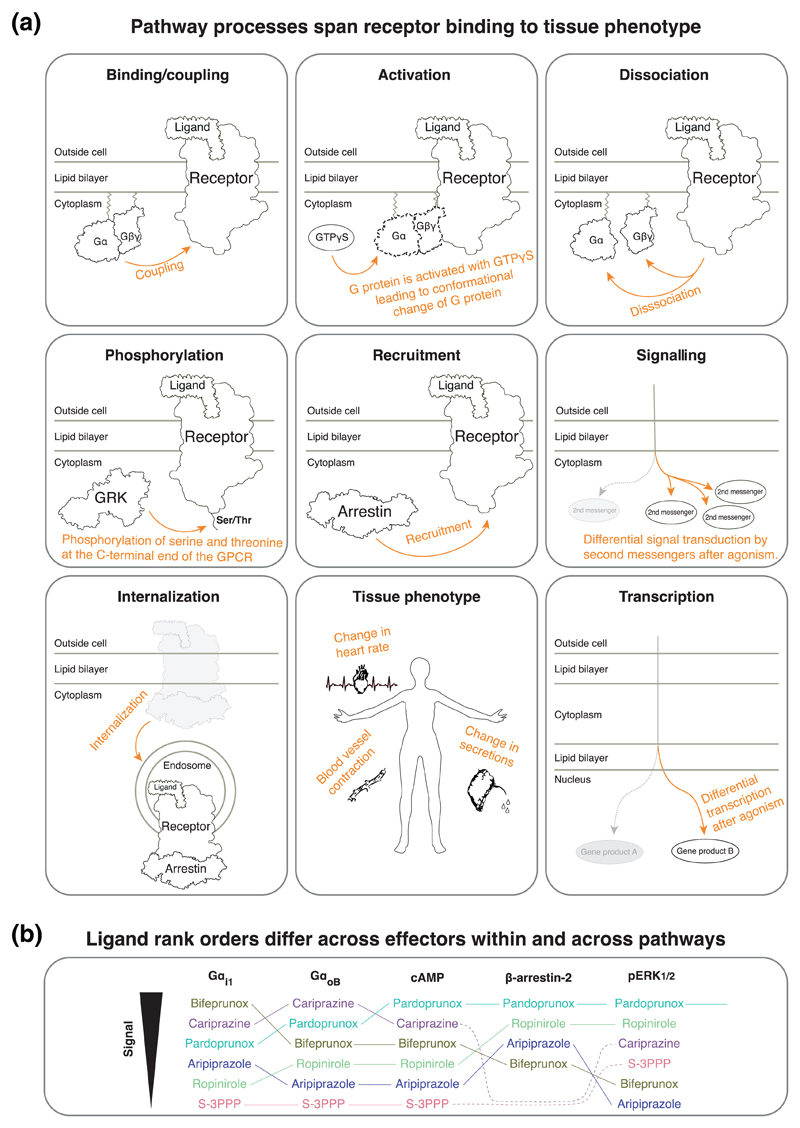
Recommendations to describe the measured pathway process and molecules. (a) Terms to describe the measured pathway process. Also see reviews describing the translation across these levels (Luttrell, Maudsley, & Gesty-Palmer, 2018), assays (Smith, Lefkowitz, & Rajagopal, 2018) and animal models (Bradley & Tobin, 2016). (b) Data from (Klein Herenbrink et al., 2016) show the relative bias of dopamine D_2_receptor agonists for five pathways (all with dopamine as the reference ligand). The relative order of the ligands changes depending on the measured molecules, even across those participating in the same pathway. This emphasizes that bias should be measured at similar pathway depths (comparably proximal/distal to the receptor), each of which should be defined with respect to the measured entities

- If a single representative transducer is used, it may not be representative for the signalling behaviour of the entire transducer family (e.g. G_q_, G_11_, G_14_, and G_15_ in the G_q/11_ family). Differential activation or recruitment of individual transducer family members has been shown both for G protein families (Avet et al., 2021; Inoue et al., 2019; Masuho et al., 2015; Namkung et al., 2018; Olsen et al., 2020) and the arrestin family (Avet et al., 2021; Srivastava, Gupta, Gupta, & Shukla, 2015).
- Different cellular systems can express transducers differentially. Hence, even measurements of transducer-levels, albeit devoid of signalling amplification, can differ across cells and tissues.
- Assays are often only referred to by their detection method, for example, ‘BRET assay’. This is insufficient, as the same detection technique can be used to measure fundamentally different molecules and processes, such as GPCR-G protein binding/coupling or G_α_-G_βγ_ dissociation (Figure 3a).

#### 4.1.2 Recommendations for experimental design

- Use recent assays consistently profiling G proteins and arrestins (also with GRKs) (Avet et al., 2021; Olsen et al., 2020) (The assays in Olsen et al. were optimized from Gales et al. 2006; Lukasheva etal., 2020).
- If possible, test all transducers for each investigated transducer family because it cannot be assumed that one transducer is representative for the signal mediated by the entire family.
- If selecting representative transducer subtypes, use the most relevant. If not all transducer subtypes within a given transducer family can be tested, a representative could be selected based on the transducer expression levels in the tissue most relevant for the study. If this is not known, another rationale could be to select the transducer subtype with the highest activation or recruitment. Data to support such a selection have come from, for example, Avet et al. (2021), Inoue et al. (2019) and Olsen et al. (2020) and have been integrated in GproteinDb (Pandy-Szekeres et al., 2022) and ArrestinDb (https://arrestindb.org).

#### 4.1.3 Reporting recommendations

- Modifications of receptors, transducers, or effectors must be clearly defined (e.g. tags, mutations and chimeras).

### 4.2 Ligand bias measured downstream of distinct transducers

Whereas ligand bias is often grouped into pathways represented by the primary receptor-binding transducers (G proteins, GRKs, and arrestins), most studies measure downstream effector proteins or second messenger molecules (Figure 3a). Unlike measurements at the transducer level (previous section), this collates the signalling of all subtypes of a transducer family and may correlate better to the tissue-level or systemic response. However, they may suffer from pathway convergence or crosstalk (see Section 4.3).

#### 4.2.1 Problems and pitfalls

- If two pathways are measured at different depths, one close to the receptor and another further downstream, this can lead to a higher signal amplification for the latter pathway. This observation bias can cause apparent ligand bias. For example, arrestin is nearly always measured as recruitment or binding to receptors because there is no consensus on a downstream effector or second messenger. Conversely, G protein pathways are often measured at the downstream level, typically as a secondary messenger.
- Ligand bias relationships may differ depending on which of a pathway’s downstream molecules are measured (Figure 3b).

#### 4.2.2 Recommendations for experimental design

- Minimize differential signal amplification by measuring pathways at similar depth (estimated based on the number of upstream effectors, Figure 3a). Where the pathway readouts differ in depth, special consideration should be given to potential amplification effects.

#### 4.2.3 Reporting recommendations

- Report measured processes. The same molecule can be involved in several steps of the signalling process. For example, G proteins can be measured for receptor binding, activation, nucleotide exchange, or subunit dissociation. A non-exhaustive list of terms to distinguish such signalling processes is visualized in Figure 3a and tabulated in Table 2 along with example assay principles.
- Report the measured molecules. To provide clarity on what has been measured, we recommend that the pathways are defined not only for the upstream GPCR-binding transducer but also for the downstream measured molecule or molecule pair (Table 3).

### 4.3 Ligand bias measured downstream of converged transducer pathways

#### 4.3.1 Problems and pitfalls

- Several transducer pathways intercept or converge downstream. If measuring at or downstream of such pathway nodes, the signal cannot be mapped back to a specific transducer. For example, ERK proteins can be activated by all four G protein families (Jain, Watson, Vasudevan, & Saini, 2018), and this process is shaped in space and time by arrestins and GRKs (Eichel, Jullie, & von Zastrow, 2016; Gurevich & Gurevich, 2018; Gutkind & Kostenis, 2018; Luttrell et al., 2018; Wehbi et al., 2013).

#### 4.3.2 Recommendations for experimental design

- Avoid measuring downstream of converged pathways, unless the purpose is to compare two ‘deep’ signals close to the cellular end-points without a need for relative transducer contributions.
- If possible, dissect upstream transducer contributions using other assays.

#### 4.3.3 Reporting recommendations

- When pathways are truly inseparable and their contributions cannot be dissected using upstream assays, the bias may be described as a type of ‘effector bias’ (instead of pathway-bias) accounting for the net pathway contributions.
- Interpret findings in light of their relative strength for the given receptor and ligand. For example, calcium, PKC, or DAG measurements should not be exclusively equated to G_q_ activation. Indeed, different receptor transducers, including G_βγ_ released from G_αi_ can also lead to the generation of these second messengers and activation of this kinase (Dorn, Oswald, McCluskey, Kuhel, & Liggett, 1997).

## 5 Considering Cellular System, Kinetics and Spatial Bias

### 5.1 Bias may not translate across in vitro, physiological, and therapeutic systems

#### 5.1.1 Problems and pitfalls

- Most in vitro pharmacology experiments are performed in systems (e.g. HEK cells) that may differ substantially from a physiologically or therapeutically relevant system. Given the cell phenotype- and physiological cell state-dependence of bias, the measured bias incurs the risk of not translating to the expected physiological or therapeutic effect in human (Bohn, Zhou, & Ho, 2015).
- The use of recombinant and/or overexpressed receptor, transducer, or effector proteins may not reflect the ligand bias in a native system. Insofar as ligand bias quantification can be carried out in a recombinant system, it serves to identify bias as a property of the molecule (not the system) and still serves as an identifier for molecular mechanisms to be elucidated further in natural systems.
- Some signalling pathways are difficult to evaluate in tissues (i.e. arrestin recruitment) as many assays require genetically modified tags to be applied to receptors and/or effectors. If modifications are made to proteins in endogenous systems, those modifications must be clearly indicated, and interpretations should consider the possible effects of overexpression and modification on the system.

#### 5.1.2 Recommendations for experimental design

- Where possible, use primary and/or disease-specific cells and evaluate potential system bias.
- Where possible, validate the effect in a model organism using an appropriate model of efficacy and/or genetic engineering to confirm target and pathway specificity.

#### 5.1.3 Reporting recommendations

- Where a non-native system has been used, conclusions should be stated carefully such that a ligand inducing bias in this system may or may not have a beneficial effect in a therapeutically relevant system in vivo.

### 5.2 Kinetics and choosing measurement time points

#### 5.2.1 Problems and pitfalls

- Ligand bias often depends on the time points of data collection. For instance, comparing non-equilibrium readings with equilibrium readings due to different binding kinetics or type of biological responses (ion flux vs. reporter gene) can be a major confounding factor (Klein Herenbrink et al., 2016).
- The physiologically most relevant time point is often the same across pathways but can differ if the signalling processes (Figure 3a) occur over different time scales.
- A ligand’s effect is time-dependent, as signalling efficacy changes (e.g. due to desensitization), which can be pathway-dependent (Stout, Clarke, & Berg, 2002).

#### 5.2.2 Recommendations for experimental design

- When possible, complete time courses and endpoint measurements should be made. These could be quantified by onset kinetics, for example, time constant tau (τ) or time to reach half maximal response amplitude (Hoare, Pierre, Moya, & Larson, 2018).
- Single time points should be the physiologically most relevant or measure the maximum effect (peak).

#### 5.2.3 Reporting recommendations

- Report the chosen time points and the reason(s) why they were chosen.
- Provide data on the complete time course, if available.

**Further reading:** See Lane, May, Parton, Sexton, & Christopoulos (2017).

**Terminology summary G**

***Temporal effect:*** The effect influencing a measured response due to the choice of time point at which a response is recorded. Apparent bias can occur simply because the kinetics of the response is different between two pathways for two different ligands. These time effects include effects of the binding kinetics (k_on_ and k_off_), time course of the biological response measured, and time domain of the assay itself (e.g. reporter gene vs. ion flux).

### 5.3 Spatial bias: Differing signalling efficacies across cellular compartments

#### 5.3.1 Problems and pitfalls

GPCRs signal from a wide range of cellular compartments other than the plasma membrane, including endosomes, the Golgi and the nucleus (Crilly & Puthenveedu, 2021; Jong, Harmon, & O’Malley, 2019). This signalling can be modulated by different transducers, such as β-arrestins (Luttrell et al., 2001), G_α_ subunits (Feinstein et al., 2011; Irannejad et al., 2017), and G_βγ_ subunits (Masuho, Skamangas, Muntean, & Martemyanov, 2021). With this has come the realization that signalling involving the same transducer from different compartments can have distinct outcomes; for example, cAMP evolution from endosomes but not the plasma membrane promotes gene transcription (Tsvetanova & von Zastrow, 2014). This phenomenon of GPCR signalling through the same transducer in different locations producing distinct signalling responses has been referred to as ‘spatial’ or ‘location bias’. Furthermore, spatial bias extends to ligand-biased trafficking. For example, ligands differentially regulate receptor recycling following endocytosis (Namkung et al., 2016).

#### 5.3.2 Reporting recommendations

- Report the biosensors and tags used for monitoring compartment-specific signalling.
- Report the cell types used in assays, as some cell types lack transporters, such as OCT3/SLC22A3, required for the trafficking of hydrophilic small-molecule ligands that cannot cross the plasma membrane (Irannejad et al., 2017).
- Report ligands with altered characteristics, for example, permeability, through chemical modification (Jensen et al., 2017).

**Terminology summary H**

***Spatial/location bias:*** The observation of biased GPCR signalling through the same transducer in different locations that results in distinct signalling responses. This GPCR signal may originate from different compartments or may be from the same compartment but then result in the trafficking of transducers to different compartments (Masuho, Skamangas, Muntean, & Martemyanov, 2021).

## 6 How to Quantify Ligand Bias

### 6.1 Many models exist to quantify ligand bias

The classical theory of receptor action (Furchott, 1966; Stephenson, 1956) aims to separate ligand- and system-dependent parameters and forms a basis of strategies to quantify ligand bias. Many methods to quantify ligand bias are based on the null principle of equi-effective concentrations of agonist producing equal responses, first described by Barlow, Scott, and Stephenson (1967) and theoretically justified within the framework of the Black-Leff operational model (Black & Leff, 1983).

However, there are still different views on how to best quantify bias and strategies for quantification are still being developed and improved. Hence, there are numerous models that could be used, as long as pathways are measured with consistent pharmacological parameters, and in similar systems and assays with corresponding levels of downstream processes and kinetics (see above). Of note, even with the most detailed models, it is not possible to directly compare quantitative estimates of ligand bias across different studies.

#### 6.1.1 Problems and pitfalls

- Quantification of ligand bias with different methods can in some cases lead to different conclusions on the biased/unbiased nature of a ligand (and system) (Onaran et al., 2017; Rajagopal et al., 2011) or to a different relative bias rank order of ligands.
- No model can provide an absolute bias value of a given ligand at a given receptor. Only bias values relative to a reference ligand are accessible with current quantification techniques.

#### 6.1.2 Recommendations for experimental design

- It is not possible to recommend a single, best-practice, quantification method due to the lack of consensus in the community, with new or refined methods still being proposed.
- Results are more definitive when bias is quantified using multiple models.
- Irrespective of the model, error propagation and statistics must be handled appropriately.

**Further reading:** We may refer readers to Table 4 in Kenakin (2019) for an overview of methods to quantify ligand bias, and Onaran and Costa (2021) for a critical review of the detailed principles, on which specific implementations are based.

### 6.2 A minimal and a refined model to calculate a ligand bias factor

The two most commonly used models to quantify ligand bias are relative-relative Log (E_max_/EC_50_) (Ehlert, 2008) and relative-relative Log(τ/K_A_) (Box 1, Kenakin, 2015b). ΔΔLog (E_max_/EC_50_) is easy to use, as it only requires E_max_ and EC_50_ values for which the log ratio is first compared for two ligands and subsequently for two pathways (Box 1). For ligands with concentration–response curve slope factors (Hill coefficients) of 1, the ΔΔLog (E_max_/EC_50_) values are identical to Log (τ/K_A_) values. For other ligands, ΔΔLog(τ/K_A_) values are more correct, as they account for receptor density and coupling within a system. Like E_max_/EC_50_, τ/K_A_ can be calculated from a concentration–response curve, but it requires a software, such as GraphPad Prism, to solve the Black/Leff model and special care for full agonists which can have ambiguous fits (Box 1). Both ΔΔLog (E_max_/EC_50_) and ΔΔLog (τ/K_A_) can be expressed as a bias factor, which is the antilog. As the bias factor is on a logarithmic scale, a difference of 1 corresponds to a 10-fold activation difference. A template (MS Excel file) to calculate bias factors is available at https://files.gpcrdb.org/bias_guidelines/Template_to_calculate_ligand_bias_factors.xlsx.

#### 6.2.1 Recommendations for experimental design

- ΔΔLog (E_max_/EC_50_) should not be used when ligand concentration–response curve slope factors (Hill coefficients) are not close to 1, as this quantification is theoretically justifiable only when the slope factor is close to 1.
- ΔΔLog(τ/K_A_) values are preferred over ΔΔLog (E_max_/EC_50_) when ligand concentration–response curve slope factors (Hill coefficients) are not close to 1.
- Confirm that the calculated bias factors are consistent with a bias plot (Figure 2). There is unlikely to be bias in a system if it cannot be visualized in a bias plot. Although a bias plot only provides a qualitative assessment of bias, it does not have the weakness of relying on fit parameters that can have artificially low errors from fitting routines.

#### 6.2.2 Reporting recommendations

- Report ligand E_max_, EC_50_, and τ/K_A_ values and system maximum response, Em. We recommend authors to tabulate the underlying quantitative values of the pharmacological parameters. Reporting these values will improve clarity, increase transparency and future-proof cross-study comparisons. It also allows the calculation and presentation of multiple models of bias or to change the choice of reference ligand to facilitate comparison across studies.
- Report the ligand concentration–response curve slope factors (Hill coefficients). This information is critical, especially when ΔΔLog (E_max_/EC_50_) is used to assess bias, as slope factors deviating from 1 indicate the extent to which system bias contributes to the calculated bias factors.
- Report a bias plot for biased ligands. This will serve to validate quantified ligand bias in a qualitative and visualized manner.

**Further reading:** For further guides on how to calculate ΔΔLog (E_max_/EC_50_) and ΔΔLog(τ/K_A_) values, we refer the reader to (Kenakin, 2015b; Kenakin & Christopoulos, 2013; Rajagopal et al., 2011).

**Caution:** A large degree of caution is advisable for describing ligands with only weak bias (low bias factor) or absolute efficacy (low E_max_), as these compounds are more likely to produce system-dependent bias effects (see Section 7.1). Such agonists are therefore more likely to be spuriously identified as biased, as both methods outlined above rely on best-fit parameters. Weak partial agonists will result in relatively poor fits (but still with excellent R^2^) with E_max_/EC_50_ or τ/K_A_ values that grossly underestimate the errors of the derived bias factors. One can use a bias plot to confirm non-quantitatively that bias exists between two compounds, but one should never rely on bias factors alone.

### 6.3 Comparing ligand bias across studies and systems (use of rank orders)

#### 6.3.1 Problems and pitfalls

Bias values obtained from different experimental systems are not comparable on a quantitative level. For example, a bias value above 2.0 in one system may be below 2.0 for the same pathways when studied in another system differing by, for example, cell line, measured molecules or process (Figure 3). Achieving a more consistent assessment of which ligand is the most biased towards a given pathway is important to identify functionally selective probes that can be used to dissect a distinct effect. This provides information about which pathways should be targeted or avoided in the design of drugs with higher efficacy and fewer side effects.

#### 6.3.2 Recommendations for experimental design

- We recommend using ligand rank orders of bias factors (rather than quantitative bias values) for comparisons of ligand bias across studies using different experimental systems. Only the pathways studied in both studies/systems can be compared, and the pathway ranks must be identical (i.e. the strongest, second strongest, etc. pathway).

**Disclaimer:** The relative ligand bias rank orders may also differ across systems (Figure 3b). However, they differ less than detailed quantitative values.

## 7 Special Recommendations for ‘Tricky’ Ligands

### 7.1 Low efficacy agonists

Ligand bias quantification should be considered together with the quantified strength of intrinsic efficacy of ligands in the relevant signalling pathways. This is because organ sensitivity and relative intrinsic efficacy differences have a large influence on a low-efficacy (for some pathways) ligand, which may appear to be very functionally selective in some organs. However, a signal may ‘appear’ in a more sensitive organ, or in the same organ at a different physiological state.

#### 7.1.1 Problems and pitfalls

- Too low efficacy in one pathway will result in bias factors with very large errors.
- The response may be stronger in more sensitive tissues or assays.

#### 7.1.2 Recommendations for experimental design

- Use ligand pathway rank orders. To avoid misleading conclusions based on low efficacy, one should use rank orders, that is, normalizing within the transducers for each ligand without referencing to other ligands. Only a transducer/pathway rank order change would constitute biased signalling. For example, if ligands A and B share rank order G_s_ > G_q_ >> arrestin they are not biased relative to each other. However, if a third ligand C differs in its rank order, for example, arrestin > G_q_ >> G_s_, it is biased relative to A and B.
- If quantifying bias, use another reference agonist for E_max_ (see Section 3.1.2).

**Caution:** Lack of response in a low-efficacy pathway does not preclude the antagonism of the natural agonist for that pathway. Thus, a physiological bias can be produced by a synthetic ligand’s combined own response (e.g. full agonism in pathway 1) and blockade of the endogenous response (e.g. partial agonist in pathway 2). The outcome of this consideration is that, although ligand physiology-bias or pathway-bias may not be concluded for a low-efficacy compound, it could still result in functional selectivity that may have physiological importance in some tissues in which a low efficacy compound may simply not be efficacious enough to activate all the pathways. Such a situation would therefore result in a different physiological outcome from that of a compound able to activate all these pathways in the same tissue.

### 7.2 Inverse agonists

Inverse agonists inhibiting the non-ligand-dependent constitutive activity of a receptor may, as biased agonists do, act differentially on pathways by stabilizing distinct receptor conformations. This is only evident for receptors with constitutive activity in the absence of an agonist. The minimum condition needed to quantify bias would be concentration–response curves in two pathways and this condition can be met for an inverse agonist ligand. For receptors with a high constitutive activity, biased inverse agonism could be valuable to fundamentally understand signalling and to exploit this knowledge therapeutically.

#### 7.2.1 Recommendations for experimental design

- Use another inverse agonist as the reference ligand. When a ligand acts as an inverse agonist in two pathways compared for bias, a bias factor can, in theory, be calculated in the same way as for agonists, but using an inverse agonist reference. As most endogenous ligands are agonists, this typically precludes quantification of ligand physiology-bias (exceptions exist, for example, agouti is a rare example of an endogenous inverse agonist).

**Further reading:** Methods to quantify bias for inverse agonists are beyond the scope of this paper, as they involve differences in agonist-mediated and constitutive activity-mediated efficacy (Ehlert, Suga, & Griffin, 2011). It is known that constitutively active receptors themselves possess an efficacy that can be different from agonist-mediated efficacy. This is manifested as the phenomenon of protean agonism whereby a low efficacy partial agonist demonstrates positive agonism in quiescent systems and inverse agonism in constitutively active systems (Chidiac, Nouet, & Bouvier, 1996; Kenakin, 1997). This is because the agonist-mediated active state is of lower efficacy than the constitutively active state. Such phenomena must be considered to ascribe an efficacy to an inverse agonist.

### 7.3 Agonist and antagonist across pathways (‘modality bias’)

#### 7.3.1 Problems and pitfalls

When agonism and no agonism (neutral antagonism or inverse agonism), respectively, are observed in two pathways compared, it is not possibly to quantify bias using the above models. This is because calculation of a quantitated bias factor requires two concentration–response curves with the same modality (agonism or inverse agonism).

#### 7.3.2 Reporting recommendations

- Ligand bias with opposite modalities across pathways can be described as a non-quantitative term, ‘modality bias’. In this case, there is no need to quantify bias to claim bias.
- Alternatively, it can be approximated by measuring an affinity to limit bias or describe it in a ‘bias is larger than’ relationship (Kenakin, 2015a; Stahl, Ehlert, & Bohn, 2019; Stahl, Zhou, Ehlert, & Bohn, 2015). Specifically, the affinity (determined from functional antagonism) is used to determine receptor occupancy and a very low level of efficacy is assumed to generate a simulated curve (i.e. maximal response of 5%) which is then used to calculate bias. This yields the lowest possible bias (it could be greater than this if the efficacy is lower than the assumed one giving 5% maximal response).

**Caution:** Although a very low ligand efficacy cannot be detected in one system, it may be detected in another functional system with higher sensitivity. Hence, the statement should be understood as a practical qualification, in the sense that the efficacy of the ligand is close to zero within the detection limits of the given system. That is, some partial agonists may appear to be neutral antagonists.

**Terminology summary I**

***Modality bias:*** Ligand with efficacy in only one of the compared pathways (neutral antagonist or inverse agonist in others). As for all biased ligands, this refers to a particular system and in another more sensitive system, there may be efficacy at several compared pathways allowing the determination of concentration–response curves. We discourage the use of the previous terms ‘full bias’ or ‘perfect bias’, as they can misleadingly give the impression that the ligand always has full efficacy in the preferred pathway. In fact, a ligand with modality bias may give a weaker activation than other agonists for which bias can be quantified (as they induce agonism in at least two pathways).

### 7.4 Allosteric modulators affecting ligand bias

Pure allosteric modulators will modulate the orthosteric ligand binding and/or efficacy, whereas ago-allosteric modulators also induce signalling of their own. In all cases, the functional outcome can vary depending on the orthosteric ligands.

#### 7.4.1 Problems and pitfalls

No concentration–response curves can be measured for an allosteric ligand on its own; thus, bias cannot be attributed to it individually but must be defined together with the orthosteric ligand.

#### 7.4.2 Recommendations for experimental design

- Quantification of allosteric modulator bias should make use of an extended operational model of allosterism, which was originally described in Leach, Sexton, and Christopoulos (2007) and further extended in Slosky, Caron, and Barak (2021).

#### 7.4.3 Reporting recommendations

- Report both the allosteric modulator and orthosteric ligand.

**Further reading:** For further reading, including examples of biased allosteric modulators see Slosky, Caron, and Barak (2021).

## 8 Publication and Database Deposition

### 8.1 Unambiguous description of ligand bias

All the above information is necessary to interpret ligand bias correctly and to separate it from system bias (Figure 1). Hence, the mention of a ‘biased’ or ‘unbiased’ ligand as short terms are only meaningful after the *prior* definition of this information.

#### 8.1.1 Problems and pitfalls

Ligand bias will not be reproducible and understood consistently without sufficient information about the ligands, pathways and systems. Such ambiguity hampers consensus and advances in the field.

#### 8.1.2 Reporting recommendation

- Define the ligand evoking bias and its system in a clear sentence. This should be at first mention in a manuscript/text. For example, *ligand L displays [recruitment/signalling …] bias towards pathway P1 at time point TP1 over pathway P2 at time point TP2 and relative to reference ligand A at receptor R in cell line C*.
- Tabulate the experimental details required for unambiguous description (Table 3 can be used as a template).
- Deposit biased ligands in a database. GPCRdb offers authors the ability to submit biased ligands, compliant with the reporting recommendations brought forward above. The submission is done via a standardized Excel file detailing all necessary information (https://docs.gpcrdb.org/BiasedSignaling.html#data_submission).

## 9 Conclusion and Outlook

Adopting the guidelines presented here will facilitate consensus and advances in the GPCR field through increased quality, reproducibility and clarity of ligand-induced bias. More precise experiments will improve individual studies and make them more useful for others, as data become more comparable. Furthermore, the unambiguous and structured reporting of bias data will be directly reflected in more relevant databases and literature reviews.

To unlock the rational targeting of GPCRs by exploiting ligand bias to achieve functional selectivity, we must first map individual or combined pathways to their distinct effects. This requires identification of many more probes inducing bias, pharmacological assays, animal models, and so on. This represents a tremendous challenge, but one with extraordinary potential. The recommendations herein can contribute to this massive endeavour by better description of such probes and effects. We have deliberately left the door open to include additional intracellular transducers, effectors, or modulators involved in GPCR signalling, and our definitions should therefore be future-proofed, in the sense that they can be applied also to signalling proteins that have yet to be discovered.

Ligand-dependent biased function is also being investigated for other protein classes, for example, receptor TKs (Karl, Paul, Pasquale, & Hristova, 2020), nuclear receptors (Heidari et al., 2019), monoamine transporters (Hasenhuetl, Bhat, Freissmuth, & Sandtner, 2019), Toll-like receptors (unpublished), and cytochrome P450 enzymes (Jensen et al., 2021). Many of the recommendations brought forward in this paper are also applicable to these protein families, which would in time benefit from publication of additional dedicated guidelines.

### 9.1 Nomenclature of targets and ligands

Key protein targets and ligands in this article are hyperlinked to corresponding entries in https://www.guidetopharmacology.org, and are permanently archived in the Concise Guide to PHARMACOLOGY 2021/22 (Alexander, Christopoulos et al., 2021; Alexander, Fabbro, et al., 2021; Alexander, Kelly et al., 2021).

## Supplementary Material

Appendix

## Figures and Tables

**Table 1:** Types of ligand bias and their meaning by choice of reference ligand

Type of bias	Reference ligand	Meaning (what can be concluded from data)	Disclaimer (meanings/conclusion not supported by data)
Ligand benchmark-bias	Any ligand, for example, a candidate drug or tool compound. The reference ligand can be arbitrarily chosen, but often has a particular relevance as tool or clinical agent and is therefore selected to benchmark other tested ligands.	Simultaneous comparison across pathways and ligands where the reference ligand can be any ligand of choice.	A biased ligand for which the reference ligand was not selected based on specific signalling pathway qualities has bias only relative to the reference ligand, which in turn can elicit any bias.
Ligand pathway-bias	Pathway-balanced ligand	Signalling preferentially via one pathway, as the reference ligand approximates a pathway-balanced signal.	A pathway-balanced/unbiased ligand can be physiology-biased, although it is by definition unbiased in the pathway definition. A balanced ligand in one system may not be ‘balanced’ in another (applies to all types of ligand bias).
Ligand physiology-bias	Principal endogenous agonist	Signalling differs from the physiological, as the reference ligand represents the endogenous response of the given receptor and system.	An endogenous agonist can be pathway-biased, although it is by definition unbiased in the physiological definition.

*Note*: The terms ‘ligand pathway-bias’ and ‘ligand physiology-bias’ are recommended when researchers wish to attribute a specific function (in addition to just a difference to the reference ligand employed).

**Table 2:** Suggested terms to define the pathway processes at different levels being compared for bias

Term	Examples of assay principles
Accumulation	Ca^2+^ or cAMP accumulation
Binding/coupling	Proximity
Activation	Receptor conformation and G protein activation, for example, GTPase activity, GTPγS binding, or recruitment of downstream effectors.
Dissociation	G protein dissociation/rearrangement
Phosphorylation	Incorporation of ^32^P in a specific protein. Two-dimensional phosphopeptide mapping after orthophosphate labelling, MS-based identification of phosphorylation sites, Phosphosite-specific antibody assays or arrays
Recruitment	Subcellular redistribution
Signalling	Downstream cellular second messenger levels
Internalization	ELISA, flow cytometry, diffusion-enhanced resonance energy transfer (DERET), enhanced bystander BRET (ebBRET), (confocal) microscopy, antibody-feeding experiments, real-time internalization by SNAP-tagged receptors
Tissue phenotype	Blood vessel contraction, cell migration, hormone secretion, heart rate, force of contraction, glandular secretion.
Transcription	Differential gene arrays after agonism, qPCR, and gene reporter assay.

*Note*: For specific assays implementing the tabulated assay principles, we refer readers to Bohn & McDonald (2010); Bohn, Zhou & Ho (2015); Masuho, Martemyanov & Lambert (2015); Perry-Hauser, Asher, Hauge Pedersen & Javitch (2021); Wright & Bouvier (2021)

**Table 3:** Experimental parameters critical to the unambiguous description of ligand bias

Parameter	Pathway 1	Pathway 2	Pathway 3
Transducer (‘pathway’)	β-Arrestin 2	G_i_	G_q_
Cell line	CHO	CHO	CHO
Receptor expression levels (fmol·mg^–1^ protein)	300	300	300
Time point for data collection (min) (for time-resolved, a span)	15–60	30	30
Temperature (°C)	20	20	20
Reference ligand for bias	CP55,940	CP55,940	CP55,940
Reference ligand for E_max_	CP55,940	CP55,940	CP55,940
Measured process	Recruitment	Accumulation	Accumulation
Measured molecule 1^a^	CB_1_ receptor	cAMP	Inositol 1-phosphate
Measured molecule 2^a^ (if any)	β-Arrestin 2	-	-
Signal detection technique	FRET	HTRF	HTRF

*Note*: Table entries are examples only.

a A measured molecule is a GPCR, transducer, effector or second messenger.

## Box 1 Ligand bias factor calculation using ΔΔLog(E_max_/EC_50_) or ΔΔLog(*τ*/K_A_).

**ΔΔLog(E_max_/EC_50_) (relative activity)**

1) Ligand Log(E_max_/EC_50_) is calculated using E_max_ in the form of fractions and not percent (e.g. 0.997, not 99.7) and potency values expressed in molar (mole/liter).

$$\log{\left(\frac{Emax}{EC_{50}}\right)}_{lig}$$

2) ΔLog(E_max_/EC_50_) values are calculated for each pathway by subtracting the Log(E_max_/EC_50_) of the reference ligand from that of the tested ligand.

$$\Delta \log\left(\frac{Emax}{EC_{50}}\right)=\log{\left(\frac{Emax}{EC_{50}}\right)}_{lig}-\log{\left(\frac{Emax}{EC_{50}}\right)}_{ref}$$

3) ΔΔLog(E_max_/EC_50_) is calculated by subtracting the ΔLog(E_max_/EC_50_) of pathway P2 from the ΔLog(E_max_/EC_50_) of pathway P1, which has the strongest activation.

$$\Delta \Delta \log\left(\frac{Emax}{EC_{50}}\right)=\text{Δ}\log{\left(\frac{Emax}{EC_{50}}\right)}_{P1}-\text{Δ}\log{\left(\frac{Emax}{EC_{50}}\right)}_{P2}$$

4) Finally, the bias factor is the antilog of the ΔΔLog(E_max_/EC_50_) value. For example, a bias factor of 1 corresponds to a 10-fold activation difference.

$$\text{Bias}\;\text{factor}=10^{\Delta \Delta \log\left(\frac{Emax}{EC_{50}}\right)}$$

**ΔΔLog(τ/K_A_) (Black-Leff operational model)**

ΔΔLog(τ/K_A_) is calculated in the same way as ΔΔLog(E_max_/EC_50_) but replacing E_max_/EC_50_ with τ/K_A_ (Kenakin, 2015). τ is the ‘coupling efficiency’ between the agonist/receptor complex and its downstream signaling partners, and K_A_ is the equilibrium dissociation constant of the agonist-receptor complex. Often no unique values can be calculated for τ and K_A_, which is why the ratio τ/K_A_ is used instead. The fraction τ/K_A_ is an index of agonism termed transduction coefficient. It is solved by fitting the below Black/Leff equation to data using a software, e.g., GraphPad Prism, and setting starting values for τ and K_A_. For partial agonists, the EC_50_ of the curve is a good starting point for K_A_. For full agonists, a useful strategy is to enter 100*EC_50_ for the fitting procedure.

$$\text{Response}=\frac{{\left[\text{A}\right]}^{n}\tau^{n}\text{Em}}{{\left[\text{A}\right]}^{n}\tau^{n}+{\left(\left[\text{A}\right]+{\text{K}}_{A}\right)}^{n}}$$

[A] = Agonist concentration

K_A_= equilibrium dissociation constant of agonist-receptor complex

Response = Agonist response (at the given concentration) as a fraction system maximum response, Em.

System-specific variables (common for all agonists in the assay):

n = Hill slope of the concentration-response-curve

Em = the maximal response of the system induced by a full agonist.

Bias factor calculations using the above equations in the same order (grey shading). This example investigates pathway-bias using Salvinorin A as the reference ligand, as it has a bias plot slope close to 1 (Figure 2 and (White et al., 2014)). A table template with formulae is available in the section Biased signaling – Guidelines in GPCRdb.

**Table:**

Ligand	Bias plot slope	G protein				Arrestin				Ligand bias		
		Emax (%)*	EC_50_ (nM)	Log (Emax/EC_50_)	ΔLog (Emax/EC_50_)	Emax (%)*	EC_50_ (nM)	Log (Emax/EC_50_)	ΔLog (Emax/EC_50_)	ΔΔLog (Emax/EC_50_)	Bias factor	For
Salvinorin A	1.0	99.7	5.18	8.3	0.0 (ref.)	97.2	5.75	8.2	0.0 (ref.)	0.0	1.0	- (ref.)
GR 89696	0.6	96.4	0.97	9.0	0.7	92.8	0.26	9.6	1.3	0.6	3.6	Arrestin
ICI 199,441	0.7	101	1.63	8.8	0.5	84.8	0.4	9.3	1.0	0.5	3.2	Arrestin
RB 48	1.8	101	8.82	8.1	-0.2	63.2	143	6.6	-1.6	1.4	26	G protein
RB 64	1.3	101	5.29	8.3	0.0	104	391	6.4	-1.9	1.9	72	G protein

## Abbreviations

CK: casein kinase
CRIP1a: cannabinoid receptor interacting protein 1a
DERET: diffusion-enhanced resonance energy transfer
ebBRET: enhanced bystander BRET
ERNEST: European Research NEtwork on Signal Transduction
GAP: GTPase-activating protein
GEF: guanine nucleotide exchange factor
GRK: GPCR kinase
K_A_: equilibrium dissociation constant of agonist-receptor complex
k_off_: ligand dissociation rate
k_on_: ligand association rate
NC-IUPHAR: Nomenclature and Standards Committee of the International Union of Basic and Clinical Pharmacology
PH: pleckstrin homology domain
RAMP: receptor activity-modulating protein
RGS: regulator of G protein signalling
RH: RGS homology domain
τ: coupling efficiency between the agonist/receptor complex and its downstream signalling partners

## References

[R1] Ahn S, Shenoy SK, Luttrell LM, Lefkowitz RJ (2020). SnapShot: β-arrestin functions. Cell.

[R2] Alexander SP, Christopoulos A, Davenport AP, Kelly E, Mathie A, Peters JA, Veale EL, Armstrong JF, Faccenda E, Harding SD, Pawson AJ (2021). THE CONCISE GUIDE TO PHARMACOLOGY 2021/22: G protein-coupled receptors. British Journal of Pharmacology.

[R3] Alexander SP, Fabbro D, Kelly E, Mathie A, Peters JA, Veale EL, Armstrong JF, Faccenda E, Harding SD, Pawson AJ, Southan C (2021). THE CONCISE GUIDE TO PHARMACOLOGY 2021/22: Enzymes. British Journal of Pharmacology.

[R4] Alexander SP, Kelly E, Mathie A, Peters JA, Veale EL, Armstrong JF, Faccenda E, Harding SD, Pawson AJ, Southan C, Buneman OP (2021). THE CONCISE GUIDE TO PHARMACOLOGY 2021/22: Other Protein Targets. British Journal of Pharmacology.

[R5] Anderson A, Masuho I, De Velasco EM, Nakano A, Birnbaumer L, Martemyanov KA, Wickman K (2020). GPCR-dependent biasing of GIRK channel signaling dynamics by RGS6 in mouse sinoatrial nodal cells. Proceedings of the National Academy of Sciences of the United States of America.

[R6] Avet C, Mancini A, Breton B, Gouill CL, Hauser AS, Normand C, Kobayashi H, Gross F, Hogue M, Lukasheva V, St-Onge S (2021). Effector membrane translocation biosensors reveal G protein and B-arrestin profiles of 100 therapeutically relevant GPCRs. bioRxiv.

[R7] Azzi M, Charest PG, Angers S, Rousseau G, Kohout T, Bouvier M, Pineyro G (2003). β-Arrestin-mediated activation of MAPK by inverse agonists reveals distinct active conformations for G protein-coupled receptors. Proceedings of the National Academy of Sciences of the United States of America.

[R8] Baker JG, Hall IP, Hill SJ (2003). Agonist and inverse agonist actions of β-blockers at the human β2-adrenoceptor provide evidence for agonist-directed signaling. Molecular Pharmacology.

[R9] Barlow RB, Scott NC, Stephenson RP (1967). The affinity and efficacy of onium salts on the frog rectus abdominis. British Journal of Pharmacology and Chemotherapy.

[R10] Black JW, Leff P (1983). Operational models of pharmacological agonism. Proceedings of the Royal Society of London - Series B: Biological Sciences.

[R11] Bock A, Bermudez M (2021). Allosteric coupling and biased agonism in G protein-coupled receptors. The FEBS Journal.

[R12] Bockaert J, Fagni L, Dumuis A, Marin P (2004). GPCR interacting proteins (GIP). Pharmacology & Therapeutics.

[R13] Bohn LM, McDonald PH (2010). Seeking ligand bias: Assessing GPCR coupling to β-arrestins for drug discovery. Drug Discovery Today: Technologies.

[R14] Bohn LM, Zhou L, Ho JH (2015). Approaches to assess functional selectivity in GPCRs: Evaluating G protein signaling in an endogenous environment. Methods in Molecular Biology.

[R15] Bouvier M, Leeb-Lundberg LM, Benovic JL, Caron MG, Lefkowitz RJ (1987). Regulation of adrenergic receptor function by phosphorylation. II. Effects of agonist occupancy on phosphorylation of α1- and β2-adrenergic receptors by protein kinase C and the cyclic AMP-dependent protein kinase. The Journal of Biological Chemistry.

[R16] Bradley SJ, Tobin AB (2016). Design of next-generation G protein-coupled receptor drugs: Linking novel pharmacology and in vivo animal models. Annual Review of Pharmacology and Toxicology.

[R17] Carman CV, Parent JL, Day PW, Pronin AN, Sternweis PM, Wedegaertner PB, Gilman AG, Benovic JL, Kozasa T (1999). Selective regulation of Gα(q/11) by an RGS domain in the G proteincoupled receptor kinase, GRK2. The Journal of Biological Chemistry.

[R18] Che T, Dwivedi-Agnihotri H, Shukla AK, Roth BL (2021). Biased ligands at opioid receptors: Current status and future directions. Science Signaling.

[R19] Chidiac P, Nouet S, Bouvier M (1996). Agonist-induced modulation of inverse agonist efficacy at the β2-adrenergic receptor. Molecular Pharmacology.

[R20] Crilly SE, Puthenveedu MA (2021). Compartmentalized GPCR signaling from intracellular membranes. The Journal of Membrane Biology.

[R21] De Lean A, Stadel JM, Lefkowitz RJ (1980). A ternary complex model explains the agonist-specific binding properties of the adenylate cyclase-coupled β-adrenergic receptor. The Journal of Biological Chemistry.

[R22] Dean T, Vilardaga JP, Potts JT, Gardella TJ (2008). Altered selectivity of parathyroid hormone (PTH) and PTH-related protein (PTHrP) for distinct conformations of the PTH/PTHrP receptor. Molecular Endocrinology.

[R23] DebBurman SK, Ptasienski J, Benovic JL, Hosey MM (1996). G protein-coupled receptor kinase GRK2 is a phospholipid-dependent enzyme that can be conditionally activated by G protein βγ subunits. The Journal of Biological Chemistry.

[R24] Dorn GW, Oswald KJ, McCluskey TS, Kuhel DG, Liggett SB (1997). α2A-Adrenergic receptor stimulated calcium release is transduced by Gi-associated G (βγ)-mediated activation of phospholipase C. Biochemistry.

[R25] Ehlert FJ (2008). On the analysis of ligand-directed signaling at G protein-coupled receptors. Naunyn-Schmiedeberg’s Archives of Pharmacology.

[R26] Ehlert FJ, Suga H, Griffin MT (2011). Analysis of agonism and inverse agonism in functional assays with constitutive activity: Estimation of orthosteric ligand affinity constants for active and inactive receptor states. The Journal of Pharmacology and Experimental Therapeutics.

[R27] Eichel K, Jullie D, Barsi-Rhyne B, Latorraca NR, Masureel M, Sibarita JB, Dror RO, von Zastrow M (2018). Catalytic activation of β-arrestin by GPCRs. Nature.

[R28] Eichel K, Jullie D, von Zastrow M (2016). β-Arrestin drives MAP kinase signalling from clathrin-coated structures after GPCR dissociation. Nature Cell Biology.

[R29] Feinstein TN, Wehbi VL, Ardura JA, Wheeler DS, Ferrandon S, Gardella TJ, Vilardaga JP (2011). Retromer terminates the generation of cAMP by internalized PTH receptors. Nature Chemical Biology.

[R30] Furchott RF, Harper NJAS (1996). Adv drug res.

[R31] Galandrin S, Oligny-Longpre G, Bouvier M (2007). The evasive nature of drug efficacy: Implications for drug discovery. Trends in Pharmacological Sciences.

[R32] Gales C, Van Durm JJ, Schaak S, Pontier S, Percherancier Y, Audet M, Paris H, Bouvier M (2006). Probing the activation-promoted structural rearrangements in preassembled receptor-G protein complexes. Nature Structural & Molecular Biology.

[R33] Ghosh E, Dwivedi H, Baidya M, Srivastava A, Kumari P, Stepniewski T, Kim HR, Lee MH, van Gastel J, Chaturvedi M, Roy D (2019). Conformational sensors and domain swapping reveal structural and functional differences between β-arrestin isoforms. Cell Reports.

[R34] Gomes I, Sierra S, Lueptow L, Gupta A, Gouty S, Margolis EB, Cox BM, Devi LA (2020). Biased signaling by endogenous opioid peptides. Proceedings of the National Academy of Sciences of the United States of America.

[R35] Grundmann M, Merten N, Malfacini D, Inoue A, Preis P, Simon K, Rüttiger N, Ziegler N, Benkel T, Schmitt NK, Ishida S (2018). Lack of β-arrestin signaling in the absence of active G proteins. Nature Communications.

[R36] Gurevich VV, Benovic JL (1997). Mechanism of phosphorylation-recognition by visual arrestin and the transition of arrestin into a high affinity binding state. Molecular Pharmacology.

[R37] Gurevich VV, Gurevich EV (2018). Arrestin-mediated signaling: Is there a controversy?. World Journal of Biological Chemistry.

[R38] Gutkind JS, Kostenis E (2018). Arrestins as rheostats of GPCR signalling. Nature Reviews Molecular Cell Biology.

[R39] Harding SD, Armstrong JF, Faccenda E, Southan C, Alexander SPH, Davenport AP, Pawson AJ, Spedding M, Davies JA, NC-IUPHAR (2021). The IUPHAR/BPS guide to pharmacology in 2022: Curating pharmacology for COVID-19, malaria and antibacterials. Nucleic Acids Research.

[R40] Hasenhuetl PS, Bhat S, Freissmuth M, Sandtner W (2019). Functional selectivity and partial efficacy at the monoamine transporters: A unified model of allosteric modulation and amphetamine-induced substrate release. Molecular Pharmacology.

[R41] Hausdorff WP, Bouvier M, O’Dowd BF, Irons GP, Caron MG, Lefkowitz RJ (1989). Phosphorylation sites on two domains of the β2-adrenergic receptor are involved in distinct pathways of receptor desensitization. The Journal of Biological Chemistry.

[R42] Hauser AS, Attwood MM, Rask-Andersen M, Schioth HB, Gloriam DE (2017). Trends in GPCR drug discovery: New agents, targets and indications. Nature Reviews. Drug Discovery.

[R43] Hay DL, Garelja ML, Poyner DR, Walker CS (2018). Update on the pharmacology of calcitonin/CGRP family of peptides: IUPHAR review 25. British Journal of Pharmacology.

[R44] Hay DL, Pioszak AA (2016). Receptor activity-modifying proteins (RAMPs): New insights and roles. Annual Review of Pharmacology and Toxicology.

[R45] Heidari Z, Chrisman IM, Nemetchek MD, Novick SJ, Blayo AL, Patton T, Mendes DE, Diaz P, Kamenecka TM, Griffin PR, Hughes TS (2019). Definition of functionally and structurally distinct repressive states in the nuclear receptor PPARγ. Nature Communications.

[R46] Hillenbrand M, Schori C, Schoppe J, Plückthun A (2015). Comprehensive analysis of heterotrimeric G-protein complex diversity and their interactions with GPCRs in solution. Proceedings of the National Academy of Sciences of the United States of America.

[R47] Ho MKC, Wong YH (2001). Gz signaling: Emerging divergence from Gi signaling. Oncogene.

[R48] Hoare SRJ, Pierre N, Moya AG, Larson B (2018). Kinetic operational models of agonism for G-protein-coupled receptors. Journal of Theoretical Biology.

[R49] Hollenberg MD, Mihara K, Polley D, Suen JY, Han A, Fairlie DP, Ramachandran R (2014). Biased signalling and proteinase-activated receptors (PARs): Targeting inflammatory disease. British Journal of Pharmacology.

[R50] Hollinger S, Hepler JR (2002). Cellular regulation of RGS proteins: Modulators and integrators of G protein signaling. Pharmacological Reviews.

[R51] Hunton DL, Barnes WG, Kim J, Ren XR, Violin JD, Reiter E, Milligan G, Patel DD, Lefkowitz RJ (2005). β-Arrestin 2-dependent angiotensin II type 1A receptor-mediated pathway of chemotaxis. Molecular Pharmacology.

[R52] Inoue A, Raimondi F, Kadji FMN, Singh G, Kishi T, Uwamizu A, Ono Y, Shinjo Y, Ishida S, Arang N, Kawakami K (2019). Illuminating G-protein-coupling selectivity of GPCRs. Cell.

[R53] Irannejad R, Pessino V, Mika D, Huang B, Wedegaertner PB, Conti M, von Zastrow M (2017). Functional selectivity of GPCR-directed drug action through location bias. Nature Chemical Biology.

[R54] Jain R, Watson U, Vasudevan L, Saini DK, Shukla AK (2018). Int rev cell Mol biol.

[R55] Jensen DD, Lieu T, Halls ML, Veldhuis NA, Imlach WL, Mai QN, Poole DP, Quach T, Aurelio L, Conner J, Herenbrink CK (2017). Neurokinin 1 receptor signaling in endosomes mediates sustained nociception and is a viable therapeutic target for prolonged pain relief. Science Translational Medicine.

[R56] Jensen SB, Thodberg S, Parween S, Moses ME, Hansen CC, Thomsen J, Sletfjerding MB, Knudsen C, del Giudice R, Lund PM, Castaño PR (2021). Biased cytochrome P450-mediated metabolism via small-molecule ligands binding P450 oxidoreductase. Nature Communications.

[R57] Jiang M, Bajpayee NS (2009). Molecular mechanisms of go signaling. Neurosignals.

[R58] Jong YI, Harmon SK, O’Malley KL (2019). Location and cell-type-specific bias of metabotropic glutamate receptor, mGlu5, negative allosteric modulators. ACS Chemical Neuroscience.

[R59] Karl K, Paul MD, Pasquale EB, Hristova K (2020). Ligand bias in receptor tyrosine kinase signaling. The Journal of Biological Chemistry.

[R60] Kenakin T (1995). Agonist-receptor efficacy. II. Agonist trafficking of receptor signals. Trends in Pharmacological Sciences.

[R61] Kenakin T (1997). Differences between natural and recombinant G protein-coupled receptor systems with varying receptor/G protein stoichiometry. Trends in Pharmacological Sciences.

[R62] Kenakin T (2015a). The effective application of biased signaling to new drug discovery. Molecular Pharmacology.

[R63] Kenakin T (2015b). The measurement of receptor signaling bias. Methods in Molecular Biology.

[R64] Kenakin T (2019). Biased receptor signaling in drug discovery. Pharmacological Reviews.

[R65] Kenakin T, Christopoulos A (2013). Signalling bias in new drug discovery Detection, quantification and therapeutic impact. Nature Reviews Drug Discovery.

[R66] Kenakin TP, Morgan PH (1989). Theoretical effects of single and multiple transducer receptor coupling proteins on estimates of the relative potency of agonists. Molecular Pharmacology.

[R67] Klein Herenbrink C, Sykes DA, Donthamsetti P, Canals M, Coudrat T, Shonberg J, Scammells PJ, Capuano B, Sexton PM, Charlton SJ, Javitch JA (2016). The role of kinetic context in apparent biased agonism at GPCRs. Nature Communications.

[R68] Kohout TA, Nicholas SL, Perry SJ, Reinhart G, Junger S, Struthers RS (2004). Differential desensitization, receptor phosphorylation, β-arrestin recruitment, and ERK1/2 activation by the two endogenous ligands for the CC chemokine receptor 7. The Journal of Biological Chemistry.

[R69] Komolov KE, Benovic JL (2018). G protein-coupled receptor kinases: Past, present and future. Cellular Signalling.

[R70] Kooistra AJ, Mordalski S, Pandy-Szekeres G, Esguerra M, Mamyrbekov A, Munk C, Keseru GM, Gloriam DE (2021). GPCRdb in 2021: Integrating GPCR sequence, structure and function. Nucleic Acids Research.

[R71] Lane JR, May LT, Parton RG, Sexton PM, Christopoulos A (2017). A kinetic view of GPCR allostery and biased agonism. Nature Chemical Biology.

[R72] Leach K, Sexton PM, Christopoulos A (2007). Allosteric GPCR modulators: Taking advantage of permissive receptor pharmacology. Trends in Pharmacological Sciences.

[R73] Lefkowitz RJ, Mullikin D, Caron MG (1976). Regulation of β-adrenergic receptors by guanyl-5′-yl imidodiphosphate and other purine nucleotides. The Journal of Biological Chemistry.

[R74] Lukasheva V, Devost D, Le Gouill C, Namkung Y, Martin RD, Longpré J-M, Amraei M, Shinjo Y, Hogue M, Lagacé M, Breton B (2020). Signal profiling of the β1AR reveals coupling to novel signalling pathways and distinct phenotypic responses mediated by β1AR and β2AR. Scientific Reports.

[R75] Luttrell LM, Maudsley S, Gesty-Palmer D (2018). Translating in vitro ligand bias into in vivo efficacy. Cellular Signalling.

[R76] Luttrell LM, Roudabush FL, Choy EW, Miller WE, Field ME, Pierce KL, Lefkowitz RJ (2001). Activation and targeting of extracellular signal-regulated kinases by β-arrestin scaffolds. Proceedings of the National Academy of Sciences of the United States of America.

[R77] Luttrell LM, Wang J, Plouffe B, Smith JS, Yamani L, Kaur S, Jean-Charles PY, Gauthier C, Lee MH, Pani B, Kim J (2018). Manifold roles of β-arrestins in GPCR signaling elucidated with siRNA and CRISPR/Cas9. Science Signaling.

[R78] Maguire ME, Van Arsdale PM, Gliman AG (1976). An agonist-specific effect of guanine nucleotides on binding to the β-adrenergic receptor. Molecular Pharmacology.

[R79] Masuho I, Balaji S, Muntean BS, Skamangas NK, Chavali S, Tesmer JJG, Babu MM, Martemyanov KA (2020). A global map of G protein signaling regulation by RGS proteins. Cell.

[R80] Masuho I, Martemyanov KA, Lambert NA (2015). Monitoring G protein activation in cells with BRET. Methods in Molecular Biology.

[R81] Masuho I, Ostrovskaya O, Kramer GM, Jones CD, Xie K, Martemyanov KA (2015). Distinct profiles of functional discrimination among G proteins determine the actions of G protein-coupled receptors. Science Signaling.

[R82] Masuho I, Skamangas NK, Muntean BS, Martemyanov KA (2021). Diversity of the Gβγ complexes defines spatial and temporal bias of GPCR signaling. Cell Systems.

[R83] Maurice P, Guillaume JL, Benleulmi-Chaachoua A, Daulat AM, Kamal M, Jockers R (2011). GPCR-interacting proteins major players of GPCR function. Advances in Pharmacology.

[R84] Meyrath M, Szpakowska M, Zeiner J, Massotte L, Merz MP, Benkel T, Simon K, Ohnmacht J, Turner JD, Krüger R, Seutin V (2020). The atypical chemokine recep- tor ACKR3/CXCR7 is a broad-spectrum scavenger for opioid peptides. Nature Communications.

[R85] Milligan G, Kostenis E (2006). Heterotrimeric G-proteins: A short history. British Journal of Pharmacology.

[R86] Namkung Y, Le Gouill C, Lukashova V, Kobayashi H, Hogue M, Khoury E, Song M, Bouvier M, Laporte SA (2016). Monitoring G protein-coupled receptor and β-arrestin trafficking in live cells using enhanced bystander BRET. Nature Communications.

[R87] Namkung Y, LeGouill C, Kumar S, Cao Y, Teixeira LB, Lukasheva V, Giubilaro J, Simões SC, Longpré JM, Devost D, Hébert TE (2018). Functional selectivity profiling of the angiotensin II type 1 receptor using pathway-wide BRET signaling sensors. Science Signaling.

[R88] Neubig RR, Siderovski DP (2002). Regulators of G-protein signalling as new central nervous system drug targets. Nature Reviews Drug Discovery.

[R89] Oliver EE, Hughes EK, Puckett MK, Chen R, Lowther WT, Howlett AC (2020). Cannabinoid receptor interacting protein 1a (CRIP1a) in health and disease. Biomolecules.

[R90] Olsen RHJ, DiBerto JF, English JG, Glaudin AM, Krumm BE, Slocum ST, Che T, Gavin AC, McCorvy JD, Roth BL, Strachan RT (2020). TRUPATH an open-source biosensor platform for interrogating the GPCR transducerome. Nature Chemical Biology.

[R91] Onaran HO, Ambrosio C, Ugur O, Madaras Koncz E, Gro MC, Vezzi V, Rajagopal S, Costa T (2017). Systematic errors in detecting biased agonism: Analysis of current methods and development of a new model-free approach. Scientific Reports.

[R92] Onaran HO, Costa T (2021). Conceptual and experimental issues in biased agonism. Cellular Signalling.

[R93] Pandey S, Kumari P, Baidya M, Kise R, Cao Y, Dwivedi-Agnihotri H, Banerjee R, Li XX, Cui CS, Lee JD, Kawakami K (2021). Intrinsic bias at non-canonical β-arrestin-coupled seven transmembrane receptors. Molecular Cell.

[R94] Pandy-Szekeres G, Esguerra M, Hauser AS, Caroli J, Munk C, Pilger S, Keserű GM, Kooistra AJ, Gloriam DE (2022). The G protein database GproteinDb. Nucleic Acids Research.

[R95] Perry-Hauser NA, Asher WB, Hauge Pedersen M, Javitch JA, Shukla AK (2021). Methods cell biol.

[R96] Rajagopal S, Ahn S, Rominger DH, Gowen-MacDonald W, Lam CM, DeWire SM, Violin JD, Lefkowitz RJ (2011). Quantifying ligand bias at seven-transmembrane receptors. Molecular Pharmacology.

[R97] Rajagopal S, Kim J, Ahn S, Craig S, Lam CM, Gerard NP, Gerard C, Lefkowitz RJ (2010). β-Arrestin- but not G protein-mediated signaling by the “decoy” receptor CXCR7. Proceedings of the National Academy of Sciences of the United States of America.

[R98] Ribas C, Penela P, Murga C, Salcedo A, Garcia-Hoz C, Jurado-Pueyo M, Aymerich I, Mayor F (2007). The G protein-coupled receptor kinase (GRK) interactome: Role of GRKs in GPCR regulation and signaling. Biochimica et Biophysica Acta.

[R99] Roth BL, Chuang DM (1987). Multiple mechanisms of serotonergic signal transduction. Life Sciences.

[R100] Sauliere A, Bellot M, Paris H, Denis C, Finana F, Hansen JT, Altié MF, Seguelas MH, Pathak A, Hansen JL, Sénard JM (2012). Deciphering biased-agonism complexity reveals a new active AT1 receptor entity. Nature Chemical Biology.

[R101] Schmid CL, Raehal KM, Bohn LM (2008). Agonist-directed signaling of the serotonin 2A receptor depends on β-arrestin-2 interactions in vivo. Proceedings of the National Academy of Sciences of the United States of America.

[R102] Slosky LM, Caron MG, Barak LS (2021). Biased allosteric modulators: New frontiers in GPCR drug discovery. Trends in Pharmacological Sciences.

[R103] Smith JS, Lefkowitz RJ, Rajagopal S (2018). Biased signalling: From simple switches to allosteric microprocessors. Nature Reviews Drug Discovery.

[R104] Smith JS, Pack TF, Inoue A, Lee C, Zheng K, Choi I, Eiger DS, Warman A, Xiong X, Ma Z, Viswanathan G (2021). Noncanonical scaffolding of Gαi and β-arrestin by G protein-coupled receptors. Science.

[R105] Sommer ME, Selent J, Carlsson J, De Graaf C, Gloriam DE, Keseru GM, Kosloff M, Mordalski S, Rizk A, Rosenkilde MM, Sotelo E (2020). The European Research Network on Signal Transduction (ERNEST): Toward a multidimensional holistic understanding of G protein-coupled receptor signaling. ACS Pharmacology & Translational Science.

[R106] Spengler D, Waeber C, Pantaloni C, Holsboer F, Bockaert J, Seeburg PH, Journot L (1993). Differential signal transduction by five splice variants of the PACAP receptor. Nature.

[R107] Sriram K, Insel PA (2018). G protein-coupled receptors as targets for approved drugs: How many targets and how many drugs?. Molecular Pharmacology.

[R108] Srivastava A, Gupta B, Gupta C, Shukla AK (2015). Emerging functional divergence of β-arrestin isoforms in GPCR function. Trends in Endocrinology and Metabolism.

[R109] Stahl EL, Ehlert FJ, Bohn LM (2019). Quantitating ligand bias using the competitive model of ligand activity. Methods in Molecular Biology.

[R110] Stahl EL, Zhou L, Ehlert FJ, Bohn LM (2015). A novel method for analyzing extremely biased agonism at G protein-coupled receptors. Molecular Pharmacology.

[R111] Stallaert W, Christopoulos A, Bouvier M (2011). Ligand functional selectivity and quantitative pharmacology at G protein-coupled receptors. Expert Opinion on Drug Discovery.

[R112] Stephenson RP (1956). A modification of receptor theory. British Journal of Pharmacology and Chemotherapy.

[R113] Stout BD, Clarke WP, Berg KA (2002). Rapid desensitization of the serotonin(2C) receptor system: Effector pathway and agonist dependence. The Journal of Pharmacology and Experimental Therapeutics.

[R114] Thomsen ARB, Plouffe B, Cahill TJ, Shukla AK, Tarrasch JT, Dosey AM, Kahsai AW, Strachan RT, Pani B, Mahoney JP, Huang L (2016). GPCR-G protein-β-arrestin super-complex mediates sustained G protein signaling. Cell.

[R115] Tobin AB, Totty NF, Sterlin AE, Nahorski SR (1997). Stimulus-dependent phosphorylation of G-protein-coupled receptors by casein kinase 1α. The Journal of Biological Chemistry.

[R116] Tsvetanova NG, von Zastrow M (2014). Spatial encoding of cyclic AMP signaling specificity by GPCR endocytosis. Nature Chemical Biology.

[R117] Urban JD, Clarke WP, von Zastrow M, Nichols DE, Kobilka B, Weinstein H, Javitch JA, Roth BL, Christopoulos A, Sexton PM, Miller KJ (2007). Functional selectivity and classical concepts of quantitative pharmacology. The Journal of Pharmacology and Experimental Therapeutics.

[R118] Wacker D, Stevens RC, Roth BL (2017). How ligands illuminate GPCR molecular pharmacology. Cell.

[R119] Wehbi VL, Stevenson HP, Feinstein TN, Calero G, Romero G, Vilardaga JP (2013). Noncanonical GPCR signaling arising from a PTH receptor-arrestin-Gβγ complex. Proceedings of the National Academy of Sciences of the United States of America.

[R120] Whalen EJ, Rajagopal S, Lefkowitz RJ (2011). Therapeutic potential of β-arrestin- and G protein-biased agonists. Trends in Molecular Medicine.

[R121] White KL, Scopton AP, Rives ML, Bikbulatov RV, Polepally PR, Brown PJ, Kenakin T, Javitch JA, Zjawiony JK, Roth BL (2014). Identification of novel functionally selective kappa-opioid receptor scaffolds. Molecular Pharmacology.

[R122] Wright SC, Bouvier M (2021). Illuminating the complexity of GPCR pathway selectivity—Advances in biosensor development. Current Opinion in Structural Biology.

