# Community guidelines for GPCR ligand bias: IUPHAR review 32

**DOI:** 10.1111/bph.15811

**Published:** 2022-03-27

**Authors:** Peter Kolb, Terry Kenakin, Stephen P. H. Alexander, Marcel Bermudez, Laura M. Bohn, Christian S. Breinholt, Michel Bouvier, Stephen J. Hill, Evi Kostenis, Kirill A. Martemyanov, Rick R. Neubig, H. Ongun Onaran, Sudarshan Rajagopal, Bryan L. Roth, Jana Selent, Arun K. Shukla, Martha E. Sommer, David E. Gloriam

**Affiliations:** ^1^ Department of Pharmaceutical Chemistry Philipps‐University Marburg Marburg Germany; ^2^ Department of Pharmacology University of North Carolina School of Medicine Chapel Hill North Carolina USA; ^3^ School of Life Sciences University of Nottingham Nottingham UK; ^4^ Department of Pharmaceutical and Medicinal Chemistry University of Münster Münster Germany; ^5^ Department of Molecular Medicine The Scripps Research Institute Jupiter Florida USA; ^6^ Department of Drug Design and Pharmacology University of Copenhagen Copenhagen Denmark; ^7^ Department of Biochemistry and Molecular Medicine, Institute for Research in Immunology and Cancer Université de Montréal Québec Canada; ^8^ Molecular, Cellular, and Pharmacobiology Section, Institute for Pharmaceutical Biology University of Bonn Bonn Germany; ^9^ Department of Neuroscience The Scripps Research Institute Jupiter Florida USA; ^10^ Department of Pharmacology and Toxicology Michigan State University East Lansing Michigan USA; ^11^ Molecular Biology and Technology Development Unit, Department of Pharmacology, Faculty of Medicine Ankara University Ankara Turkey; ^12^ Department of Medicine Duke University Medical Center Durham North Carolina USA; ^13^ Department of Biochemistry Duke University Medical Center Durham North Carolina USA; ^14^ Research Programme on Biomedical Informatics, Hospital Del Mar Medical Research Institute, Department of Experimental and Health Sciences Pompeu Fabra University Barcelona Spain; ^15^ Department of Biological Sciences and Bioengineering Indian Institute of Technology Kanpur India; ^16^ Institute of Medical Physics and Biophysics Charité−Universitätsmedizin Berlin Berlin Germany; ^17^ Present address: Signalling Platform ISAR Bioscience Institute Munich‐Planegg Germany

## Abstract

GPCRs modulate a plethora of physiological processes and mediate the effects of one‐third of FDA‐approved drugs. Depending on which ligand activates a receptor, it can engage different intracellular transducers. This ‘biased signalling’ paradigm requires that we now characterize physiological signalling not just by receptors but by ligand–receptor pairs. Ligands eliciting biased signalling may constitute better drugs with higher efficacy and fewer adverse effects. However, ligand bias is very complex, making reproducibility and description challenging. Here, we provide guidelines and terminology for any scientists to design and report ligand bias experiments. The guidelines will aid consistency and clarity, as the basic receptor research and drug discovery communities continue to advance our understanding and exploitation of ligand bias. Scientific insight, biosensors, and analytical methods are still evolving and should benefit from and contribute to the implementation of the guidelines, together improving translation from in vitro to disease‐relevant in vivo models.

**Abbreviations**
CK: casein kinase
CRIP1a: cannabinoid receptor interacting protein 1a
DERET: diffusion‐enhanced resonance energy transfer
ebBRET: enhanced bystander BRET
ERNEST: European Research NEtwork on Signal Transduction
GAP: GTPase‐activating protein
GEF: guanine nucleotide exchange factor
GRK: GPCR kinase
K_A_
: equilibrium dissociation constant of agonist‐receptor complex
k_off_
: ligand dissociation rate
k_on_
: ligand association rate
NC‐IUPHAR: Nomenclature and Standards Committee of the International Union of Basic and Clinical Pharmacology
PH: pleckstrin homology domain
RAMP: receptor activity‐modulating protein
RGS: regulator of G protein signalling
RH: RGS homology domain
τ: coupling efficiency between the agonist/receptor complex and its downstream signalling partners

## 1 INTRODUCTION

The ~800 human GPCRs transduce sensory inputs and systemic signals into appropriate cellular responses in numerous physiological processes. They recognize a vast diversity of signals ranging from photons, tastants and odours to ions, neurotransmitters, hormones, and cytokines (Harding et al., 2021; Wacker, Stevens, & Roth, 2017). Even though GPCRs represent the primary target of 34% of FDA‐approved drugs, more than 220 non‐olfactory GPCRs have disease associations which are as yet untapped in clinical research (Hauser, Attwood, Rask‐Andersen, Schioth, & Gloriam, 2017; Sriram & Insel, 2018). Despite the diversity of extracellular ligands and physiological roles of GPCRs, these cell surface receptors share a conserved molecular fold and intracellular transducers. Agonist binding stabilizes active conformations of the receptor, facilitating the binding of one or more cytosolic transducer proteins. These include the heterotrimeric G proteins consisting of α, β and γ subunits that dissociate to α and βγ upon activation by the receptor. G proteins comprise 16 distinct α subunits and are divided into four families based on homology and associated downstream signalling pathways: G_s_ (G_s_ and G_olf_), G_i/o_ (G_i1_, G_i2_, G_i3_, G_o_, G_t1_, G_t2_, G_t3_, and G_z_), G_q/11_ (G_q_, G_11_, G_14_ and G_15_), and G_12/13_ (G_12_ and G_13_). Moreover, there are five different β and 12 γ subunit types, resulting in a vast number of possible heterotrimeric G protein combinations (Hillenbrand et al., 2015; Masuho et al., 2021; Milligan & Kostenis, 2006; Olsen et al., 2020).

Activated GPCRs are also bound and phosphorylated at multiple Ser and Thr residues by one or more of the seven different GPCR kinases (GRKs) or effector kinases, such as PKA and PKC. Receptor phosphorylation by GRKs is a key functional determinant for the binding of arrestin proteins (4 subtypes) (Komolov & Benovic, 2018), which can ‘arrest’ signalling by blocking G protein coupling and facilitating receptor internalization, although phosphorylation‐independent arrestin interactions have also been described (Eichel et al., 2018). Arrestins are scaffold proteins that recruit and/or assemble other proteins that signal (Ahn, Shenoy, Luttrell, & Lefkowitz, 2020). Ultimately, the functional interplay between G proteins, GRKs, other kinases, arrestins, and other interaction partners at a GPCR shapes the outcome of receptor signalling in space and time (Gutkind & Kostenis, 2018; Kenakin, 2019). However, the molecular mechanisms underlying these complex and variable interactions remain far from fully understood (Smith et al., 2021; Thomsen et al., 2016).

‘Biased signalling’ is the ligand‐dependent activation of certain pathways over others, and can lead to a ‘functionally selective’ response. ‘Biased signalling’ became generally accepted after evidence accumulated that the rank order of ligands by potency could be different for different pathways engaged by a single receptor (Roth & Chuang, 1987; Spengler et al., 1993) or inversion of the ligand modality (Azzi et al., 2003; Baker, Hall, & Hill, 2003). The most frequently studied pathway‐bias has been that between G proteins and arrestins, while more recent studies have compared G protein families and even subtypes belonging to the same G protein family. Several receptors have evolved to recognize multiple alternative endogenous agonists eliciting biased signalling (relative to the principal endogenous ligand), for example, chemokine (Kohout et al., 2004), opioid (Gomes et al., 2020), PACAP (Spengler et al., 1993), protease‐activated (Hollenberg et al., 2014), 5‐HT (serotonin) (Schmid, Raehal, & Bohn, 2008), and PTH (Dean, Vilardaga, Potts, & Gardella, 2008) receptors. Therapeutic exploitation of biased signalling could increase drug efficacy while avoiding adverse effects attributable to particular pathways. Several studies have outlined disease‐relevant pathways for future therapeutic targeting (Urban et al., 2007; Whalen, Rajagopal, & Lefkowitz, 2011) or retrospective cross‐screening yielding biased ligands predicted to result in potentially useful phenotypes in therapy (Che, Dwivedi‐Agnihotri, Shukla, & Roth, 2021; Galandrin, Oligny‐Longpre, & Bouvier, 2007; Kenakin, 2019; Urban et al., 2007; Whalen, Rajagopal, & Lefkowitz, 2011).

Mechanistically, biased signalling has been explained by conformational selection. Specifically, a ligand and transducer—binding from opposite sides of the cell membrane—may prefer the same receptor structure conformation and thereby allosterically select each other (Galandrin, Oligny‐Longpre, & Bouvier, 2007; Kenakin, 1995; Kenakin & Morgan, 1989; Roth & Chuang, 1987; Smith, Lefkowitz, & Rajagopal, 2018). In other words, this allosteric communication is reciprocal, as G protein pre‐coupling can potentiate agonist binding (Bock & Bermudez, 2021; De Lean, Stadel, & Lefkowitz, 1980; Lefkowitz, Mullikin, & Caron, 1976; Maguire, Van Arsdale, & Gliman, 1976). An activated receptor state has also been linked to a high affinity binding state for arrestin (Gurevich & Benovic, 1997). However, it is still unclear what the precise relationship between conformation and signalling is—at least at the level of detail required to predict such outcomes.

Biased signalling comprises very complex pharmacology making experiment design, interpretation and description challenging and often inconsistent—causing confusion about what has really been measured and what can be concluded. Here, we provide recommendations on ligand bias intended to serve as common guidelines for the field along with defined terminology. They are supported by the authoritative organization for pharmacological nomenclature, the Nomenclature and Standards Committee of the International Union of Basic and Clinical Pharmacology (NC‐IUPHAR, https://www.guidetopharmacology.org/nciuphar.jsp), and COST Action CA18133 ERNEST (European Research NEtwork on Signal Transduction) (Sommer et al., 2020). Each section below provides definitions, terminology and recommendations for experiments and reporting (summarized in Data S1, S2, and S3).

## 2 DEFINITIONS OF PATHWAYS AND OF BIAS TYPES

### 2.1 Pathway definition and modulation

A GPCR pathway is here defined by a transducer protein, or family thereof, binding intracellularly to the receptor and eliciting a distinct cellular downstream signalling cascade, trafficking or internalization. Based on present knowledge, this includes the four G protein families—that is, the G_s_, G_i/o_, G_q/11_, G_12/13_ pathways—and the two GPCR kinase (GRK) and arrestin families (in all six transducer protein families). Typically, there is a sequential engagement of a receptor with a G protein, GRK, and arrestin. However, GRKs and arrestins can be recruited to and bind to GPCRs independently of functionally active G proteins (Grundmann et al., 2018; Hunton et al., 2005; Sauliere et al., 2012; Wehbi et al., 2013). For example, GRK4‐6 functions do not appear to require either G proteins or arrestins, as they are not recruited by G_βγ_ but anchored to the plasma membrane via polybasic domains and lipid modification (Komolov & Benovic, 2018). Ligand bias is not limited to the comparison of these six transducer protein families but can also occur within each such family, that is, across its different members. This extends the number of pathways to 27: 16 Gα proteins, 7 GRKs, and 4 arrestin proteins. For example, G proteins belonging to the same family may differ in their functional outcome due to unique binding kinetics, cellular expression levels, and engagement of different downstream effectors (Anderson et al., 2020; Avet et al., 2021; Ho & Wong, 2001; Jiang & Bajpayee, 2009; Olsen et al., 2020). Similarly, differential recruitment of the two isoforms of β‐arrestin (β‐arrestin 1–2) can translate to distinct functional outcomes, with respect to regulatory and signalling paradigms (Ghosh et al., 2019; Srivastava, Gupta, Gupta, & Shukla, 2015).

In addition, a range of modulatory proteins interact with and change the signalling of receptors, transducers or effectors. For example, receptor activity‐modulating proteins (RAMPs) bind to receptors and can alter their trafficking as well as G protein and/or arrestin binding (Hay & Pioszak, 2016). In the case of the calcitonin and calcitonin receptor‐like receptor, different receptor‐RAMP complexes produce distinct pharmacological responses and are therefore considered as separate receptor subtypes: one calcitonin, two adrenomedullin, and three amylin receptors (Hay, Garelja, Poyner, & Walker, 2018). Similarly, the cannabinoid CB_1_ receptor can bind to cannabinoid receptor interacting protein 1a (CRIP1a), yielding distinct pharmacology (Oliver et al., 2020). GPCRs are also substrates for second messenger‐activated kinases such as the cAMP‐dependent kinase (PKA), PKC, and the casein kinase (CK), with each producing different effects on receptor signalling and trafficking (Bouvier, Leeb‐Lundberg, Benovic, Caron, & Lefkowitz, 1987; Hausdorff et al., 1989; Tobin, Totty, Sterlin, & Nahorski, 1997). Additionally, numerous downstream intracellular effectors modulate pathway responses as scaffolding proteins, for example, kinases and PDZ proteins (Bockaert, Fagni, Dumuis, & Marin, 2004; Kenakin, 2019; Maurice et al., 2011). The regulator of G protein signalling (RGS) proteins selectively modulate G protein subtypes and differentially alter G protein signal strength (Hollinger & Hepler, 2002; Masuho et al., 2020; Neubig & Siderovski, 2002). Furthermore, GRK2 and GRK3 have a RGS homology domain (RH) binding to G_q/11_ to inhibit signalling, and a pleckstrin homology (PH) domain that can bind to G_βγ_ to inhibit its signalling while inducing recruitment of GRK to the receptors (Carman et al., 1999; DebBurman, Ptasienski, Benovic, & Hosey, 1996; Ribas et al., 2007).

**Terminology summary A**

***Transducer***: For the purpose of defining biased signalling initiated by the GPCRs, transducers are defined as proteins that bind directly to an activated receptor to initiate, facilitate or modulate downstream events such as signalling, trafficking or internalization. This includes G proteins, GRKs and arrestins. Some also use ‘primary’ effector to denote a transducer, but this word can be confusing as these proteins typically bind the receptor one after the other.

***Effector***: Signalling protein located downstream in a transducer's pathway.

***Modulator***: Proteins or molecules that do not mediate, but modulate signalling of a receptor, transducer or effectors. Examples include receptor activity‐modulating proteins (RAMPs), guanine nucleotide exchange factors (GEFs), GTPase‐activating proteins (GAPs), regulators of G protein signalling (RGSs), NO, cholesterol and other lipids.

***Second messenger***: Small molecules or ions directly controlled by the effectors. Changes in second messenger homeostasis mediate cellular responses and can serve as a quantifiable measure of GPCR activation. Examples include cAMP and calcium.

***Pathway***: A pathway is named after a transducer protein, or family thereof, that binds to GPCRs. This includes G proteins and their families—that is, G_s_, G_i/o_, G_q/11_, and G_12/13_ which have distinct downstream effectors. It also includes the GPCR kinase (GRK) and arrestin families, which are often but not always engaged following G protein activation.

### 2.2 Ligand bias definition and distinction from system bias

**FIGURE 1:**
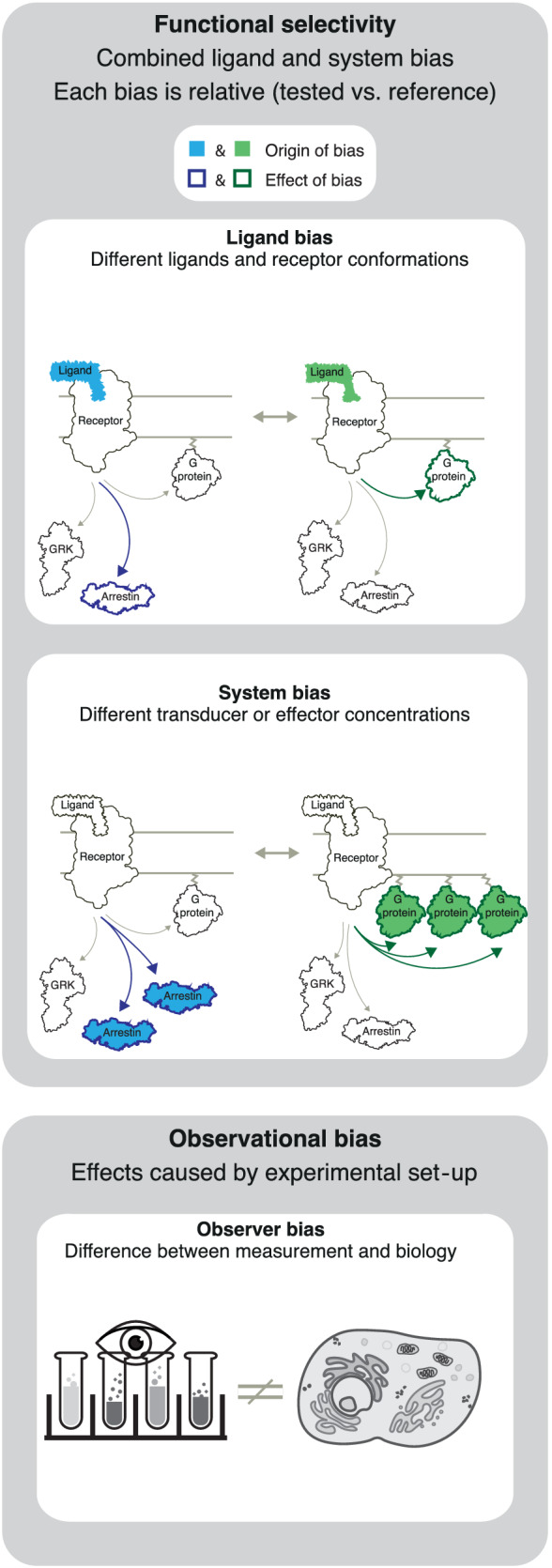
Ligand, system and observational bias. Ligand and system bias together determine the functional selectivity (see terminology subsection for definitions). Each type of bias is measured relative to a reference. Observational bias is not of biological origin, but a consequence of assay sensitivity/non‐linearity and the experimental set‐up

This paper focuses on **ligand‐dependent bias**, that is, cases where a receptor's pathway engagement changes when binding a given ligand (Figure 1). Quantification of bias compares two pathways at a time, typically the pathway with the strongest signalling relative to the most relevant other pathway(s). It also uses a reference ligand and is therefore a comparison of both pathways and ligands (like a quantitative rank order).

In addition to ligand bias, a receptor's response is also determined by **system bias**, which encompasses all non‐ligand molecules involved in signalling. System bias encompasses, for example, the stoichiometry of receptors, transducers, effectors, and modulatory proteins which varies spatially across tissues, cell types, and temporally across physiological states. System bias includes a receptor's G protein or arrestin selectivity. For example, some GPCRs lack the inherent ability to elicit G protein coupling while exhibiting robust arrestin interaction (Meyrath et al., 2020; Pandey et al., 2021; Rajagopal et al., 2010). Furthermore, system bias depends on the different levels of receptor constitutive activity, and intracellular intra‐ or inter‐pathway feedbacks. System bias affects all ligands equally (including the reference ligand), unless the system itself has an influence on ligand concentration (e.g. by conditions where one of two ligands is metabolized).

**Functional selectivity** is the combined effect of ligand and system bias (Stallaert, Christopoulos, & Bouvier, 2011). For example, a GPCR can give rise to different physiological effects after activation by alternative endogenous agonists or for the same ligand in different cells/tissues with differentially expressed signalling components. In drug discovery, achieving functional selectivity by tuning ligand bias on the background of system bias in the tissue of interest provides an opportunity to elicit predominantly the therapeutically beneficial effect of a receptor target.

Biased signalling studies can also suffer from **observational bias**, which is an artificial bias caused by an inappropriate experimental set‐up and therefore not reflecting native biology. An example are time points for data collection that are not relevant for the physiological effect. Another example is if one of the two studied pathways is measured using an assay with insufficient sensitivity or, conversely, has an artificially high level of amplification. Therefore, it is necessary to ensure that at least one assay for each pathway has sufficient sensitivity (preferred) or to increase expression levels of the involved signalling partners (alternative). Moreover, the actual signal plateau may be missed if the signal detection tools saturate prematurely. Observational bias can be avoided by using an independent ‘orthogonal’ assay to validate each pathway.

**Disclaimer:** A ligand may be unbiased (relative to the reference ligand) with respect to two particular pathways but may be biased when additional different pathways are studied or if a different reference ligand is used. Hence, the term ‘biased ligand’ cannot be used as an absolute descriptor for a ligand, but only in the context of receptor, reference ligand, and pathways.

**Terminology summary B**

***Ligand bias***: Ligand‐dependent preferential receptor activation so that one over other transducer pathways in a given cellular system and relative to a reference ligand is induced (see below).

***System bias***: Bias due to differences in the cellular system, including so called ‘tissue bias’. System bias can, for example, arise from differential expression levels of receptors, transducers, effectors, or modulatory proteins.

***Functional selectivity***: Functional selectivity is the observed response combining ligand‐ and system‐bias.

***Observational bias***: An artificial bias introduced by the experimental set‐up. This effect can be minimized, for example, by using equally sensitive assays across pathways.

## 3 CHOOSING A REFERENCE LIGAND

### 3.1 The choice of reference ligand distinguishes benchmark‐, pathway‐, or physiology‐bias

The meaning of ligand bias depends on the choice of reference ligand (Table 1). When the reference ligand is a tool compound, for example, isoprotenerol/isoprenaline for the β_2_‐adrenoceptor) or a drug (e.g. to investigate if an agent in a clinical trial is different from this drug), we refer to this as **ligand benchmark‐bias**, as all that can be concluded is how the ligands differ in bias. In contrast, **ligand pathway‐bias** utilizes the reference ligand with the most balanced signalling across pathways, allowing the researcher to identify ligands for or functionally dissect a specific pathway. Finally, **ligand physiology‐bias** utilizes the principal physiological agonist as the reference ligand and consequently can conclude whether the tested ligands induce ‘natural’ or ‘unnatural’ signalling. Thus, the three types of ligand bias are complementary in that they allow different scientific conclusions to be drawn for the tested ligands.

**TABLE 1:** Types of ligand bias and their meaning by choice of reference ligand

Type of bias	Reference ligand	Meaning (what can be concluded from data)	Disclaimer (meanings/conclusion not supported by data)
Ligand benchmark‐bias	Any ligand, for example, a candidate drug or tool compound. The reference ligand can be arbitrarily chosen, but often has a particular relevance as tool or clinical agent and is therefore selected to benchmark other tested ligands.	Simultaneous comparison across pathways and ligands where the reference ligand can be any ligand of choice.	A biased ligand for which the reference ligand was not selected based on specific signalling pathway qualities has bias only relative to the reference ligand, which in turn can elicit any bias.
Ligand pathway‐bias	Pathway‐balanced ligand	Signalling preferentially via one pathway, as the reference ligand approximates a pathway‐balanced signal.	A pathway‐balanced/unbiased ligand can be physiology‐biased, although it is by definition unbiased in the pathway definition. A balanced ligand in one system may not be ‘balanced’ in another (applies to all types of ligand bias).
Ligand physiology‐bias	Principal endogenous agonist	Signalling differs from the physiological, as the reference ligand represents the endogenous response of the given receptor and system.	An endogenous agonist can be pathway‐biased, although it is by definition unbiased in the physiological definition.

*Note*: The terms ‘ligand pathway‐bias’ and ‘ligand physiology‐bias’ are recommended when researchers wish to attribute a specific function (in addition to just a difference to the reference ligand employed).

As an example, the choice of reference ligand changes the preferred pathway of the κ‐opioid receptor agonist naphthoyl‐β‐naltrexamine (β‐NNTA) (White et al., 2014). Using the endogenous ligand dynorphin‐A as the reference, β‐NNTA has a 4.3‐fold bias for β‐arrestin recruitment over G protein activation. However, when using the plant psychedelic salvinorin A, β‐NNTA displays a 8.9‐fold G protein bias instead. When using an analogue, salvinorin B, β‐NNTA still has a G protein bias but less pronounced, only 2‐fold.

#### 3.1.1 Problems and pitfalls

- Bias cannot be reported without the use of a reference ligand, as a presumed bias may otherwise in fact be due to other differences in, for example, the baseline, window, and sensitivity of assays (‘system bias’ or ‘observational bias’ in Figure 1).
- It cannot be concluded that a tested ligand induces isolated/specific signalling via one pathway unless a balanced ligand is used as reference. In all other cases, the only conclusion is that the tested ligand differs from the reference. This is because the reference ligand may itself have pathway‐bias, making a tested balanced ligand appear biased in comparison.

#### 3.1.2 Recommendations for experimental design

- Choose a reference ligand that can support the claims to be made (Table 1). Use a pathway‐balanced and physiological reference ligand to study pathway‐ and physiology bias, respectively.
- Include multiple reference ligands, thus allowing claims about different types of ligand bias. Doing this will extend the insights from the current study and enable comparison to other studies. This entails, if possible, the inclusion of all types of reference ligand: a highly used tool compound, a drug, a pathway‐balanced ligand, and a physiological ligand. Such ligands are available from the receptor pages of the Guide to Pharmacology database (Harding et al., 2021) and the ligand and drug resources in GPCRdb (Hauser, Attwood, Rask‐Andersen, Schioth, & Gloriam, 2017; Kooistra et al., 2021).
- Measure the reference ligand(s) and the ligands tested for bias, under identical conditions.
- If needed, use separate reference ligands for bias and E_max_. If the reference ligand for bias is not a full agonist, but a partial agonist producing less than a full receptor response (maximum efficacy, E_max_), a separate reference ligand for E_max_ should be used to exploit the full window of receptor response. For the G_s_ pathway, some studies use forskolin, which activates adenylate cyclase directly, rather than a reference ligand to determine the maximal response. It is possible to use different reference ligands for E_max_ in the different pathways, as long as the reference ligand for bias and all ligands tested for bias are normalized relative to the same reference ligand for E_max_. For mathematical models quantifying bias, see Section 6.2.

#### 3.1.3 Reporting recommendations

- Report the reference ligand along with a motivation to why it was chosen. For example, ‘The reference ligand A was chosen, as it (i) is the principal physiological agonist, (ii) has a relatively balanced signalling through pathways P1 and P2 (provide bias plot slope, or (iii) a clinical or tool compound for which enhanced pathway specificity is desired through the introduction of bias’.
- The claims should be restricted to what is supported by the chosen reference ligand (Table 1). Thus, in order to conclude on pathway‐ and physiology‐bias, a balanced and physiological reference ligand, respectively, must be used.
- Define the bias type. Instead of just ‘ligand bias’, we recommend using the specific terms ‘ligand benchmark‐bias’, ‘ligand pathway‐bias’, and ‘ligand physiology‐bias’ when the reference ligand is a drug/probe, balanced and physiological ligand, respectively. Although the person who generated the data may only be interested in one type of bias, the community understands the meaning of bias differently.

**FIGURE 2:**
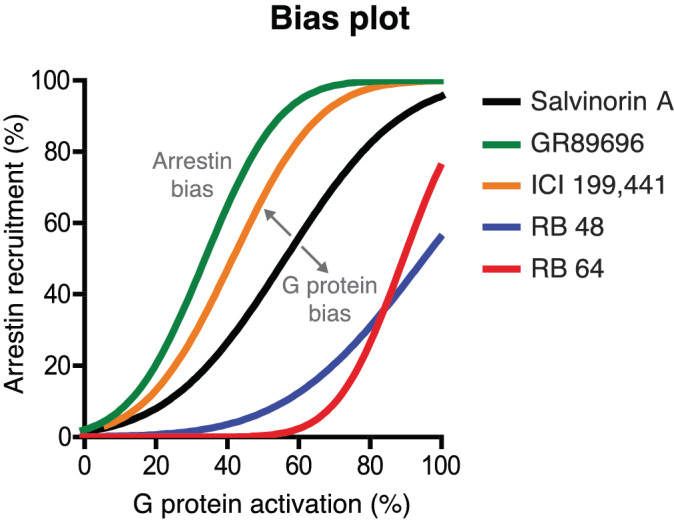
Bias plot of an equimolar comparison of ligand‐induced activities in two pathways. The plot is adapted from White et al., (2014) in which Salvinorin A was chosen as the reference ligand because it has a bias plot slope close to 1. GR89696 and ICI 199,441 are arrestin‐biased and RB 48 and RB 64 are G protein‐biased. The pathway percent activation could, for example, use ΔΔLog (E_max_/EC_50_) or (ΔΔLog(τ/K_A_) values (see Section 6.2 and Box 1)

#### 3.1.4 Disclaimer

**Assay/system dependence:** If the system or assays change, the same ligands, receptor, and pathways can display a different bias. This is because a reference ligand does not automatically equalise an imbalance in pathways but merely moves the baseline. Imbalance in pathways is instead predominantly determined by the choice of systems and assays, which should be as similar as possible.

**Terminology summary C**

**Biased ligand:** Ligand preferentially activating one receptor transducer pathway in a given cellular system and relative to a reference ligand. Ligand bias is a property of not just a ligand, but of a ligand, pathway pair and receptor in combination, and only valid within the specific system that is investigated. Therefore, the term ‘biased ligand’ should only be used if explicitly defined, and not be construed to represent a ligand‐only property. A recommended definition is included in Section 8.1, which provides one‐sentence and table templates for reporting.

**Reference ligand for bias:** The ligand that is, by definition, unbiased. The bias of any other tested ligands is quantified relative to this reference.

**Reference ligand for E**_**max**_: A separate reference ligand for the full receptor response (maximum efficacy, E_max_). This allows exploiting the full window of receptor response even when the reference ligand for bias is a weak partial agonist.

**Unbiased ligand:** A ligand that stimulates pathways in a manner indistinguishable from the reference ligand.

### 3.2 Ligand pathway‐bias (uses a pathway‐balanced reference ligand)

Ligand pathway‐bias is the most relevant type of ligand bias when seeking to discover a drug with functional selectivity, as it describes predominant signalling via one pathway. A pathway‐balanced reference ligand (typically a surrogate, but can also be an endogenous ligand) has near equal signalling through compared pathways, meaning that if a tested ligand is biased, it preferentially activates one pathway over the other (Table 1).

#### 3.2.1 Problems and pitfalls

**Lacking reference ligands:** When no balanced ligand is available, bias can still be measured but not interpreted as pathway‐bias. In these cases, it can be useful to visualize the relative pathway preferences of the most interesting tested ligands in a bias plot (Figure 2).

#### 3.2.2 Recommendations for experimental design

- Determine a pathway‐balanced reference ligand in a bias plot. A balanced ligand can be defined based on a bias plot, which is an equimolar comparison of induced pathway activities (Figure 2). A balanced (least biased) ligand has a slope of (close to) 1.

#### 3.2.3 Reporting recommendations

- Document the pathway‐balance of the reference ligand. For example, present a bias plot (Figure 2) or reference a previous supporting study, if available.

#### 3.2.4 Disclaimer

**Context specific bias:** Many researchers argue, rightly so, that a ligand referred to as balanced will not be balanced in another system or assay. Therefore, any claim of a ligand being balanced and tested ligands exhibiting pathway‐bias is always system‐specific (which is true for all ligand bias studies). See also the Section 5.1.

**Terminology summary D**

**Balanced ligand:** Has indistinguishable or very similar signalling through compared pathways (Figure 2).

**Ligand pathway‐bias:** Ligand bias that is measured relative to a balanced reference ligand and therefore has the meaning that signalling is predominant via one pathway.

### 3.3 Ligand physiology‐bias (uses the principal endogenous ligand as reference)

When using an endogenous ligand as a reference ligand, the statement that a tested ligand is biased carries the meaning that its signalling differs from the physiological one (Table 1). This is valuable, for example, when seeking to map the relationships between pathways and physiological effects. Whether this entails a response through a single pathway or not will therefore depend on the extent to which the endogenous ligand engages different transducer pathways or not, in the first place.

#### 3.3.1 Problems and pitfalls

- The same receptor often has multiple endogenous agonists and depending on which one is used as the reference for a ligand physiology‐bias study, the measured bias may change substantially.
- A secondary endogenous agonist can be biased relative to the principal endogenous agonist.
- Ligand physiology‐bias cannot be determined for ‘orphan’ GPCRs, as they have no known endogenous ligand.
- Endogenous ligands with low efficacy cannot be quantified for bias (see Section 7.1).

#### 3.3.2 Recommendations for experimental design

- Use the principal endogenous agonist as reference ligand for studies of how a tested ligand differs from the natural signalling, that is, ligand physiology‐bias. The principal endogenous agonist is that which is most abundant and potent in the receptor's most physiologically relevant tissue. Principal and secondary endogenous agonists of GPCRs are curated by experts coordinated by NC‐IUPHAR and available in the receptor pages of the Guide to Pharmacology database (Harding et al., 2021). In cases where it is not clear which endogenous agonist represents the principal agonist, we recommend using the endogenous agonist with the highest potency (efficacy is not annotated in this database) or efficacy in the given investigators' assay and system.

**Terminology summary E**

**G protein selectivity**: The profile of one or more G proteins that a receptor engages, typically when activated by the principal endogenous ligand reference. This may be a subset of additional G proteins that the receptor can engage when bound to other agonists. The term ‘natural bias’ is self‐contradictory and should not be used.

**Ligand physiology‐bias:** Ligand bias relative to a receptor's principal endogenous agonist, which therefore bears the meaning that signalling differs from the physiological.

### 3.4 Ligand pathway‐preference (uses no reference ligand, and is not ligand bias)

Without a reference ligand, a study cannot conclude on ligand bias. Investigation of a ligand's differential activity across pathways (e.g. pathway ΔLog (E_max_/EC_50_) or ΔLog(τ/K_A_) values), but not relative to a reference ligand, is here instead referred to as **pathway‐preference**. Comparing pathway‐preferences of a number of tested ligands can be a useful way to select a reference ligand for subsequent calculation of ligand bias. It may also be a way to rank order ligands without defining a fixed reference point.

#### 3.4.1 Problems and pitfalls

- Studies cannot claim ligand bias without a reference ligand.

#### 3.4.2 Recommendations for experimental design

- Compare pathway ΔLog (E_max_/EC_50_) or ΔLog(τ/K_A_) values, not only fold potencies, as differing efficacies can substantially influence the relationships (Box 1).
- Use the same or near‐identical systems and assays, as without a reference ligand any system bias will have a larger influence.

**Terminology summary F**

**Pathway‐preference:** A ligand's differential activity across pathways (e.g. pathway ΔLog (E_max_/EC_50_) values), but without comparison to a reference ligand.

## 4 MEASURING AT THE TRANSDUCER OR DOWNSTREAM

### 4.1 Ligand bias measured at the transducer level

Measuring receptor‐binding transducers (G proteins, GRKs, or arrestins) avoids downstream signal amplification. This can make experiments more comparable across pathways than measuring downstream effectors or second messengers.

#### 4.1.1 Problems and pitfalls

**FIGURE 3:**
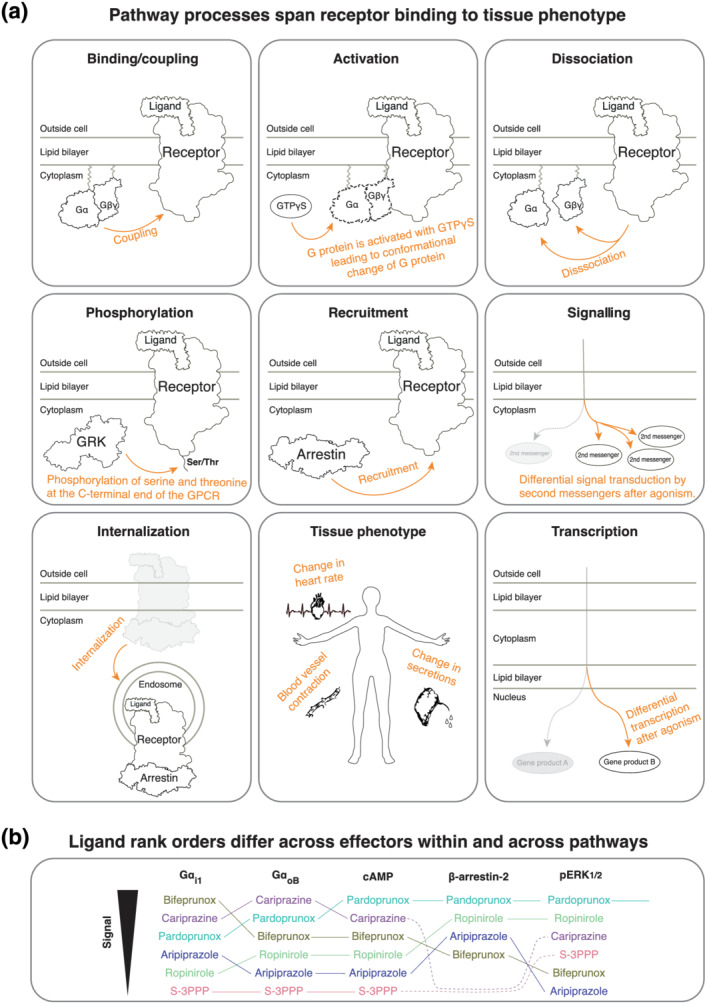
Recommendations to describe the measured pathway process and molecules. (a) Terms to describe the measured pathway process. Also see reviews describing the translation across these levels (Luttrell, Maudsley, & Gesty‐Palmer, 2018), assays (Smith, Lefkowitz, & Rajagopal, 2018) and animal models (Bradley & Tobin, 2016). (b) Data from (Klein Herenbrink et al., 2016) show the relative bias of dopamine D_2_ receptor agonists for five pathways (all with dopamine as the reference ligand). The relative order of the ligands changes depending on the measured molecules, even across those participating in the same pathway. This emphasizes that bias should be measured at similar pathway depths (comparably proximal/distal to the receptor), each of which should be defined with respect to the measured entities

- If a single representative transducer is used, it may not be representative for the signalling behaviour of the entire transducer family (e.g. G_q_, G_11_, G_14_, and G_15_ in the G_q/11_ family). Differential activation or recruitment of individual transducer family members has been shown both for G protein families (Avet et al., 2021; Inoue et al., 2019; Masuho et al., 2015; Namkung et al., 2018; Olsen et al., 2020) and the arrestin family (Avet et al., 2021; Srivastava, Gupta, Gupta, & Shukla, 2015).
- Different cellular systems can express transducers differentially. Hence, even measurements of transducer‐levels, albeit devoid of signalling amplification, can differ across cells and tissues.
- Assays are often only referred to by their detection method, for example, ‘BRET assay’. This is insufficient, as the same detection technique can be used to measure fundamentally different molecules and processes, such as GPCR‐G protein binding/coupling or G_α_‐G_βγ_ dissociation (Figure 3a).

#### 4.1.2 Recommendations for experimental design

- Use recent assays consistently profiling G proteins and arrestins (also with GRKs) (Avet et al., 2021; Olsen et al., 2020) (The assays in Olsen et al. were optimized from Gales et al. 2006; Lukasheva et al., 2020).
- If possible, test all transducers for each investigated transducer family because it cannot be assumed that one transducer is representative for the signal mediated by the entire family.
- If selecting representative transducer subtypes, use the most relevant. If not all transducer subtypes within a given transducer family can be tested, a representative could be selected based on the transducer expression levels in the tissue most relevant for the study. If this is not known, another rationale could be to select the transducer subtype with the highest activation or recruitment. Data to support such a selection have come from, for example, Avet et al. (2021), Inoue et al. (2019) and Olsen et al. (2020) and have been integrated in GproteinDb (Pandy‐Szekeres et al., 2022) and ArrestinDb (https://arrestindb.org).

#### 4.1.3 Reporting recommendations

- Modifications of receptors, transducers, or effectors must be clearly defined (e.g. tags, mutations and chimeras).

### 4.2 Ligand bias measured downstream of distinct transducers

Whereas ligand bias is often grouped into pathways represented by the primary receptor‐binding transducers (G proteins, GRKs, and arrestins), most studies measure downstream effector proteins or second messenger molecules (Figure 3a). Unlike measurements at the transducer level (previous section), this collates the signalling of all subtypes of a transducer family and may correlate better to the tissue‐level or systemic response. However, they may suffer from pathway convergence or crosstalk (see Section 4.3).

#### 4.2.1 Problems and pitfalls

- If two pathways are measured at different depths, one close to the receptor and another further downstream, this can lead to a higher signal amplification for the latter pathway. This observation bias can cause apparent ligand bias. For example, arrestin is nearly always measured as recruitment or binding to receptors because there is no consensus on a downstream effector or second messenger. Conversely, G protein pathways are often measured at the downstream level, typically as a secondary messenger.
- Ligand bias relationships may differ depending on which of a pathway's downstream molecules are measured (Figure 3b).

#### 4.2.2 Recommendations for experimental design

- Minimize differential signal amplification by measuring pathways at similar depth (estimated based on the number of upstream effectors, Figure 3a). Where the pathway readouts differ in depth, special consideration should be given to potential amplification effects.

#### 4.2.3 Reporting recommendations

- Report measured processes. The same molecule can be involved in several steps of the signalling process. For example, G proteins can be measured for receptor binding, activation, nucleotide exchange, or subunit dissociation. A non‐exhaustive list of terms to distinguish such signalling processes is visualized in Figure 3a and tabulated in Table 2 along with example assay principles.
- Report the measured molecules. To provide clarity on what has been measured, we recommend that the pathways are defined not only for the upstream GPCR‐binding transducer but also for the downstream measured molecule or molecule pair (Table 3).

**TABLE 2:** Suggested terms to define the pathway processes at different levels being compared for bias

Term	Examples of assay principles
Accumulation	Ca^2+^ or cAMP accumulation
Binding/coupling	Proximity
Activation	Receptor conformation and G protein activation, for example, GTPase activity, GTPγS binding, or recruitment of downstream effectors.
Dissociation	G protein dissociation/rearrangement
Phosphorylation	Incorporation of ^32^P in a specific protein. Two‐dimensional phosphopeptide mapping after orthophosphate labelling, MS‐based identification of phosphorylation sites, Phosphosite‐specific antibody assays or arrays
Recruitment	Subcellular redistribution
Signalling	Downstream cellular second messenger levels
Internalization	ELISA, flow cytometry, diffusion‐enhanced resonance energy transfer (DERET), enhanced bystander BRET (ebBRET), (confocal) microscopy, antibody‐feeding experiments, real‐time internalization by SNAP‐tagged receptors
Tissue phenotype	Blood vessel contraction, cell migration, hormone secretion, heart rate, force of contraction, glandular secretion.
Transcription	Differential gene arrays after agonism, qPCR, and gene reporter assay.

*Note*: For specific assays implementing the tabulated assay principles, we refer readers to Bohn & McDonald (2010); Bohn, Zhou & Ho (2015); Masuho, Martemyanov & Lambert (2015); Perry‐Hauser, Asher, Hauge Pedersen & Javitch (2021); Wright & Bouvier (2021).

**TABLE 3:** Experimental parameters critical to the unambiguous description of ligand bias

Parameter	Pathway 1	Pathway 2	Pathway 3
Transducer (‘pathway’)	β‐Arrestin 2	G_i_	G_q_
Cell line	CHO	CHO	CHO
Receptor expression levels (fmol·mg^−1^ protein)	300	300	300
Time point for data collection (min) (for time‐resolved, a span)	15–60	30	30
Temperature (°C)	20	20	20
Reference ligand for bias	CP55,940	CP55,940	CP55,940
Reference ligand for E_max_	CP55,940	CP55,940	CP55,940
Measured process	Recruitment	Accumulation	Accumulation
Measured molecule 1^a^	CB_1_ receptor	cAMP	Inositol 1‐phosphate
Measured molecule 2^a^ (if any)	β‐Arrestin 2	‐	‐
Signal detection technique	FRET	HTRF	HTRF

*Note*: Table entries are examples only.

^a^ A measured molecule is a GPCR, transducer, effector or second messenger.

### 4.3 Ligand bias measured downstream of converged transducer pathways

#### 4.3.1 Problems and pitfalls

- Several transducer pathways intercept or converge downstream. If measuring at or downstream of such pathway nodes, the signal cannot be mapped back to a specific transducer. For example, ERK proteins can be activated by all four G protein families (Jain, Watson, Vasudevan, & Saini, 2018), and this process is shaped in space and time by arrestins and GRKs (Eichel, Jullie, & von Zastrow, 2016; Gurevich & Gurevich, 2018; Gutkind & Kostenis, 2018; Luttrell et al., 2018; Wehbi et al., 2013).

#### 4.3.2 Recommendations for experimental design

- Avoid measuring downstream of converged pathways, unless the purpose is to compare two ‘deep’ signals close to the cellular endpoints without a need for relative transducer contributions.
- If possible, dissect upstream transducer contributions using other assays.

#### 4.3.3 Reporting recommendations

- When pathways are truly inseparable and their contributions cannot be dissected using upstream assays, the bias may be described as a type of ‘effector bias’ (instead of pathway‐bias) accounting for the net pathway contributions.
- Interpret findings in light of their relative strength for the given receptor and ligand. For example, calcium, PKC, or DAG measurements should not be exclusively equated to G_q_ activation. Indeed, different receptor transducers, including G_βγ_ released from G_αi_ can also lead to the generation of these second messengers and activation of this kinase (Dorn, Oswald, McCluskey, Kuhel, & Liggett, 1997).

## 5 CONSIDERING CELLULAR SYSTEM, KINETICS AND SPATIAL BIAS

### 5.1 Bias may not translate across in vitro, physiological, and therapeutic systems

#### 5.1.1 Problems and pitfalls

- Most in vitro pharmacology experiments are performed in systems (e.g. HEK cells) that may differ substantially from a physiologically or therapeutically relevant system. Given the cell phenotype‐ and physiological cell state‐dependence of bias, the measured bias incurs the risk of not translating to the expected physiological or therapeutic effect in human (Bohn, Zhou, & Ho, 2015).
- The use of recombinant and/or overexpressed receptor, transducer, or effector proteins may not reflect the ligand bias in a native system. Insofar as ligand bias quantification can be carried out in a recombinant system, it serves to identify bias as a property of the molecule (not the system) and still serves as an identifier for molecular mechanisms to be elucidated further in natural systems.
- Some signalling pathways are difficult to evaluate in tissues (i.e. arrestin recruitment) as many assays require genetically modified tags to be applied to receptors and/or effectors. If modifications are made to proteins in endogenous systems, those modifications must be clearly indicated, and interpretations should consider the possible effects of overexpression and modification on the system.

#### 5.1.2 Recommendations for experimental design

- Where possible, use primary and/or disease‐specific cells and evaluate potential system bias.
- Where possible, validate the effect in a model organism using an appropriate model of efficacy and/or genetic engineering to confirm target and pathway specificity.

#### 5.1.3 Reporting recommendations

- Where a non‐native system has been used, conclusions should be stated carefully such that a ligand inducing bias in this system may or may not have a beneficial effect in a therapeutically relevant system in vivo.

### 5.2 Kinetics and choosing measurement time points

#### 5.2.1 Problems and pitfalls

- Ligand bias often depends on the time points of data collection. For instance, comparing non‐equilibrium readings with equilibrium readings due to different binding kinetics or type of biological responses (ion flux vs. reporter gene) can be a major confounding factor (Klein Herenbrink et al., 2016).
- The physiologically most relevant time point is often the same across pathways but can differ if the signalling processes (Figure 3a) occur over different time scales.
- A ligand's effect is time‐dependent, as signalling efficacy changes (e.g. due to desensitization), which can be pathway‐dependent (Stout, Clarke, & Berg, 2002).

#### 5.2.2 Recommendations for experimental design

- When possible, complete time courses and endpoint measurements should be made. These could be quantified by onset kinetics, for example, time constant tau (τ) or time to reach half maximal response amplitude (Hoare, Pierre, Moya, & Larson, 2018).
- Single time points should be the physiologically most relevant or measure the maximum effect (peak).

#### 5.2.3 Reporting recommendations

- Report the chosen time points and the reason(s) why they were chosen.
- Provide data on the complete time course, if available.

**Further reading:** See Lane, May, Parton, Sexton, & Christopoulos (2017).

**Terminology summary G**

***Temporal effect***: The effect influencing a measured response due to the choice of time point at which a response is recorded. Apparent bias can occur simply because the kinetics of the response is different between two pathways for two different ligands. These time effects include effects of the binding kinetics (k_on_ and k_off_), time course of the biological response measured, and time domain of the assay itself (e.g. reporter gene vs. ion flux).

### 5.3 Spatial bias: Differing signalling efficacies across cellular compartments

#### 5.3.1 Problems and pitfalls

GPCRs signal from a wide range of cellular compartments other than the plasma membrane, including endosomes, the Golgi and the nucleus (Crilly & Puthenveedu, 2021; Jong, Harmon, & O'Malley, 2019). This signalling can be modulated by different transducers, such as β‐arrestins (Luttrell et al., 2001), G_α_ subunits (Feinstein et al., 2011; Irannejad et al., 2017), and G_βγ_ subunits (Masuho, Skamangas, Muntean, & Martemyanov, 2021). With this has come the realization that signalling involving the same transducer from different compartments can have distinct outcomes; for example, cAMP evolution from endosomes but not the plasma membrane promotes gene transcription (Tsvetanova & von Zastrow, 2014). This phenomenon of GPCR signalling through the same transducer in different locations producing distinct signalling responses has been referred to as ‘spatial’ or ‘location bias’. Furthermore, spatial bias extends to ligand‐biased trafficking. For example, ligands differentially regulate receptor recycling following endocytosis (Namkung et al., 2016).

#### 5.3.2 Reporting recommendations

- Report the biosensors and tags used for monitoring compartment‐specific signalling.
- Report the cell types used in assays, as some cell types lack transporters, such as OCT3/SLC22A3, required for the trafficking of hydrophilic small‐molecule ligands that cannot cross the plasma membrane (Irannejad et al., 2017).
- Report ligands with altered characteristics, for example, permeability, through chemical modification (Jensen et al., 2017).

**Terminology summary H**

***Spatial/location bias***: The observation of biased GPCR signalling through the same transducer in different locations that results in distinct signalling responses. This GPCR signal may originate from different compartments or may be from the same compartment but then result in the trafficking of transducers to different compartments (Masuho, Skamangas, Muntean, & Martemyanov, 2021).

## 6 HOW TO QUANTIFY LIGAND BIAS

### 6.1 Many models exist to quantify ligand bias

The classical theory of receptor action (Furchott, 1966; Stephenson, 1956) aims to separate ligand‐ and system‐dependent parameters and forms a basis of strategies to quantify ligand bias. Many methods to quantify ligand bias are based on the null principle of equi‐effective concentrations of agonist producing equal responses, first described by Barlow, Scott, and Stephenson (1967) and theoretically justified within the framework of the Black‐Leff operational model (Black & Leff, 1983).

However, there are still different views on how to best quantify bias and strategies for quantification are still being developed and improved. Hence, there are numerous models that could be used, as long as pathways are measured with consistent pharmacological parameters, and in similar systems and assays with corresponding levels of downstream processes and kinetics (see above). Of note, even with the most detailed models, it is not possible to directly compare quantitative estimates of ligand bias across different studies.

#### 6.1.1 Problems and pitfalls

- Quantification of ligand bias with different methods can in some cases lead to different conclusions on the biased/unbiased nature of a ligand (and system) (Onaran et al., 2017; Rajagopal et al., 2011) or to a different relative bias rank order of ligands.
- No model can provide an absolute bias value of a given ligand at a given receptor. Only bias values relative to a reference ligand are accessible with current quantification techniques.

#### 6.1.2 Recommendations for experimental design

- It is not possible to recommend a single, best‐practice, quantification method due to the lack of consensus in the community, with new or refined methods still being proposed.
- Results are more definitive when bias is quantified using multiple models.
- Irrespective of the model, error propagation and statistics must be handled appropriately.

**Further reading:** We may refer readers to Table 4 in Kenakin (2019) for an overview of methods to quantify ligand bias, and Onaran and Costa (2021) for a critical review of the detailed principles, on which specific implementations are based.

### 6.2 A minimal and a refined model to calculate a ligand bias factor

The two most commonly used models to quantify ligand bias are relative‐relative Log (E_max_/EC_50_) (Ehlert, 2008) and relative‐relative Log(τ/K_A_) (Box 1, Kenakin, 2015b). ΔΔLog (E_max_/EC_50_) is easy to use, as it only requires E_max_ and EC_50_ values for which the log ratio is first compared for two ligands and subsequently for two pathways (Box 1). For ligands with concentration–response curve slope factors (Hill coefficients) of 1, the ΔΔLog (E_max_/EC_50_) values are identical to Log(τ/K_A_) values. For other ligands, ΔΔLog(τ/K_A_) values are more correct, as they account for receptor density and coupling within a system. Like E_max_/EC_50_, τ/K_A_ can be calculated from a concentration–response curve, but it requires a software, such as GraphPad Prism, to solve the Black/Leff model and special care for full agonists which can have ambiguous fits (Box 1). Both ΔΔLog (E_max_/EC_50_) and ΔΔLog(τ/K_A_) can be expressed as a bias factor, which is the antilog. As the bias factor is on a logarithmic scale, a difference of 1 corresponds to a 10‐fold activation difference. A template (MS Excel file) to calculate bias factors is available at https://files.gpcrdb.org/bias_guidelines/Template_to_calculate_ligand_bias_factors.xlsx.

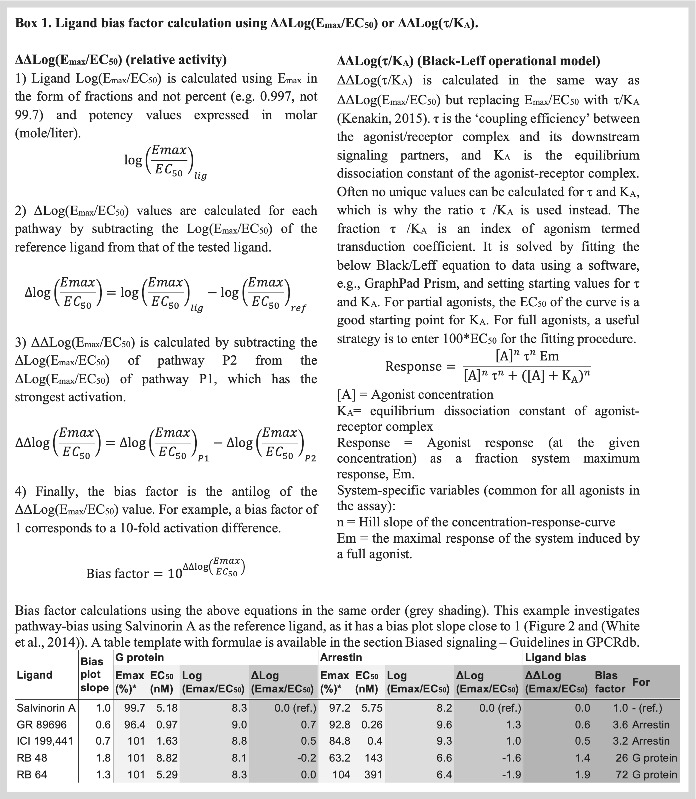

#### 6.2.1 Recommendations for experimental design

- ΔΔLog (E_max_/EC_50_) should not be used when ligand concentration–response curve slope factors (Hill coefficients) are not close to 1, as this quantification is theoretically justifiable only when the slope factor is close to 1.
- ΔΔLog(τ/K_A_) values are preferred over ΔΔLog (E_max_/EC_50_) when ligand concentration–response curve slope factors (Hill coefficients) are not close to 1.
- Confirm that the calculated bias factors are consistent with a bias plot (Figure 2). There is unlikely to be bias in a system if it cannot be visualized in a bias plot. Although a bias plot only provides a qualitative assessment of bias, it does not have the weakness of relying on fit parameters that can have artificially low errors from fitting routines.

#### 6.2.2 Reporting recommendations

- Report ligand E_max_, EC_50_, and τ/K_A_ values and system maximum response, Em. We recommend authors to tabulate the underlying quantitative values of the pharmacological parameters. Reporting these values will improve clarity, increase transparency and future‐proof cross‐study comparisons. It also allows the calculation and presentation of multiple models of bias or to change the choice of reference ligand to facilitate comparison across studies.
- Report the ligand concentration–response curve slope factors (Hill coefficients). This information is critical, especially when ΔΔLog (E_max_/EC_50_) is used to assess bias, as slope factors deviating from 1 indicate the extent to which system bias contributes to the calculated bias factors.
- Report a bias plot for biased ligands. This will serve to validate quantified ligand bias in a qualitative and visualized manner.

**Further reading:** For further guides on how to calculate ΔΔLog (E_max_/EC_50_) and ΔΔLog(τ/K_A_) values, we refer the reader to (Kenakin, 2015b; Kenakin & Christopoulos, 2013; Rajagopal et al., 2011).

**Caution:** A large degree of caution is advisable for describing ligands with only weak bias (low bias factor) or absolute efficacy (low E_max_), as these compounds are more likely to produce system‐dependent bias effects (see Section 7.1). Such agonists are therefore more likely to be spuriously identified as biased, as both methods outlined above rely on best‐fit parameters. Weak partial agonists will result in relatively poor fits (but still with excellent R^2^) with E_max_/EC_50_ or τ/K_A_ values that grossly underestimate the errors of the derived bias factors. One can use a bias plot to confirm non‐quantitatively that bias exists between two compounds, but one should never rely on bias factors alone.

### 6.3 Comparing ligand bias across studies and systems (use of rank orders)

#### 6.3.1 Problems and pitfalls

Bias values obtained from different experimental systems are not comparable on a quantitative level. For example, a bias value above 2.0 in one system may be below 2.0 for the same pathways when studied in another system differing by, for example, cell line, measured molecules or process (Figure 3). Achieving a more consistent assessment of which ligand is the most biased towards a given pathway is important to identify functionally selective probes that can be used to dissect a distinct effect. This provides information about which pathways should be targeted or avoided in the design of drugs with higher efficacy and fewer side effects.

#### 6.3.2 Recommendations for experimental design

- We recommend using ligand rank orders of bias factors (rather than quantitative bias values) for comparisons of ligand bias across studies using different experimental systems. Only the pathways studied in both studies/systems can be compared, and the pathway ranks must be identical (i.e. the strongest, second strongest, etc. pathway).

**Disclaimer:** The relative ligand bias rank orders may also differ across systems (Figure 3b). However, they differ less than detailed quantitative values.

## 7 SPECIAL RECOMMENDATIONS FOR ‘TRICKY’ LIGANDS

### 7.1 Low efficacy agonists

Ligand bias quantification should be considered together with the quantified strength of intrinsic efficacy of ligands in the relevant signalling pathways. This is because organ sensitivity and relative intrinsic efficacy differences have a large influence on a low‐efficacy (for some pathways) ligand, which may appear to be very functionally selective in some organs. However, a signal may ‘appear’ in a more sensitive organ, or in the same organ at a different physiological state.

#### 7.1.1 Problems and pitfalls

- Too low efficacy in one pathway will result in bias factors with very large errors.
- The response may be stronger in more sensitive tissues or assays.

#### 7.1.2 Recommendations for experimental design

- Use ligand pathway rank orders. To avoid misleading conclusions based on low efficacy, one should use rank orders, that is, normalizing within the transducers for each ligand without referencing to other ligands. Only a transducer/pathway rank order change would constitute biased signalling. For example, if ligands A and B share rank order G_s_ > G_q_ >> arrestin they are not biased relative to each other. However, if a third ligand C differs in its rank order, for example, arrestin > G_q_ >> G_s_, it is biased relative to A and B.
- If quantifying bias, use another reference agonist for E_max_ (see Section 3.1.2).

**Caution:** Lack of response in a low‐efficacy pathway does not preclude the antagonism of the natural agonist for that pathway. Thus, a physiological bias can be produced by a synthetic ligand's combined own response (e.g. full agonism in pathway 1) and blockade of the endogenous response (e.g. partial agonist in pathway 2). The outcome of this consideration is that, although ligand physiology‐bias or pathway‐bias may not be concluded for a low‐efficacy compound, it could still result in functional selectivity that may have physiological importance in some tissues in which a low efficacy compound may simply not be efficacious enough to activate all the pathways. Such a situation would therefore result in a different physiological outcome from that of a compound able to activate all these pathways in the same tissue.

### 7.2 Inverse agonists

Inverse agonists inhibiting the non‐ligand‐dependent constitutive activity of a receptor may, as biased agonists do, act differentially on pathways by stabilizing distinct receptor conformations. This is only evident for receptors with constitutive activity in the absence of an agonist. The minimum condition needed to quantify bias would be concentration–response curves in two pathways and this condition can be met for an inverse agonist ligand. For receptors with a high constitutive activity, biased inverse agonism could be valuable to fundamentally understand signalling and to exploit this knowledge therapeutically.

#### 7.2.1 Recommendations for experimental design

- Use another inverse agonist as the reference ligand. When a ligand acts as an inverse agonist in two pathways compared for bias, a bias factor can, in theory, be calculated in the same way as for agonists, but using an inverse agonist reference. As most endogenous ligands are agonists, this typically precludes quantification of ligand physiology‐bias (exceptions exist, for example, agouti is a rare example of an endogenous inverse agonist).

**Further reading:** Methods to quantify bias for inverse agonists are beyond the scope of this paper, as they involve differences in agonist‐mediated and constitutive activity‐mediated efficacy (Ehlert, Suga, & Griffin, 2011). It is known that constitutively active receptors themselves possess an efficacy that can be different from agonist‐mediated efficacy. This is manifested as the phenomenon of protean agonism whereby a low efficacy partial agonist demonstrates positive agonism in quiescent systems and inverse agonism in constitutively active systems (Chidiac, Nouet, & Bouvier, 1996; Kenakin, 1997). This is because the agonist‐mediated active state is of lower efficacy than the constitutively active state. Such phenomena must be considered to ascribe an efficacy to an inverse agonist.

### 7.3 Agonist and antagonist across pathways (‘modality bias’)

#### 7.3.1 Problems and pitfalls

When agonism and no agonism (neutral antagonism or inverse agonism), respectively, are observed in two pathways compared, it is not possibly to quantify bias using the above models. This is because calculation of a quantitated bias factor requires two concentration–response curves with the same modality (agonism or inverse agonism).

#### 7.3.2 Reporting recommendations

- Ligand bias with opposite modalities across pathways can be described as a non‐quantitative term, ‘modality bias’. In this case, there is no need to quantify bias to claim bias.
- Alternatively, it can be approximated by measuring an affinity to limit bias or describe it in a ‘bias is larger than’ relationship (Kenakin, 2015a; Stahl, Ehlert, & Bohn, 2019; Stahl, Zhou, Ehlert, & Bohn, 2015). Specifically, the affinity (determined from functional antagonism) is used to determine receptor occupancy and a very low level of efficacy is assumed to generate a simulated curve (i.e. maximal response of 5%) which is then used to calculate bias. This yields the lowest possible bias (it could be greater than this if the efficacy is lower than the assumed one giving 5% maximal response).

**Caution:** Although a very low ligand efficacy cannot be detected in one system, it may be detected in another functional system with higher sensitivity. Hence, the statement should be understood as a practical qualification, in the sense that the efficacy of the ligand is close to zero within the detection limits of the given system. That is, some partial agonists may appear to be neutral antagonists.

**Terminology summary I**

***Modality bias***: Ligand with efficacy in only one of the compared pathways (neutral antagonist or inverse agonist in others). As for all biased ligands, this refers to a particular system and in another more sensitive system, there may be efficacy at several compared pathways allowing the determination of concentration–response curves. We discourage the use of the previous terms ‘full bias’ or ‘perfect bias’, as they can misleadingly give the impression that the ligand always has full efficacy in the preferred pathway. In fact, a ligand with modality bias may give a weaker activation than other agonists for which bias can be quantified (as they induce agonism in at least two pathways).

### 7.4 Allosteric modulators affecting ligand bias

Pure allosteric modulators will modulate the orthosteric ligand binding and/or efficacy, whereas ago‐allosteric modulators also induce signalling of their own. In all cases, the functional outcome can vary depending on the orthosteric ligands.

#### 7.4.1 Problems and pitfalls

No concentration–response curves can be measured for an allosteric ligand on its own; thus, bias cannot be attributed to it individually but must be defined together with the orthosteric ligand.

#### 7.4.2 Recommendations for experimental design

- Quantification of allosteric modulator bias should make use of an extended operational model of allosterism, which was originally described in Leach, Sexton, and Christopoulos (2007) and further extended in Slosky, Caron, and Barak (2021).

#### 7.4.3 Reporting recommendations

- Report both the allosteric modulator and orthosteric ligand.

**Further reading:** For further reading, including examples of biased allosteric modulators see Slosky, Caron, and Barak (2021).

## 8 PUBLICATION AND DATABASE DEPOSITION

### 8.1 Unambiguous description of ligand bias

All the above information is necessary to interpret ligand bias correctly and to separate it from system bias (Figure 1). Hence, the mention of a ‘biased’ or ‘unbiased’ ligand as short terms are only meaningful after the *prior* definition of this information.

#### 8.1.1 Problems and pitfalls

Ligand bias will not be reproducible and understood consistently without sufficient information about the ligands, pathways and systems. Such ambiguity hampers consensus and advances in the field.

#### 8.1.2 Reporting recommendation

- Define the ligand evoking bias and its system in a clear sentence. This should be at first mention in a manuscript/text. For example, *ligand L displays [recruitment/signalling …] bias towards pathway P1 at time point TP1 over pathway P2 at time point TP2 and relative to reference ligand A at receptor R in cell line C*.
- Tabulate the experimental details required for unambiguous description (Table 3 can be used as a template).
- Deposit biased ligands in a database. GPCRdb offers authors the ability to submit biased ligands, compliant with the reporting recommendations brought forward above. The submission is done via a standardized Excel file detailing all necessary information (https://docs.gpcrdb.org/BiasedSignaling.html#data_submission).

## 9 CONCLUSION AND OUTLOOK

Adopting the guidelines presented here will facilitate consensus and advances in the GPCR field through increased quality, reproducibility and clarity of ligand‐induced bias. More precise experiments will improve individual studies and make them more useful for others, as data become more comparable. Furthermore, the unambiguous and structured reporting of bias data will be directly reflected in more relevant databases and literature reviews.

To unlock the rational targeting of GPCRs by exploiting ligand bias to achieve functional selectivity, we must first map individual or combined pathways to their distinct effects. This requires identification of many more probes inducing bias, pharmacological assays, animal models, and so on. This represents a tremendous challenge, but one with extraordinary potential. The recommendations herein can contribute to this massive endeavour by better description of such probes and effects. We have deliberately left the door open to include additional intracellular transducers, effectors, or modulators involved in GPCR signalling, and our definitions should therefore be future‐proofed, in the sense that they can be applied also to signalling proteins that have yet to be discovered.

Ligand‐dependent biased function is also being investigated for other protein classes, for example, receptor TKs (Karl, Paul, Pasquale, & Hristova, 2020), nuclear receptors (Heidari et al., 2019), monoamine transporters (Hasenhuetl, Bhat, Freissmuth, & Sandtner, 2019), Toll‐like receptors (unpublished), and cytochrome P450 enzymes (Jensen et al., 2021). Many of the recommendations brought forward in this paper are also applicable to these protein families, which would in time benefit from publication of additional dedicated guidelines.

### 9.1 Nomenclature of targets and ligands

Key protein targets and ligands in this article are hyperlinked to corresponding entries in https://www.guidetopharmacology.org, and are permanently archived in the Concise Guide to PHARMACOLOGY 2021/22 (Alexander, Christopoulos et al., 2021; Alexander, Fabbro, et al., 2021; Alexander, Kelly et al., 2021).

## CONFLICT OF INTEREST

Michel Bouvier is President of the Domain Therapeutics Scientific Advisory Board. There are no other conflicts of interest to declare.

## APPENDIX A Experiment recommendation checklist

### A.1 Choosing a reference ligand

3.1 The choice of reference ligand distinguishes benchmark‐, pathway‐ or physiology‐bias

- Choose a reference ligand that can support the claims to be made.
- Include multiple reference ligands, thus allowing claims about different types of ligand bias.
- Measure the reference ligand(s) under identical conditions as the ligands tested for bias.
- If needed, use separate reference ligands for bias and Emax.

3.2 Ligand pathway‐bias (uses a pathway‐balanced reference ligand)

- Determine a pathway‐balanced reference ligand in a bias plot.

3.3 Ligand physiology‐bias (uses the principal endogenous ligand as reference)

- Use the principal endogenous agonist as reference ligand.

3.4 Ligand pathway‐preference (uses no reference ligand, and is not ligand bias)

- Compare pathway ΔLog (Emax/EC_50_) or ΔLog(τ/K_A_) values, not only fold potencies.
- Use the same or near‐identical systems and assays.

### A.2 Measuring at the transducer or downstream

4.1 Ligand bias measured at the transducer level

- Use recent assays consistently profiling G proteins and arrestins (also with GRKs).
- If possible, test all transducers for each investigated transducer family.
- If selecting representative transducer subtypes, use the most relevant.

4.2 Ligand bias measured downstream of distinct transducers

- Minimize differential signal amplification by measuring pathways at similar depth (estimated based on the number of upstream effectors).

4.3 Ligand bias measured downstream of converged transducer pathways

- Avoid measuring downstream of converged pathways.
- If possible, dissect upstream transducer contributions using other assays.

### A.3 Considering cellular system, kinetics and spatial bias

5.1 Bias may not translate across in vitro, physiological, and therapeutic systems

- Where possible, use primary and/or disease‐specific cells and evaluate potential system bias.
- Where possible, validate the effect in a model organism using an appropriate model of efficacy, and/or genetic engineering to confirm target and pathway specificity.

5.2 Kinetics and choosing measurement time points

- When possible, complete time courses and endpoint measurements should be made.
- Single time points should be the physiologically most relevant or the measure maximum effect.

### A.4 How to quantify ligand bias

6.1 Many models exist to quantify ligand bias

- It is not possible to recommend a single best practice quantification method.
- Results are more definitive when bias is quantified using multiple models.
- Irrespective of the model, error propagation and statistics must be handled appropriately.

6.2 A minimal and a refined model to calculate a ligand bias factor

- ΔΔLog (Emax/EC_50_) should not be used when ligand concentration‐response‐curve slope factors (Hill coefficients) are not close to 1.
- ΔΔLog(τ/K_A_) values are preferred over ΔΔLog (Emax/EC_50_) when ligand concentration‐response‐curve slope factors (Hill coefficients) are not close to 1.
- Confirm that the calculated bias factors are consistent with a bias plot.

6.3 Comparing ligand bias across studies and systems (use of rank orders)

- We recommend using ligand rank orders of bias factors (rather than quantitative bias values) for comparisons of ligand bias across studies using different experimental systems.

### A.5 Special recommendations for ‘tricky’ ligands

7.1 Low efficacy agonists

- Use ligand pathway rank orders.
- If quantifying bias, use another reference agonist for Emax.

7.2 Inverse agonists

- Use another inverse agonist as the reference ligand.

7.4 Allosteric modulators affecting ligand bias

- Quantification of allosteric modulator bias should make use of an extended operational model of allosterism.

## APPENDIX B Reporting recommendation checklist

### B.1 Choosing a reference ligand

3.1 The choice of reference ligand distinguishes benchmark‐, pathway‐ or physiology‐bias

- Report the reference ligand along with a motivation to why it was chosen.
- The claims should be restricted to what is supported by the chosen reference ligand.
- Define the bias type.

3.2 Ligand pathway‐bias (uses a pathway‐balanced reference ligand)

- Document the pathway‐balance of the reference ligand.

### B.2 Measuring at the transducer or downstream

4.1 Ligand bias measured at the transducer level

- Modifications of receptors, transducers or effectors must be clearly defined (e.g., tags, mutations, and chimeras).

4.2 Ligand bias measured downstream of distinct transducers

- Report measured processes.
- Report the measured molecules.

4.3 Ligand bias measured downstream of converged transducer pathways

- When pathways are truly inseparable and their contributions cannot be dissected using upstream assays, the bias may be described as a type of ‘effector bias’ (instead of pathway‐bias) accounting for the net pathway contributions.
- Interpret findings in light of their relative strength for the given receptor and ligand.

### B.3 Considering cellular system, kinetics and spatial bias

5.1 Bias may not translate across in vitro, physiological, and therapeutic systems

- Where a non‐native system has been used, conclusions should be stated carefully.

5.2 Kinetics and choosing measurement time points

- Report the chosen time points and the reason(s) why they were chosen.
- Provide data on the complete time course, if available.

5.3 Spatial bias: differing signaling efficacies across cellular compartments

- Report the biosensors and tags used for monitoring compartment‐specific signaling.
- Report the cell types used in assays.
- Report ligands with altered characteristics, e.g., from chemical modification.

### B.4 How to quantify ligand bias

6.2 A minimal and a refined model to calculate a ligand bias factor

- Report ligand Emax, EC_50_ and τ/K_A_ values and system maximum response, Em.
- Report the ligand concentration‐response‐curve slope factors (Hill coefficients).
- Report a bias plot for biased ligands.

### B.5 Special recommendations for ‘tricky’ ligands

7.3 Agonist and antagonist across pathways (‘modality bias’)

- Ligand bias with opposite modalities across pathways can be described as a non‐quantitative term, ‘modality bias’.
- Alternatively, it can be approximated by measuring an affinity to limit bias or describe it in a ‘bias is larger than’ relationship.

7.4 Allosteric modulators affecting ligand bias

- Report both the allosteric modulator and orthosteric ligand.

### B.6 Publication and database deposition

8.1 Unambiguous description of ligand bias

- Define the ligand evoking bias and its system in a clear sentence.
- Tabulate the experimental details required for unambiguous description (Table 3)
- Deposit biased ligands in a database.

## APPENDIX C Terminology cheat sheet

### C.1 Definitions of pathways and of bias types

2.1 Pathway definition and modulation

***Transducer:*** proteins that bind directly to an activated receptor to initiate, facilitate or modulate downstream events. This includes G proteins, GRKs and arrestins.

***Effector:*** Signaling protein located downstream in a transducer’s pathway.

***Modulator:*** Proteins or molecules that do not mediate, but modulate signaling of a receptor, transducer or effectors. (RAMPs, GEFs, GAPs, RGSes, NO, cholesterol, other lipids etc.

***Second messenger:*** Small molecules or ions directly controlled by the effectors. Examples include cAMP, calcium, etc.

***Pathway**:* A pathway is named after a transducer protein, or family thereof, that binds to GPCRs.

2.2 Ligand bias definition and distinction from system bias

***Ligand bias:*** Ligand‐dependent preferential receptor activation so that one over other transducer pathways in a given cellular system and relative to a reference ligand is induced.

***System bias:*** Bias due to differences in the cellular system, including so called ‘tissue bias’.

***Functional selectivity:*** Functional selectivity is the observed response combining ligand‐ and system‐bias.

***Observational bias:*** An artificial bias introduced by the experimental setup.

### C.2 Choosing a reference ligand

3.1 The choice of reference ligand distinguishes benchmark‐, pathway‐ or physiology‐bias

***Biased ligand:*** Ligand preferentially activating one receptor transducer pathway in a given cellular system and relative to a reference ligand. Ligand bias is a property of not just a ligand, but of a ligand, pathway pair and receptor in combination, and only valid within the specific investigated system. Therefore, the term ‘biased ligand’ should only be used if explicitly defined, and not be construed to represent a ligand‐only property. A recommended definition is included in section ‘Unambiguous description of ligand bias’, which provides one‐sentence and table templates for reporting.

***Reference ligand for bias:*** The ligand that is, by definition, unbiased. The bias of any other tested ligands is quantified relative to this reference.

***Reference ligand for E***_***max***_ ***:*** A separate reference ligand for the full receptor response (maximum efficacy, E_max_).

***Unbiased ligand:*** A ligand that stimulates pathways in a manner indistinguishable from the reference ligand.

3.2 Ligand pathway‐bias (uses a pathway‐balanced reference ligand)

***Balanced ligand:*** Has indistinguishable or very similar signaling through compared pathways.

***Ligand pathway‐bias:*** Ligand bias that is measured relative to a balanced reference ligand and therefore has the meaning that signaling is predominant via one pathway.

3.3 Ligand physiology‐bias (uses the principal endogenous ligand as reference)

***G protein selectivity***: The repertoire of G proteins that a receptor can engage. The term ‘natural bias’ is self‐contradictory and should not be used.

***Ligand physiology‐bias*:** Ligand bias relative to a receptor’s principal endogenous agonist, which therefore bears the meaning that signaling differs from the physiological.

3.4 Ligand pathway‐preference (uses no reference ligand, and is not ligand bias)

***Pathway‐preference*:** A ligand’s differential activity across pathways (e.g. pathway ΔLog (Emax/EC50) values), but without comparison to a reference ligand.

### C.3 Considering cellular system, kinetics and spatial bias

5.2 Kinetics and choosing measurement time points

***Temporal effect:*** The effect influencing a measured response due to the choice of time point at which a response is recorded.

5.3 Spatial bias: differing signaling efficacies across cellular compartments

***Spatial/location bias:*** The observation of biased GPCR signaling through the same transducer in different locations that results in distinct signaling responses.

### C.4 Special recommendations for ‘tricky’ ligands

7.3 Agonist and antagonist across pathways (‘modality bias’)

***Modality bias:*** Ligand with efficacy in only one of compared pathways (neutral antagonist or inverse agonist in others).

## References

[bph15811-bib-0001] Ahn, S. , Shenoy, S. K. , Luttrell, L. M. , & Lefkowitz, R. J. (2020). SnapShot: β‐arrestin functions. Cell, 182(1362–1362), e1361. 10.1016/j.cell.2020.07.034 32888497

[bph15811-bib-0002] Alexander, S. P. , Christopoulos, A. , Davenport, A. P. , Kelly, E. , Mathie, A. , Peters, J. A. , Veale, E. L. , Armstrong, J. F. , Faccenda, E. , Harding, S. D. , Pawson, A. J. , Southan, C. , Davies, J. A. , Abbracchio, M. P. , Alexander, W. , Al‐hosaini, K. , Bäck, M. , Barnes, N. M. , Bathgate, R. , … Ye, R. D. (2021). THE CONCISE GUIDE TO PHARMACOLOGY 2021/22: G protein‐coupled receptors. British Journal of Pharmacology, 178(S1), S27–S156. 10.1111/bph.15538 34529832

[bph15811-bib-0003] Alexander, S. P. , Fabbro, D. , Kelly, E. , Mathie, A. , Peters, J. A. , Veale, E. L. , Armstrong, J. F. , Faccenda, E. , Harding, S. D. , Pawson, A. J. , Southan, C. , Davies, J. A. , Boison, D. , Burns, K. E. , Dessauer, C. , Gertsch, J. , Helsby, N. A. , Izzo, A. A. , Koesling, D. , … Wong, S. S. (2021). THE CONCISE GUIDE TO PHARMACOLOGY 2021/22: Enzymes. British Journal of Pharmacology, 178(S1), S313–S411. 10.1111/bph.15542 34529828

[bph15811-bib-0004] Alexander, S. P. , Kelly, E. , Mathie, A. , Peters, J. A. , Veale, E. L. , Armstrong, J. F. , Faccenda, E. , Harding, S. D. , Pawson, A. J. , Southan, C. , Buneman, O. P. , Cidlowski, J. A. , Christopoulos, A. , Davenport, A. P. , Fabbro, D. , Spedding, M. , Striessnig, J. , Davies, J. A. , Ahlers‐Dannen, K. E. , … Zolghadri, Y. (2021). THE CONCISE GUIDE TO PHARMACOLOGY 2021/22: Other Protein Targets. British Journal of Pharmacology, 178(S1), S1–S26. 10.1111/bph.15537 34529830 PMC9513948

[bph15811-bib-0005] Anderson, A. , Masuho, I. , De Velasco, E. M. , Nakano, A. , Birnbaumer, L. , Martemyanov, K. A. , & Wickman, K. (2020). GPCR‐dependent biasing of GIRK channel signaling dynamics by RGS6 in mouse sinoatrial nodal cells. Proceedings of the National Academy of Sciences of the United States of America, 117, 14522–14531. 10.1073/pnas.2001270117 32513692 PMC7322085

[bph15811-bib-0006] Avet, C. , Mancini, A. , Breton, B. , Gouill, C. L. , Hauser, A. S. , Normand, C. , Kobayashi, H. , Gross, F. , Hogue, M. , Lukasheva, V. , & St‐Onge, S. (2021). Effector membrane translocation biosensors reveal G protein and B‐arrestin profiles of 100 therapeutically relevant GPCRs. bioRxiv: 2020.2004.2020.052027. 10.7554/eLife.74101 PMC9005190 35302493

[bph15811-bib-0007] Azzi, M. , Charest, P. G. , Angers, S. , Rousseau, G. , Kohout, T. , Bouvier, M. , & Pineyro, G. (2003). β‐Arrestin‐mediated activation of MAPK by inverse agonists reveals distinct active conformations for G protein‐coupled receptors. Proceedings of the National Academy of Sciences of the United States of America, 100, 11406–11411. 10.1073/pnas.1936664100 13679574 PMC208770

[bph15811-bib-0008] Baker, J. G. , Hall, I. P. , & Hill, S. J. (2003). Agonist and inverse agonist actions of β‐blockers at the human β2‐adrenoceptor provide evidence for agonist‐directed signaling. Molecular Pharmacology, 64, 1357–1369. 10.1124/mol.64.6.1357 14645666

[bph15811-bib-0009] Barlow, R. B. , Scott, N. C. , & Stephenson, R. P. (1967). The affinity and efficacy of onium salts on the frog rectus abdominis. British Journal of Pharmacology and Chemotherapy, 31, 188–196. 10.1111/j.1476-5381.1967.tb01989.x 6055253 PMC1557287

[bph15811-bib-0010] Black, J. W. , & Leff, P. (1983). Operational models of pharmacological agonism. Proceedings of the Royal Society of London ‐ Series B: Biological Sciences, 220, 141–162. 6141562 10.1098/rspb.1983.0093

[bph15811-bib-0011] Bock, A. , & Bermudez, M. (2021). Allosteric coupling and biased agonism in G protein‐coupled receptors. The FEBS Journal, 288, 2513–2528. 10.1111/febs.15783 33621418

[bph15811-bib-0012] Bockaert, J. , Fagni, L. , Dumuis, A. , & Marin, P. (2004). GPCR interacting proteins (GIP). Pharmacology & Therapeutics, 103, 203–221. 10.1016/j.pharmthera.2004.06.004 15464590

[bph15811-bib-0013] Bohn, L. M. , & McDonald, P. H. (2010). Seeking ligand bias: Assessing GPCR coupling to β‐arrestins for drug discovery. Drug Discovery Today: Technologies, 7, e37–e42. 10.1016/j.ddtec.2010.06.005 21218149 PMC3014586

[bph15811-bib-0014] Bohn, L. M. , Zhou, L. , & Ho, J. H. (2015). Approaches to assess functional selectivity in GPCRs: Evaluating G protein signaling in an endogenous environment. Methods in Molecular Biology, 1335, 177–189. 10.1007/978-1-4939-2914-6_12 26260601 PMC4804702

[bph15811-bib-0015] Bouvier, M. , Leeb‐Lundberg, L. M. , Benovic, J. L. , Caron, M. G. , & Lefkowitz, R. J. (1987). Regulation of adrenergic receptor function by phosphorylation. II. Effects of agonist occupancy on phosphorylation of α1‐ and β2‐adrenergic receptors by protein kinase C and the cyclic AMP‐dependent protein kinase. The Journal of Biological Chemistry, 262, 3106–3113. 10.1016/S0021-9258(18)61475-9 3029101

[bph15811-bib-0016] Bradley, S. J. , & Tobin, A. B. (2016). Design of next‐generation G protein‐coupled receptor drugs: Linking novel pharmacology and in vivo animal models. Annual Review of Pharmacology and Toxicology, 56, 535–559. 10.1146/annurev-pharmtox-011613-140012 26738479

[bph15811-bib-0017] Carman, C. V. , Parent, J. L. , Day, P. W. , Pronin, A. N. , Sternweis, P. M. , Wedegaertner, P. B. , Gilman, A. G. , Benovic, J. L. , & Kozasa, T. (1999). Selective regulation of Gα(q/11) by an RGS domain in the G protein‐coupled receptor kinase, GRK2. The Journal of Biological Chemistry, 274, 34483–34492. 10.1074/jbc.274.48.34483 10567430

[bph15811-bib-0018] Che, T. , Dwivedi‐Agnihotri, H. , Shukla, A. K. , & Roth, B. L. (2021). Biased ligands at opioid receptors: Current status and future directions. Science Signaling, 14, eaav0320. 10.1126/scisignal.aav0320 33824179 PMC7611221

[bph15811-bib-0019] Chidiac, P. , Nouet, S. , & Bouvier, M. (1996). Agonist‐induced modulation of inverse agonist efficacy at the β2‐adrenergic receptor. Molecular Pharmacology, 50, 662–669. 8794908

[bph15811-bib-0020] Crilly, S. E. , & Puthenveedu, M. A. (2021). Compartmentalized GPCR signaling from intracellular membranes. The Journal of Membrane Biology, 254, 259–271. 10.1007/s00232-020-00158-7 33231722 PMC8141539

[bph15811-bib-0021] De Lean, A. , Stadel, J. M. , & Lefkowitz, R. J. (1980). A ternary complex model explains the agonist‐specific binding properties of the adenylate cyclase‐coupled β‐adrenergic receptor. The Journal of Biological Chemistry, 255, 7108–7117. 6248546

[bph15811-bib-0022] Dean, T. , Vilardaga, J. P. , Potts, J. T. Jr. , & Gardella, T. J. (2008). Altered selectivity of parathyroid hormone (PTH) and PTH‐related protein (PTHrP) for distinct conformations of the PTH/PTHrP receptor. Molecular Endocrinology, 22, 156–166. 10.1210/me.2007-0274 17872377 PMC2194631

[bph15811-bib-0023] DebBurman, S. K. , Ptasienski, J. , Benovic, J. L. , & Hosey, M. M. (1996). G protein‐coupled receptor kinase GRK2 is a phospholipid‐dependent enzyme that can be conditionally activated by G protein βγ subunits. The Journal of Biological Chemistry, 271, 22552–22562. 10.1074/jbc.271.37.22552 8798423

[bph15811-bib-0024] Dorn, G. W. 2nd , Oswald, K. J. , McCluskey, T. S. , Kuhel, D. G. , & Liggett, S. B. (1997). α2A‐Adrenergic receptor stimulated calcium release is transduced by Gi‐associated G (βγ)‐mediated activation of phospholipase C. Biochemistry, 36, 6415–6423. 9174358 10.1021/bi970080s

[bph15811-bib-0025] Ehlert, F. J. (2008). On the analysis of ligand‐directed signaling at G protein‐coupled receptors. Naunyn‐Schmiedeberg's Archives of Pharmacology, 377, 549–577. 10.1007/s00210-008-0260-4 18253722

[bph15811-bib-0026] Ehlert, F. J. , Suga, H. , & Griffin, M. T. (2011). Analysis of agonism and inverse agonism in functional assays with constitutive activity: Estimation of orthosteric ligand affinity constants for active and inactive receptor states. The Journal of Pharmacology and Experimental Therapeutics, 338, 671–686. 10.1124/jpet.111.179309 21576379 PMC3141894

[bph15811-bib-0027] Eichel, K. , Jullie, D. , Barsi‐Rhyne, B. , Latorraca, N. R. , Masureel, M. , Sibarita, J. B. , Dror, R. O. , & von Zastrow, M. (2018). Catalytic activation of β‐arrestin by GPCRs. Nature, 557, 381–386. 10.1038/s41586-018-0079-1 29720660 PMC6058965

[bph15811-bib-0028] Eichel, K. , Jullie, D. , & von Zastrow, M. (2016). β‐Arrestin drives MAP kinase signalling from clathrin‐coated structures after GPCR dissociation. Nature Cell Biology, 18, 303–310. 10.1038/ncb3307 26829388 PMC4767649

[bph15811-bib-0029] Feinstein, T. N. , Wehbi, V. L. , Ardura, J. A. , Wheeler, D. S. , Ferrandon, S. , Gardella, T. J. , & Vilardaga, J. P. (2011). Retromer terminates the generation of cAMP by internalized PTH receptors. Nature Chemical Biology, 7, 278–284. 10.1038/nchembio.545 21445058 PMC3079799

[bph15811-bib-0030] Furchott, R. F. (1966). The use of β‐haloaklylamines in the differentiation of the receptors and in the determination of dissociation constants of receptor–agonist complexes. In N. J. A. S. Harper (Ed.), Adv drug res (pp. 21–55). A.B. Academic Press.

[bph15811-bib-0031] Galandrin, S. , Oligny‐Longpre, G. , & Bouvier, M. (2007). The evasive nature of drug efficacy: Implications for drug discovery. Trends in Pharmacological Sciences, 28, 423–430. 10.1016/j.tips.2007.06.005 17659355

[bph15811-bib-0032] Gales, C. , Van Durm, J. J. , Schaak, S. , Pontier, S. , Percherancier, Y. , Audet, M. , Paris, H. , & Bouvier, M. (2006). Probing the activation‐promoted structural rearrangements in preassembled receptor‐G protein complexes. Nature Structural & Molecular Biology, 13, 778–786. 10.1038/nsmb1134 16906158

[bph15811-bib-0033] Ghosh, E. , Dwivedi, H. , Baidya, M. , Srivastava, A. , Kumari, P. , Stepniewski, T. , Kim, H. R. , Lee, M. H. , van Gastel, J. , Chaturvedi, M. , & Roy, D. (2019). Conformational sensors and domain swapping reveal structural and functional differences between β‐arrestin isoforms. Cell Reports, 28, 3287–3299. 31553900 10.1016/j.celrep.2019.08.053 PMC7099875

[bph15811-bib-0034] Gomes, I. , Sierra, S. , Lueptow, L. , Gupta, A. , Gouty, S. , Margolis, E. B. , Cox, B. M. , & Devi, L. A. (2020). Biased signaling by endogenous opioid peptides. Proceedings of the National Academy of Sciences of the United States of America, 117, 11820–11828. 10.1073/pnas.2000712117 32393639 PMC7261131

[bph15811-bib-0035] Grundmann, M. , Merten, N. , Malfacini, D. , Inoue, A. , Preis, P. , Simon, K. , Rüttiger, N. , Ziegler, N. , Benkel, T. , Schmitt, N. K. , Ishida, S. , Müller, I. , Reher, R. , Kawakami, K. , Inoue, A. , Rick, U. , Kühl, T. , Imhof, D. , Aoki, J. , … Kostenis, E. (2018). Lack of β‐arrestin signaling in the absence of active G proteins. Nature Communications, 9, 1–16. 10.1038/s41467-017-02661-3 PMC5780443 29362459

[bph15811-bib-0036] Gurevich, V. V. , & Benovic, J. L. (1997). Mechanism of phosphorylation‐recognition by visual arrestin and the transition of arrestin into a high affinity binding state. Molecular Pharmacology, 51, 161–169. 10.1124/mol.51.1.161 9016359

[bph15811-bib-0037] Gurevich, V. V. , & Gurevich, E. V. (2018). Arrestin‐mediated signaling: Is there a controversy? World Journal of Biological Chemistry, 9, 25–35. 10.4331/wjbc.v9.i3.25 30595812 PMC6305498

[bph15811-bib-0038] Gutkind, J. S. , & Kostenis, E. (2018). Arrestins as rheostats of GPCR signalling. Nature Reviews. Molecular Cell Biology, 19, 615–616. 10.1038/s41580-018-0041-y 30026541

[bph15811-bib-0039] Harding, S. D. , Armstrong, J. F. , Faccenda, E. , Southan, C. , Alexander, S. P. H. , Davenport, A. P. , Pawson, A. J. , Spedding, M. , Davies, J. A. , & NC‐IUPHAR . (2021). The IUPHAR/BPS guide to pharmacology in 2022: Curating pharmacology for COVID‐19, malaria and antibacterials. Nucleic Acids Research, 50, D1282–D1294. 10.1093/nar/gkab1010 PMC8689838 34718737

[bph15811-bib-0040] Hasenhuetl, P. S. , Bhat, S. , Freissmuth, M. , & Sandtner, W. (2019). Functional selectivity and partial efficacy at the monoamine transporters: A unified model of allosteric modulation and amphetamine‐induced substrate release. Molecular Pharmacology, 95, 303–312. 10.1124/mol.118.114793 30567955

[bph15811-bib-0041] Hausdorff, W. P. , Bouvier, M. , O'Dowd, B. F. , Irons, G. P. , Caron, M. G. , & Lefkowitz, R. J. (1989). Phosphorylation sites on two domains of the β2‐adrenergic receptor are involved in distinct pathways of receptor desensitization. The Journal of Biological Chemistry, 264, 12657–12665. 10.1016/S0021-9258(18)63907-9 2545714

[bph15811-bib-0042] Hauser, A. S. , Attwood, M. M. , Rask‐Andersen, M. , Schioth, H. B. , & Gloriam, D. E. (2017). Trends in GPCR drug discovery: New agents, targets and indications. Nature Reviews. Drug Discovery, 16, 829–842. 10.1038/nrd.2017.178 29075003 PMC6882681

[bph15811-bib-0043] Hay, D. L. , Garelja, M. L. , Poyner, D. R. , & Walker, C. S. (2018). Update on the pharmacology of calcitonin/CGRP family of peptides: IUPHAR review 25. British Journal of Pharmacology, 175, 3–17. 10.1111/bph.14075 29059473 PMC5740251

[bph15811-bib-0044] Hay, D. L. , & Pioszak, A. A. (2016). Receptor activity‐modifying proteins (RAMPs): New insights and roles. Annual Review of Pharmacology and Toxicology, 56, 469–487. 10.1146/annurev-pharmtox-010715-103120 PMC5559101 26514202

[bph15811-bib-0045] Heidari, Z. , Chrisman, I. M. , Nemetchek, M. D. , Novick, S. J. , Blayo, A. L. , Patton, T. , Mendes, D. E. , Diaz, P. , Kamenecka, T. M. , Griffin, P. R. , & Hughes, T. S. (2019). Definition of functionally and structurally distinct repressive states in the nuclear receptor PPARγ. Nature Communications, 10, 5825. 10.1038/s41467-019-13768-0 PMC6925260 31862968

[bph15811-bib-0046] Hillenbrand, M. , Schori, C. , Schoppe, J. , & Plückthun, A. (2015). Comprehensive analysis of heterotrimeric G‐protein complex diversity and their interactions with GPCRs in solution. Proceedings of the National Academy of Sciences of the United States of America, 112, E1181–E1190. 10.1073/pnas.1417573112 25733868 PMC4371982

[bph15811-bib-0047] Ho, M. K. C. , & Wong, Y. H. (2001). Gz signaling: Emerging divergence from Gi signaling. Oncogene, 20, 1615–1625. 10.1038/sj.onc.1204190 11313909

[bph15811-bib-0048] Hoare, S. R. J. , Pierre, N. , Moya, A. G. , & Larson, B. (2018). Kinetic operational models of agonism for G‐protein‐coupled receptors. Journal of Theoretical Biology, 446, 168–204. 10.1016/j.jtbi.2018.02.014 29486201

[bph15811-bib-0049] Hollenberg, M. D. , Mihara, K. , Polley, D. , Suen, J. Y. , Han, A. , Fairlie, D. P. , & Ramachandran, R. (2014). Biased signalling and proteinase‐activated receptors (PARs): Targeting inflammatory disease. British Journal of Pharmacology, 171, 1180–1194. 10.1111/bph.12544 24354792 PMC3952797

[bph15811-bib-0050] Hollinger, S. , & Hepler, J. R. (2002). Cellular regulation of RGS proteins: Modulators and integrators of G protein signaling. Pharmacological Reviews, 54, 527–559. 10.1124/pr.54.3.527 12223533

[bph15811-bib-0051] Hunton, D. L. , Barnes, W. G. , Kim, J. , Ren, X. R. , Violin, J. D. , Reiter, E. , Milligan, G. , Patel, D. D. , & Lefkowitz, R. J. (2005). β‐Arrestin 2‐dependent angiotensin II type 1A receptor‐mediated pathway of chemotaxis. Molecular Pharmacology, 67, 1229–1236. 10.1124/mol.104.006270 15635042

[bph15811-bib-0052] Inoue, A. , Raimondi, F. , Kadji, F. M. N. , Singh, G. , Kishi, T. , Uwamizu, A. , Ono, Y. , Shinjo, Y. , Ishida, S. , Arang, N. , & Kawakami, K. (2019). Illuminating G‐protein‐coupling selectivity of GPCRs. Cell, 177, 1933–1947. 31160049 10.1016/j.cell.2019.04.044 PMC6773469

[bph15811-bib-0053] Irannejad, R. , Pessino, V. , Mika, D. , Huang, B. , Wedegaertner, P. B. , Conti, M. , & von Zastrow, M. (2017). Functional selectivity of GPCR‐directed drug action through location bias. Nature Chemical Biology, 13, 799–806. 10.1038/nchembio.2389 28553949 PMC5733145

[bph15811-bib-0054] Jain, R. , Watson, U. , Vasudevan, L. , & Saini, D. K. (2018). Chapter Three ‐ ERK activation pathways downstream of GPCRs. In A. K. Shukla (Ed.), Int rev cell Mol biol (pp. 79–109). Academic Press. 10.1016/bs.ircmb.2018.02.003 29699693

[bph15811-bib-0055] Jensen, D. D. , Lieu, T. , Halls, M. L. , Veldhuis, N. A. , Imlach, W. L. , Mai, Q. N. , Poole, D. P. , Quach, T. , Aurelio, L. , Conner, J. , Herenbrink, C. K. , Barlow, N. , Simpson, J. S. , Scanlon, M. J. , Graham, B. , McCluskey, A. , Robinson, P. J. , Escriou, V. , Nassini, R. , … Bunnett, N. W. (2017). Neurokinin 1 receptor signaling in endosomes mediates sustained nociception and is a viable therapeutic target for prolonged pain relief. Science Translational Medicine, 9, 1–5. 10.1126/scitranslmed.aal3447 PMC6034632 28566424

[bph15811-bib-0056] Jensen, S. B. , Thodberg, S. , Parween, S. , Moses, M. E. , Hansen, C. C. , Thomsen, J. , Sletfjerding, M. B. , Knudsen, C. , del Giudice, R. , Lund, P. M. , Castaño, P. R. , Bustamante, Y. G. , Velazquez, M. N. R. , Jørgensen, F. S. , Pandey, A. V. , Laursen, T. , Møller, B. L. , & Hatzakis, N. S. (2021). Biased cytochrome P450‐mediated metabolism via small‐molecule ligands binding P450 oxidoreductase. Nature Communications, 12, 1–14. 10.1038/s41467-021-22562-w PMC8050233 33859207

[bph15811-bib-0057] Jiang, M. , & Bajpayee, N. S. (2009). Molecular mechanisms of go signaling. Neurosignals, 17, 23–41. 10.1159/000186688 19212138 PMC2836949

[bph15811-bib-0058] Jong, Y. I. , Harmon, S. K. , & O'Malley, K. L. (2019). Location and cell‐type‐specific bias of metabotropic glutamate receptor, mGlu5, negative allosteric modulators. ACS Chemical Neuroscience, 10, 4558–4570. 10.1021/acschemneuro.9b00415 31609579 PMC8753982

[bph15811-bib-0059] Karl, K. , Paul, M. D. , Pasquale, E. B. , & Hristova, K. (2020). Ligand bias in receptor tyrosine kinase signaling. The Journal of Biological Chemistry, 295, 18494–18507. 10.1074/jbc.REV120.015190 33122191 PMC7939482

[bph15811-bib-0060] Kenakin, T. (1995). Agonist‐receptor efficacy. II. Agonist trafficking of receptor signals. Trends in Pharmacological Sciences, 16, 232–238. 10.1016/S0165-6147(00)89032-X 7667897

[bph15811-bib-0061] Kenakin, T. (1997). Differences between natural and recombinant G protein‐coupled receptor systems with varying receptor/G protein stoichiometry. Trends in Pharmacological Sciences, 18, 456–464. 10.1016/S0165-6147(97)01136-X 9458693

[bph15811-bib-0062] Kenakin, T. (2015a). The effective application of biased signaling to new drug discovery. Molecular Pharmacology, 88, 1055–1061. 10.1124/mol.115.099770 26138073

[bph15811-bib-0063] Kenakin, T. (2015b). The measurement of receptor signaling bias. Methods in Molecular Biology, 1335, 163–176. 10.1007/978-1-4939-2914-6_11 26260600

[bph15811-bib-0064] Kenakin, T. (2019). Biased receptor signaling in drug discovery. Pharmacological Reviews, 71, 267–315. 10.1124/pr.118.016790 30914442

[bph15811-bib-0065] Kenakin, T. , & Christopoulos, A. (2013). Signalling bias in new drug discovery: Detection, quantification and therapeutic impact. Nature Reviews. Drug Discovery, 12, 205–216. 10.1038/nrd3954 23411724

[bph15811-bib-0066] Kenakin, T. P. , & Morgan, P. H. (1989). Theoretical effects of single and multiple transducer receptor coupling proteins on estimates of the relative potency of agonists. Molecular Pharmacology, 35, 214–222. 2537459

[bph15811-bib-0067] Klein Herenbrink, C. , Sykes, D. A. , Donthamsetti, P. , Canals, M. , Coudrat, T. , Shonberg, J. , Scammells, P. J. , Capuano, B. , Sexton, P. M. , Charlton, S. J. , Javitch, J. A. , Christopoulos, A. , & Lane, J. R. (2016). The role of kinetic context in apparent biased agonism at GPCRs. Nature Communications, 7, 1–15. 10.1038/ncomms10842 PMC4770093 26905976

[bph15811-bib-0068] Kohout, T. A. , Nicholas, S. L. , Perry, S. J. , Reinhart, G. , Junger, S. , & Struthers, R. S. (2004). Differential desensitization, receptor phosphorylation, β‐arrestin recruitment, and ERK1/2 activation by the two endogenous ligands for the CC chemokine receptor 7. The Journal of Biological Chemistry, 279, 23214–23222. 10.1074/jbc.M402125200 15054093

[bph15811-bib-0069] Komolov, K. E. , & Benovic, J. L. (2018). G protein‐coupled receptor kinases: Past, present and future. Cellular Signalling, 41, 17–24. 10.1016/j.cellsig.2017.07.004 28711719 PMC5722692

[bph15811-bib-0070] Kooistra, A. J. , Mordalski, S. , Pandy‐Szekeres, G. , Esguerra, M. , Mamyrbekov, A. , Munk, C. , Keserű, G. M. , & Gloriam, D. E. (2021). GPCRdb in 2021: Integrating GPCR sequence, structure and function. Nucleic Acids Research, 49, D335–D343. 10.1093/nar/gkaa1080 33270898 PMC7778909

[bph15811-bib-0071] Lane, J. R. , May, L. T. , Parton, R. G. , Sexton, P. M. , & Christopoulos, A. (2017). A kinetic view of GPCR allostery and biased agonism. Nature Chemical Biology, 13, 929–937. 10.1038/nchembio.2431 28820879

[bph15811-bib-0072] Leach, K. , Sexton, P. M. , & Christopoulos, A. (2007). Allosteric GPCR modulators: Taking advantage of permissive receptor pharmacology. Trends in Pharmacological Sciences, 28, 382–389. 10.1016/j.tips.2007.06.004 17629965

[bph15811-bib-0073] Lefkowitz, R. J. , Mullikin, D. , & Caron, M. G. (1976). Regulation of β‐adrenergic receptors by guanyl‐5′‐yl imidodiphosphate and other purine nucleotides. The Journal of Biological Chemistry, 251, 4686–4692. 10.1016/S0021-9258(17)33257-X 947904

[bph15811-bib-0074] Lukasheva, V. , Devost, D. , Le Gouill, C. , Namkung, Y. , Martin, R. D. , Longpré, J.‐M. , Amraei, M. , Shinjo, Y. , Hogue, M. , Lagacé, M. , & Breton, B. (2020). Signal profiling of the β1AR reveals coupling to novel signalling pathways and distinct phenotypic responses mediated by β1AR and β2AR. Scientific Reports, 10, 8779. 10.1038/s41598-020-65636-3 32471984 PMC7260363

[bph15811-bib-0075] Luttrell, L. M. , Maudsley, S. , & Gesty‐Palmer, D. (2018). Translating in vitro ligand bias into in vivo efficacy. Cellular Signalling, 41, 46–55. 10.1016/j.cellsig.2017.05.002 28495495 PMC5675828

[bph15811-bib-0076] Luttrell, L. M. , Roudabush, F. L. , Choy, E. W. , Miller, W. E. , Field, M. E. , Pierce, K. L. , & Lefkowitz, R. J. (2001). Activation and targeting of extracellular signal‐regulated kinases by β‐arrestin scaffolds. Proceedings of the National Academy of Sciences of the United States of America, 98, 2449–2454. 10.1073/pnas.041604898 11226259 PMC30158

[bph15811-bib-0077] Luttrell, L. M. , Wang, J. , Plouffe, B. , Smith, J. S. , Yamani, L. , Kaur, S. , Jean‐Charles, P. Y. , Gauthier, C. , Lee, M. H. , Pani, B. , & Kim, J. (2018). Manifold roles of β‐arrestins in GPCR signaling elucidated with siRNA and CRISPR/Cas9. Science Signaling, 11, eaat7650. 30254056 10.1126/scisignal.aat7650 PMC6369040

[bph15811-bib-0078] Maguire, M. E. , Van Arsdale, P. M. , & Gliman, A. G. (1976). An agonist‐specific effect of guanine nucleotides on binding to the β‐adrenergic receptor. Molecular Pharmacology, 12, 335–339. 4726

[bph15811-bib-0079] Masuho, I. , Balaji, S. , Muntean, B. S. , Skamangas, N. K. , Chavali, S. , Tesmer, J. J. G. , Babu, M. M. , & Martemyanov, K. A. (2020). A global map of G protein signaling regulation by RGS proteins. Cell, 183, 503–521.e519. 10.1016/j.cell.2020.08.052 33007266 PMC7572916

[bph15811-bib-0080] Masuho, I. , Martemyanov, K. A. , & Lambert, N. A. (2015). Monitoring G protein activation in cells with BRET. Methods in Molecular Biology, 1335, 107–113. 10.1007/978-1-4939-2914-6_8 26260597 PMC4879496

[bph15811-bib-0081] Masuho, I. , Ostrovskaya, O. , Kramer, G. M. , Jones, C. D. , Xie, K. , & Martemyanov, K. A. (2015). Distinct profiles of functional discrimination among G proteins determine the actions of G protein‐coupled receptors. Science Signaling, 8, ra123. 26628681 10.1126/scisignal.aab4068 PMC4886239

[bph15811-bib-0082] Masuho, I. , Skamangas, N. K. , Muntean, B. S. , & Martemyanov, K. A. (2021). Diversity of the Gβγ complexes defines spatial and temporal bias of GPCR signaling. Cell Systems, 12, 324–337. 33667409 10.1016/j.cels.2021.02.001 PMC8068604

[bph15811-bib-0083] Maurice, P. , Guillaume, J. L. , Benleulmi‐Chaachoua, A. , Daulat, A. M. , Kamal, M. , & Jockers, R. (2011). GPCR‐interacting proteins, major players of GPCR function. Advances in Pharmacology, 62, 349–380. 10.1016/B978-0-12-385952-5.00001-4 21907915

[bph15811-bib-0084] Meyrath, M. , Szpakowska, M. , Zeiner, J. , Massotte, L. , Merz, M. P. , Benkel, T. , Simon, K. , Ohnmacht, J. , Turner, J. D. , Krüger, R. , Seutin, V. , Ollert, M. , Kostenis, E. , & Chevigné, A. (2020). The atypical chemokine receptor ACKR3/CXCR7 is a broad‐spectrum scavenger for opioid peptides. Nature Communications, 11, 1–16. 10.1038/s41467-020-16664-0 PMC7305236 32561830

[bph15811-bib-0085] Milligan, G. , & Kostenis, E. (2006). Heterotrimeric G‐proteins: A short history. British Journal of Pharmacology, 147(Suppl 1), S46–S55. 10.1038/sj.bjp.0706405 16402120 PMC1760735

[bph15811-bib-0086] Namkung, Y. , Le Gouill, C. , Lukashova, V. , Kobayashi, H. , Hogue, M. , Khoury, E. , Song, M. , Bouvier, M. , & Laporte, S. A. (2016). Monitoring G protein‐coupled receptor and β‐arrestin trafficking in live cells using enhanced bystander BRET. Nature Communications, 7, 12178. 10.1038/ncomms12178 PMC4942582 27397672

[bph15811-bib-0087] Namkung, Y. , LeGouill, C. , Kumar, S. , Cao, Y. , Teixeira, L. B. , Lukasheva, V. , Giubilaro, J. , Simões, S. C. , Longpré, J. M. , Devost, D. , Hébert, T. E. , Piñeyro, G. , Leduc, R. , Costa‐Neto, C. M. , Bouvier, M. , & Laporte, S. A. (2018). Functional selectivity profiling of the angiotensin II type 1 receptor using pathway‐wide BRET signaling sensors. Science Signaling, 11, 1–12. 10.1126/scisignal.aat1631 30514808

[bph15811-bib-0088] Neubig, R. R. , & Siderovski, D. P. (2002). Regulators of G‐protein signalling as new central nervous system drug targets. Nature Reviews. Drug Discovery, 1, 187–197. 10.1038/nrd747 12120503

[bph15811-bib-0089] Oliver, E. E. , Hughes, E. K. , Puckett, M. K. , Chen, R. , Lowther, W. T. , & Howlett, A. C. (2020). Cannabinoid receptor interacting protein 1a (CRIP1a) in health and disease. Biomolecules, 10, 1–22. 10.3390/biom10121609 PMC7761089 33261012

[bph15811-bib-0090] Olsen, R. H. J. , DiBerto, J. F. , English, J. G. , Glaudin, A. M. , Krumm, B. E. , Slocum, S. T. , Che, T. , Gavin, A. C. , McCorvy, J. D. , Roth, B. L. , & Strachan, R. T. (2020). TRUPATH, an open‐source biosensor platform for interrogating the GPCR transducerome. Nature Chemical Biology, 16, 841–849. 10.1038/s41589-020-0535-8 32367019 PMC7648517

[bph15811-bib-0091] Onaran, H. O. , Ambrosio, C. , Ugur, O. , Madaras Koncz, E. , Gro, M. C. , Vezzi, V. , Rajagopal, S. , & Costa, T. (2017). Systematic errors in detecting biased agonism: Analysis of current methods and development of a new model‐free approach. Scientific Reports, 7, 44247. 10.1038/srep44247 28290478 PMC5349545

[bph15811-bib-0092] Onaran, H. O. , & Costa, T. (2021). Conceptual and experimental issues in biased agonism. Cellular Signalling, 82, 109955. 10.1016/j.cellsig.2021.109955 33607257

[bph15811-bib-0093] Pandey, S. , Kumari, P. , Baidya, M. , Kise, R. , Cao, Y. , Dwivedi‐Agnihotri, H. , Banerjee, R. , Li, X. X. , Cui, C. S. , Lee, J. D. , Kawakami, K. , Maharana, J. , Ranjan, A. , Chaturvedi, M. , Jhingan, G. D. , Laporte, S. A. , Woodruff, T. M. , Inoue, A. , & Shukla, A. K. (2021). Intrinsic bias at non‐canonical, β‐arrestin‐coupled seven transmembrane receptors. Molecular Cell, 81, 4605–4621. 10.1016/j.molcel.2021.09.007 34582793 PMC7612807

[bph15811-bib-0094] Pandy‐Szekeres, G. , Esguerra, M. , Hauser, A. S. , Caroli, J. , Munk, C. , Pilger, S. , Keserű, G. M. , Kooistra, A. J. , & Gloriam, D. E. (2022). The G protein database, GproteinDb. Nucleic Acids Research, 50, D518–D525. 10.1093/nar/gkab852 34570219 PMC8728128

[bph15811-bib-0095] Perry‐Hauser, N. A. , Asher, W. B. , Hauge Pedersen, M. , & Javitch, J. A. (2021). Chapter 3 ‐ Assays for detecting arrestin interaction with GPCRs. In A. K. Shukla (Ed.), Methods cell biol (pp. 43–65). Academic Press. 10.1016/bs.mcb.2021.06.007 34752339

[bph15811-bib-0096] Rajagopal, S. , Ahn, S. , Rominger, D. H. , Gowen‐MacDonald, W. , Lam, C. M. , DeWire, S. M. , Violin, J. D. , & Lefkowitz, R. J. (2011). Quantifying ligand bias at seven‐transmembrane receptors. Molecular Pharmacology, 80, 367–377. 10.1124/mol.111.072801 21610196 PMC3164332

[bph15811-bib-0097] Rajagopal, S. , Kim, J. , Ahn, S. , Craig, S. , Lam, C. M. , Gerard, N. P. , Gerard, C. , & Lefkowitz, R. J. (2010). β‐Arrestin‐ but not G protein‐mediated signaling by the “decoy” receptor CXCR7. Proceedings of the National Academy of Sciences of the United States of America, 107, 628–632. 10.1073/pnas.0912852107 20018651 PMC2818968

[bph15811-bib-0098] Ribas, C. , Penela, P. , Murga, C. , Salcedo, A. , Garcia‐Hoz, C. , Jurado‐Pueyo, M. , Aymerich, I. , & Mayor, F. Jr. (2007). The G protein‐coupled receptor kinase (GRK) interactome: Role of GRKs in GPCR regulation and signaling. Biochimica et Biophysica Acta, 1768, 913–922. 10.1016/j.bbamem.2006.09.019 17084806

[bph15811-bib-0099] Roth, B. L. , & Chuang, D. M. (1987). Multiple mechanisms of serotonergic signal transduction. Life Sciences, 41, 1051–1064. 10.1016/0024-3205(87)90621-7 2441225

[bph15811-bib-0100] Sauliere, A. , Bellot, M. , Paris, H. , Denis, C. , Finana, F. , Hansen, J. T. , Altié, M. F. , Seguelas, M. H. , Pathak, A. , Hansen, J. L. , & Sénard, J. M. (2012). Deciphering biased‐agonism complexity reveals a new active AT1 receptor entity. Nature Chemical Biology, 8, 622–630. 10.1038/nchembio.961 22634635

[bph15811-bib-0101] Schmid, C. L. , Raehal, K. M. , & Bohn, L. M. (2008). Agonist‐directed signaling of the serotonin 2A receptor depends on β‐arrestin‐2 interactions in vivo. Proceedings of the National Academy of Sciences of the United States of America, 105, 1079–1084. 10.1073/pnas.0708862105 18195357 PMC2242710

[bph15811-bib-0102] Slosky, L. M. , Caron, M. G. , & Barak, L. S. (2021). Biased allosteric modulators: New frontiers in GPCR drug discovery. Trends in Pharmacological Sciences, 42, 283–299. 10.1016/j.tips.2020.12.005 33581873 PMC9797227

[bph15811-bib-0103] Smith, J. S. , Lefkowitz, R. J. , & Rajagopal, S. (2018). Biased signalling: From simple switches to allosteric microprocessors. Nature Reviews. Drug Discovery, 17, 243–260. 10.1038/nrd.2017.229 29302067 PMC5936084

[bph15811-bib-0104] Smith, J. S. , Pack, T. F. , Inoue, A. , Lee, C. , Zheng, K. , Choi, I. , Eiger, D. S. , Warman, A. , Xiong, X. , Ma, Z. , Viswanathan, G. , Levitan, I. M. , Rochelle, L. K. , Staus, D. P. , Snyder, J. C. , Kahsai, A. W. , Caron, M. G. , & Rajagopal, S. (2021). Noncanonical scaffolding of Gαi and β‐arrestin by G protein‐coupled receptors. Science, 371, eaay1833. 10.1126/science.aay1833 33479120 PMC8005335

[bph15811-bib-0105] Sommer, M. E. , Selent, J. , Carlsson, J. , De Graaf, C. , Gloriam, D. E. , Keseru, G. M. , Kosloff, M. , Mordalski, S. , Rizk, A. , Rosenkilde, M. M. , & Sotelo, E. (2020). The European Research Network on Signal Transduction (ERNEST): Toward a multidimensional holistic understanding of G protein‐coupled receptor signaling. ACS Pharmacology & Translational Science, 3, 361–370. 10.1021/acsptsci.0c00024 32296774 PMC7155379

[bph15811-bib-0106] Spengler, D. , Waeber, C. , Pantaloni, C. , Holsboer, F. , Bockaert, J. , Seeburg, P. H. , & Journot, L. (1993). Differential signal transduction by five splice variants of the PACAP receptor. Nature, 365, 170–175. 8396727 10.1038/365170a0

[bph15811-bib-0107] Sriram, K. , & Insel, P. A. (2018). G protein‐coupled receptors as targets for approved drugs: How many targets and how many drugs? Molecular Pharmacology, 93, 251–258. 10.1124/mol.117.111062 29298813 PMC5820538

[bph15811-bib-0108] Srivastava, A. , Gupta, B. , Gupta, C. , & Shukla, A. K. (2015). Emerging functional divergence of β‐arrestin isoforms in GPCR function. Trends in Endocrinology and Metabolism, 26, 628–642. 10.1016/j.tem.2015.09.001 26471844

[bph15811-bib-0109] Stahl, E. L. , Ehlert, F. J. , & Bohn, L. M. (2019). Quantitating ligand bias using the competitive model of ligand activity. Methods in Molecular Biology, 1957, 235–247. 10.1007/978-1-4939-9158-7_15 30919358

[bph15811-bib-0110] Stahl, E. L. , Zhou, L. , Ehlert, F. J. , & Bohn, L. M. (2015). A novel method for analyzing extremely biased agonism at G protein‐coupled receptors. Molecular Pharmacology, 87, 866–877. 10.1124/mol.114.096503 25680753 PMC4407734

[bph15811-bib-0111] Stallaert, W. , Christopoulos, A. , & Bouvier, M. (2011). Ligand functional selectivity and quantitative pharmacology at G protein‐coupled receptors. Expert Opinion on Drug Discovery, 6, 811–825. 10.1517/17460441.2011.586691 22651124

[bph15811-bib-0112] Stephenson, R. P. (1956). A modification of receptor theory. British Journal of Pharmacology and Chemotherapy, 11, 379–393. 10.1111/j.1476-5381.1956.tb00006.x 13383117 PMC1510558

[bph15811-bib-0113] Stout, B. D. , Clarke, W. P. , & Berg, K. A. (2002). Rapid desensitization of the serotonin(2C) receptor system: Effector pathway and agonist dependence. The Journal of Pharmacology and Experimental Therapeutics, 302, 957–962. 10.1124/jpet.302.3.957 12183652

[bph15811-bib-0114] Thomsen, A. R. B. , Plouffe, B. , Cahill, T. J. 3rd , Shukla, A. K. , Tarrasch, J. T. , Dosey, A. M. , Kahsai, A. W. , Strachan, R. T. , Pani, B. , Mahoney, J. P. , & Huang, L. (2016). GPCR‐G protein‐β‐arrestin super‐complex mediates sustained G protein signaling. Cell, 166, 907–919. 10.1016/j.cell.2016.07.004 27499021 PMC5418658

[bph15811-bib-0115] Tobin, A. B. , Totty, N. F. , Sterlin, A. E. , & Nahorski, S. R. (1997). Stimulus‐dependent phosphorylation of G‐protein‐coupled receptors by casein kinase 1α. The Journal of Biological Chemistry, 272, 20844–20849. 10.1074/jbc.272.33.20844 9252410

[bph15811-bib-0116] Tsvetanova, N. G. , & von Zastrow, M. (2014). Spatial encoding of cyclic AMP signaling specificity by GPCR endocytosis. Nature Chemical Biology, 10, 1061–1065. 10.1038/nchembio.1665 25362359 PMC4232470

[bph15811-bib-0117] Urban, J. D. , Clarke, W. P. , von Zastrow, M. , Nichols, D. E. , Kobilka, B. , Weinstein, H. , Javitch, J. A. , Roth, B. L. , Christopoulos, A. , Sexton, P. M. , Miller, K. J. , Spedding, M. , & Mailman, R. B. (2007). Functional selectivity and classical concepts of quantitative pharmacology. The Journal of Pharmacology and Experimental Therapeutics, 320, 1–13. 10.1124/jpet.106.104463 16803859

[bph15811-bib-0118] Wacker, D. , Stevens, R. C. , & Roth, B. L. (2017). How ligands illuminate GPCR molecular pharmacology. Cell, 170, 414–427. 10.1016/j.cell.2017.07.009 28753422 PMC5560499

[bph15811-bib-0119] Wehbi, V. L. , Stevenson, H. P. , Feinstein, T. N. , Calero, G. , Romero, G. , & Vilardaga, J. P. (2013). Noncanonical GPCR signaling arising from a PTH receptor‐arrestin‐Gβγ complex. Proceedings of the National Academy of Sciences of the United States of America, 110, 1530–1535. 10.1073/pnas.1205756110 23297229 PMC3557057

[bph15811-bib-0120] Whalen, E. J. , Rajagopal, S. , & Lefkowitz, R. J. (2011). Therapeutic potential of β‐arrestin‐ and G protein‐biased agonists. Trends in Molecular Medicine, 17, 126–139. 10.1016/j.molmed.2010.11.004 21183406 PMC3628754

[bph15811-bib-0121] White, K. L. , Scopton, A. P. , Rives, M. L. , Bikbulatov, R. V. , Polepally, P. R. , Brown, P. J. , Kenakin, T. , Javitch, J. A. , Zjawiony, J. K. , & Roth, B. L. (2014). Identification of novel functionally selective kappa‐opioid receptor scaffolds. Molecular Pharmacology, 85, 83–90. 10.1124/mol.113.089649 24113749 PMC3868907

[bph15811-bib-0122] Wright, S. C. , & Bouvier, M. (2021). Illuminating the complexity of GPCR pathway selectivity—Advances in biosensor development. Current Opinion in Structural Biology, 69, 142–149. 10.1016/j.sbi.2021.04.006 34048988

