# Supplementary material for: Community guidelines for GPCR ligand bias: IUPHAR review 32
Source: Br J Pharmacol. Author manuscript; Available in PMC 2022 Jul 1. (PMC7612872; doi:10.1111/bph.15811)
Supplement: Supplementary file 1 [file EMS145239-supplement-Appendix.pdf]

APPENDIX A: Experiment recommendation checklist

Choosing a reference ligand

3.1 The choice of reference ligand distinguishes benchmark-, pathway- or physiology-bias

a. Choose a reference ligand that can support the claims to be made.
b. Include multiple reference ligands, thus allowing claims about different types of ligand bias.
c. Measure the reference ligand(s) under identical conditions as the ligands tested for bias.
d. If needed, use separate reference ligands for bias and Emax.

3.2 Ligand pathway-bias (uses a pathway-balanced reference ligand)

a. Determine a pathway-balanced reference ligand in a bias plot.

3.3 Ligand physiology-bias (uses the principal endogenous ligand as reference)

a. Use the principal endogenous agonist as reference ligand.

3.4 Ligand pathway-preference (uses no reference ligand, and is not ligand bias)

a. Compare pathway ΔLog (Emax/EC_{50}) or ΔLog(τ/K_A) values, not only fold potencies.
b. Use the same or near-identical systems and assays.

Measuring at the transducer or downstream

4.1 Ligand bias measured at the transducer level

a. Use recent assays consistently profiling G proteins and arrestins (also with GRKs).
b. If possible, test all transducers for each investigated transducer family.
c. If selecting representative transducer subtypes, use the most relevant.

4.2 Ligand bias measured downstream of distinct transducers

i. Minimize differential signal amplification by measuring pathways at similar depth (estimated based on the number of upstream effectors).

4.3 Ligand bias measured downstream of converged transducer pathways

i. Avoid measuring downstream of converged pathways.
ii. If possible, dissect upstream transducer contributions using other assays.

Considering cellular system, kinetics and spatial bias

5.1 Bias may not translate across in vitro, physiological, and therapeutic systems

a. Where possible, use primary and/or disease-specific cells and evaluate potential system bias.
b. Where possible, validate the effect in a model organism using an appropriate model of efficacy, and/or genetic engineering to confirm target and pathway specificity.

5.2 Kinetics and choosing measurement time points

a. When possible, complete time courses and endpoint measurements should be made.
b. Single time points should be the physiologically most relevant or the measure maximum effect.

How to quantify ligand bias

6.1 Many models exist to quantify ligand bias

a. It is not possible to recommend a single best practice quantification method.
b. Results are more definitive when bias is quantified using multiple models.
c. Irrespective of the model, error propagation and statistics must be handled appropriately.

6.2 A minimal and a refined model to calculate a ligand bias factor

a. ΔΔLog (Emax/EC_{50}) should not be used when ligand concentration-response-curve slope factors (Hill coefficients) are not close to 1.
b. ΔΔLog(τ/K_A) values are preferred over ΔΔLog (Emax/EC_{50}) when ligand concentration-response-curve slope factors (Hill coefficients) are not close to 1.
c. Confirm that the calculated bias factors are consistent with a bias plot.

6.3 Comparing ligand bias across studies and systems (use of rank orders)

a. We recommend using ligand rank orders of bias factors (rather than quantitative bias values) for comparisons of ligand bias across studies using different experimental systems.

Special recommendations for 'tricky' ligands

7.1 Low efficacy agonists

a. Use ligand pathway rank orders.
b. If quantifying bias, use another reference agonist for Emax.

7.2 Inverse agonists

- Use another inverse agonist as the reference ligand.

7.4 Allosteric modulators affecting ligand bias

a. Quantification of allosteric modulator bias should make use of an extended operational model of allosterism.

APPENDIX B: Reporting recommendation checklist

Choosing a reference ligand

3.1 The choice of reference ligand distinguishes benchmark-, pathway- or physiology-bias

i. Report the reference ligand along with a motivation to why it was chosen.
ii. The claims should be restricted to what is supported by the chosen reference ligand.
iii. Define the bias type.

3.2 Ligand pathway-bias (uses a pathway-balanced reference ligand)

i. Document the pathway-balance of the reference ligand.

Measuring at the transducer or downstream

4.1 Ligand bias measured at the transducer level

i. Modifications of receptors, transducers or effectors must be clearly defined (e.g., tags, mutations, and chimeras).

4.2 Ligand bias measured downstream of distinct transducers

i. Report measured processes.
ii. Report the measured molecules.

4.3 Ligand bias measured downstream of converged transducer pathways

i. When pathways are truly inseparable and their contributions cannot be dissected using upstream assays, the bias may be described as a type of 'effector bias' (instead of pathway-bias) accounting for the net pathway contributions.
ii. Interpret findings in light of their relative strength for the given receptor and ligand.

Considering cellular system, kinetics and spatial bias

5.1 Bias may not translate across in vitro, physiological, and therapeutic systems

- Where a non-native system has been used, conclusions should be stated carefully.

5.2 Kinetics and choosing measurement time points

i. Report the chosen time points and the reason(s) why they were chosen.
ii. Provide data on the complete time course, if available.

5.3 Spatial bias: differing signaling efficacies across cellular compartments

i. Report the biosensors and tags used for monitoring compartment-specific signaling.
ii. Report the cell types used in assays.
iii. Report ligands with altered characteristics, e.g., from chemical modification.

How to quantify ligand bias

6.2 A minimal and a refined model to calculate a ligand bias factor

i. Report ligand Emax, EC_{50} and τ/K_A values and system maximum response, Em.
ii. Report the ligand concentration-response-curve slope factors (Hill coefficients).
iii. Report a bias plot for biased ligands.

Special recommendations for 'tricky' ligands

7.3 Agonist and antagonist across pathways ('modality bias')

i. Ligand bias with opposite modalities across pathways can be described as a non-quantitative term, 'modality bias'.
ii. Alternatively, it can be approximated by measuring an affinity to limit bias or describe it in a 'bias is larger than' relationship.

7.4 Allosteric modulators affecting ligand bias

i. Report both the allosteric modulator and orthosteric ligand.

Publication and database deposition

8.1 Unambiguous description of ligand bias

i. Define the ligand evoking bias and its system in a clear sentence.
ii. Tabulate the experimental details required for unambiguous description (Table 3)
iii. Deposit biased ligands in a database.

APPENDIX C: Terminology cheat sheet

Definitions of pathways and of bias types

2.1 Pathway definition and modulation

Transducer: proteins that bind directly to an activated receptor to initiate, facilitate or modulate downstream events. This includes G proteins, GRKs and arrestins.

Effector: Signaling protein located downstream in a transducer's pathway.

Modulator: Proteins or molecules that do not mediate, but modulate signaling of a receptor, transducer or effectors. (RAMPs, GEFs, GAPs, RGSes, NO, cholesterol, other lipids etc.

Second messenger: Small molecules or ions directly controlled by the effectors. Examples include cAMP, calcium, etc.

Pathway: A pathway is named after a transducer protein, or family thereof, that binds to GPCRs.

2.2 Ligand bias definition and distinction from system bias

Ligand bias: Ligand-dependent preferential receptor activation so that one over other transducer pathways in a given cellular system and relative to a reference ligand is induced.

System bias: Bias due to differences in the cellular system, including so called 'tissue bias'.

Functional selectivity: Functional selectivity is the observed response combining ligand- and system-bias.

Observational bias: An artificial bias introduced by the experimental setup.

Choosing a reference ligand

3.1 The choice of reference ligand distinguishes benchmark-, pathway- or physiology-bias

Biased ligand: Ligand preferentially activating one receptor transducer pathway in a given cellular system and relative to a reference ligand. Ligand bias is a property of not just a ligand, but of a ligand, pathway pair and receptor in combination, and only valid within the specific investigated system. Therefore, the term 'biased ligand' should only be used if explicitly defined, and not be construed to represent a ligand-only property. A recommended definition is included in section 'Unambiguous description of ligand bias', which provides one-sentence and table templates for reporting.

Reference ligand for bias: The ligand that is, by definition, unbiased. The bias of any other tested ligands is quantified relative to this reference.

Reference ligand for E_{max}: A separate reference ligand for the full receptor response (maximum efficacy, E_{max}).

Unbiased ligand: A ligand that stimulates pathways in a manner indistinguishable from the reference ligand.

3.2 Ligand pathway-bias (uses a pathway-balanced reference ligand)

Balanced ligand: Has indistinguishable or very similar signaling through compared pathways.

Ligand pathway-bias: Ligand bias that is measured relative to a balanced reference ligand and therefore has the meaning that signaling is predominant via one pathway.

3.3 Ligand physiology-bias (uses the principal endogenous ligand as reference)

G protein selectivity: The repertoire of G proteins that a receptor can engage. The term 'natural bias' is self-contradictory and should not be used.

Ligand physiology-bias: Ligand bias relative to a receptor's principal endogenous agonist, which therefore bears the meaning that signaling differs from the physiological.

3.4 Ligand pathway-preference (uses no reference ligand, and is not ligand bias)

Pathway-preference: A ligand's differential activity across pathways (e.g. pathway ΔLog (Emax/EC50) values), but without comparison to a reference ligand.

5.2 Kinetics and choosing measurement time points

Considering cellular system, kinetics and spatial bias

Temporal effect: The effect influencing a measured response due to the choice of time point at which a response is recorded.

5.3 Spatial bias: differing signaling efficacies across cellular compartments

Spatial/location bias: The observation of biased GPCR signaling through the same transducer in different locations that results in distinct signaling responses.

Special recommendations for 'tricky' ligands

7.3 Agonist and antagonist across pathways ('modality bias')

Modality bias: Ligand with efficacy in only one of compared pathways (neutral antagonist or inverse agonist in others).
